# Identification and characterization of the cytosine-5 DNA methyltransferase gene family in *Salvia miltiorrhiza*

**DOI:** 10.7717/peerj.4461

**Published:** 2018-03-05

**Authors:** Jiang Li, Caili Li, Shanfa Lu

**Affiliations:** Institute of Medicinal Plant Development, Chinese Academy of Medical Sciences & Peking Union Medical College, Beijing, China

**Keywords:** Yeast extract, *Salvia miltiorrhiza*, Methyl jasmonate, C5-MTase, Cytosine DNA methylation, Salicylic acid

## Abstract

Cytosine DNA methylation is highly conserved epigenetic modification involved in a wide range of biological processes in eukaryotes. It was established and maintained by cytosine-5 DNA methyltransferases (C5-MTases) in plants. Through genome-wide identification, eight putative *SmC5-MTase* genes were identified from the genome of *Salvia miltiorrhiza*, a well-known traditional Chinese medicine material and an emerging model medicinal plant. Based on conserved domains and phylogenetic analysis, eight *SmC5-MTase* genes were divided into four subfamilies, including *MET*, *CMT*, *DRM* and *DNMT2*. Genome-wide comparative analysis of the *C5-MTase* gene family in *S. miltiorrhiza* and *Arabidopsis thaliana*, including gene structure, sequence features, sequence alignment and conserved motifs, was carried out. The results showed conservation and divergence of the members of each subfamily in plants. The length of *SmC5-MTase* open reading frames ranges widely from 1,152 (*SmDNMT2*) to 5,034 bp (*SmMET1*). The intron number of *SmC5-MTases* varies between 7 (*SmDRM1*) and 20 (*SmCMT1* and *SmCMT2b*). These features were similar to their counterparts from *Arabidopsis*. Sequence alignment and conserved motif analysis showed the existence of highly conserved and subfamily-specific motifs in the C5-MTases analyzed. Differential transcript abundance was detected for *SmC5-MTases*, implying genome-wide variance of DNA methylation in different organs and tissues. Transcriptome-wide analysis showed that the transcript levels of all *SmC5-MTase* genes was slightly changed under yeast extract and methyl jasmonate treatments. Six *SmC5-MTases*, including *SmMET1*, *SmCMT1*,* SmCMT2a*, *SmCMT2b*, *SmCMT3* and* SmDRM1*, were salicylic acid-responsive, suggesting the involvement of *SmC5-MTase*s in salicylic acid-dependent immunity. These results provide useful information for demonstrating the role of DNA methylation in bioactive compound biosynthesis and Dao-di herb formation in medicinal plants.

## Introduction

Cytosine DNA methylation, a dominating epigenetic modification mechanism, plays vital roles in gene expression regulation, transposon silencing, gene imprinting, and X chromosome inactivation (Chan, Henderson & Jacobsen, 2005; Law & Jacobsen, 2010). It is widespread in eukaryotes and displays distinct characteristics in specific species (Law & Jacobsen, 2010). In animals, cytosine methylation mostly occurs in CG sites with the exception of embryonic stem cells and neurons (Lister et al., 2009; Lister et al., 2013). DNA methylation in plants usually occurs at CG sites. It also occurs at CHG and CHH sites, where H represents any nucleotide except G (Cokus et al., 2008).

DNA methylation of each sequence context in plants can be maintained or established *de novo* through distinct mechanisms (Niederhuth & Schmitz, 2014). There are two main types of DNA methyltranferases (C5-MTases) involved in DNA methylation maintenance. DNA methyltransferase 1 (MET1), homologous to mammalian Dnmt1, maintains the CG context methylation status through recognizing the hemimethylated CG sites and methylating the newly synthesized DNA strand during DNA replication (Finnegan & Dennis, 1993; Kankel et al., 2003; Law & Jacobsen, 2010). Chromomethylase 3 (CMT3), a plant-specific DNA methyltransferase, maintains CHG context methylation through a self-reinforcing loop interacted with dimethylation of histone 3 on Lysine 9 (H3K9me2) (Du et al., 2012). *De novo* methylation is mainly established by RNA-directed DNA methylation (RdDM), in which domains-rearranged methyltransferases (DRMs) are guided to target locus to direct methylation of all three sequence contexts via 24-nucleotide short interfering RNAs (Cao & Jacobsen, 2002a; Cao & Jacobsen, 2002b; Law & Jacobsen, 2010). Although DNA methylation of all sequence contexts can be established *de novo* by RdDM, CHH context is the primary target of RdDM. In addition to RdDM, asymmetrical CHH methylation may also be established and maintained in H3K9me2 regions by CHROMOMETHYLASE 2 (CMT2), another CMT subfamily member (Stroud et al., 2014; Zemach et al., 2013). CMT2- and RdDM-mediated DNA methylation targets CHH sites at different position of the genome (Gent et al., 2013; Stroud et al., 2014; Zemach et al., 2013). The status of cytosine DNA methylation is dynamic. The balance of methylation and non-methylation is also regulated by passive or active DNA demethylation. Passive DNA demethylation occurs in newly synthesized DNA strand by dysfunction of DNA methyltransferase, whereas active demethylation is an outcome of replacement of methylated cytosine with nonmethylated cytosine under the catalysis of DEMETER-like DNA glyscosylases (Law & Jacobsen, 2010; Zhang & Zhu, 2012; Zhu, 2009).

Increasing evidence demonstrates that DNA methylation is involved in the regulation of many important biological processes, including leaf growth (Candaele et al., 2014), seed development (Xing et al., 2015), hybrid vigor (Kawanabe et al., 2016), fruit ripening (Liu et al., 2015; Zhong et al., 2013), and secondary metabolism (Conde et al., 2017). Due to the importance of DNA methylation, *C5-MTase* genes have been widely identified from various plant species, such as *Arabidopsis* (Ashapkin, Kutueva & Vanyushin, 2016), rice (Ahmad et al., 2014), tomato (Cao et al., 2014), soybean (Garg et al., 2014), maize (Qian et al., 2014), peanut (Wang et al., 2016), and globe artichoke (Gianoglio et al., 2017). Based on conserved domain and phylogenetic analysis, *C5-MTase* genes can be divided into the four DNA methyltransferase subfamilies, including *MET*, *CMT*, *DRM*, and *DNMT2*. The function of a few *C5-MTases* has been analyzed. *AtMET1* was found to regulate plant morphology and flowering time through the maintenance of CG methylation in *Arabidopsis* (Finnegan, Peacock & Dennis, 1996). The *Arabidopsis drm1drm2cmt3* triple mutant exhibited pleiotropic developmental defects, including developmental retardation, plant size reduction, and partial sterility (Cao & Jacobsen, 2002a). Null function of *OsMET1-2* caused abnormal seed development and swift seedling lethality in rice (Hu et al., 2014). Functions of the other *C5-MTases* are largely unknown.

*Salvia miltiorrhiza*, a member of the Labiatae family, has been widely and successfully used to treat dysmenorrheal, coronary heart diseases and amenorrhoea (Cheng, 2006a; Cheng, 2006b). It is also an emerging model system for traditional Chinese medicine (TCM) studies (Li et al., 2009; Ma et al., 2012; Wang, Morris-Natschke & Lee, 2007; Xu et al., 2015). The genome of *S. miltiorrhiza* has been decoded recently and abundant transcriptome data are available (Xu et al., 2016; Zhang et al., 2015). Many bioactive compound biosynthesis-related genes have been identified (Li & Lu, 2014; Ma et al., 2012; Xu et al., 2015; Zhang et al., 2014). However, little is known about DNA methylation-related genes in *S. miltiorrhiza*. With long-term goal to explore biological role of DNA methylation in medicinal plants and to test whether DNA methylation is involved in the regulation of bioactive compound biosynthesis and Dao-di herb formation, we carried out genome-wide identification and characterization of the *SmC5-MTase* gene family in *S. miltiorrhiza*.

## Materials & Methods

### *SmC5-MTase* gene identification

Sequences of 11 *Arabidopsis* AtC5-MTase proteins were downloaded from the *Arabidopsis* Information Resource (TAIR, http://www.arabidopsis.org). *Arabidopsis* AtC5-MTase proteins were blast-analyzed against the *S. miltiorrhiza* 99-3 whole genome sequence using tBLASTn algorithm (Altschul et al., 1997; Xu et al., 2016). *S. miltiorrhiza C5-MTase* gene models were predicted from retrieved genomic DNA sequences through alignment with *C5-MTase* genes from other plants and *S. miltiorrhiza* transcriptome data (http://www.ncbi.nlm.nih.gov/sra) using the BLASTx and BLASTn program, respectively (http://www.ncbi.nlm.nih.gov/blast/). Obtained raw genomic sequences, open reading frame (ORF) sequences and deduced protein sequences were listed in Data S1.

### Gene structure determination, protein sequence analysis and phylogenetic tree construction

Gene structures were determined on the Gene Structure Display Server (GSDS 2.0, http://gsds.cbi.pku.edu.cn/index.php) using the coding sequences and the corresponding genomic sequences as inputs. Protein amino acid number, molecular weight (Mw) and theoretical isoelectric point (p*I*) were analyzed using the EXPASY PROTOPARAM tool (http://www.expasy.org/tools/protparam.html). Multiple sequence alignment was carried out using ClustalW (Larkin et al., 2007). Conserved motifs were determined using the MEME suite (Bailey et al., 2009). The neighbor-joining (NJ) tree was constructed using MEGA version 7.0 (Kumar, Stecher & Tamura, 2016). Node robustness was detected using the bootstrap method (1,000 replicates).

### RNA extraction and quantitative real-time reverse transcription polymerase chain reaction (qRT-PCR)

Roots, stems, leaves and flowers of 2-year-old *S. miltiorrhiza* (line 99-3) plants grown in a field nursery at the Institute of Medicinal Plant Development, Chinese Academy of Medical Sciences, were collected in August and stored in liquid nitrogen until use. Total RNA was extracted from three biological replicates for each tissue using the Quick RNA isolation kit (Huayueyang Biotechnology, Beijing, China). Each biological replicate represents independent single plant. RNA integrity and quantity were analyzed using agarose gel and NanoDrop 2000C Spectrophotometer (Thermo Scientific, Waltham, MA, USA), respectively. Total RNA was reverse-transcribed using the PrimeScript™  RT reagent kit (TaKaRa, Japan). qRT-PCR primers listed in Table S1 were designed using Primer3 (http://frodo.wi.mit.edu/primer3/). qRT-PCR analysis was conducted in triplicate on the CFX96™ real-time PCR detection system (Bio-Rad, Hercules, CA, USA) for each biological replicate using SYBR premix Ex Taq™ kit (TaKaRa, Shiga, Japan) as described (Ma et al., 2012). The 2^−ΔΔ*Ct*^ method was used to evaluate relative transcript levels (Livak & Schmittgen, 2001). Differential transcript abundance among tissues was determined by the one-way ANOVA (analysis of variance) method using IBM SPSS 20 software (IBM Corporation, Armonk, NY, USA).

### RNA-seq data and bioinformatic analysis

Transcriptome data of periderm, phloem and xylem from *S. miltiorrhiza* roots was downloaded from SRA database of NCBI (SRA accession number SRR1640458) (Xu et al., 2015). RNA-seq reads from *S. miltiorrhiza* hairy roots treated with yeast extract (YE) and methyl jasmonate (MeJA) were downloaded from SRA database under the accession number SRP111399 (Zhou et al., 2017). RNA-seq data for *S. miltiorrhiza* cell cultures treated with salicylic acid (SA) were downloaded from SRA database under the accession number SRX1423774 (Zhang et al., 2016). Differential transcript abundance was analyzed using TopHat2.0.12 and Cufflinks2.2.1 (Trapnell et al., 2012). Heat maps were created using R statistical software (Le Meur & Gentleman, 2012).

## Results

### Genome-wide identification and sequence feature analysis of *SmC5-MTase* genes

**Figure 1:**
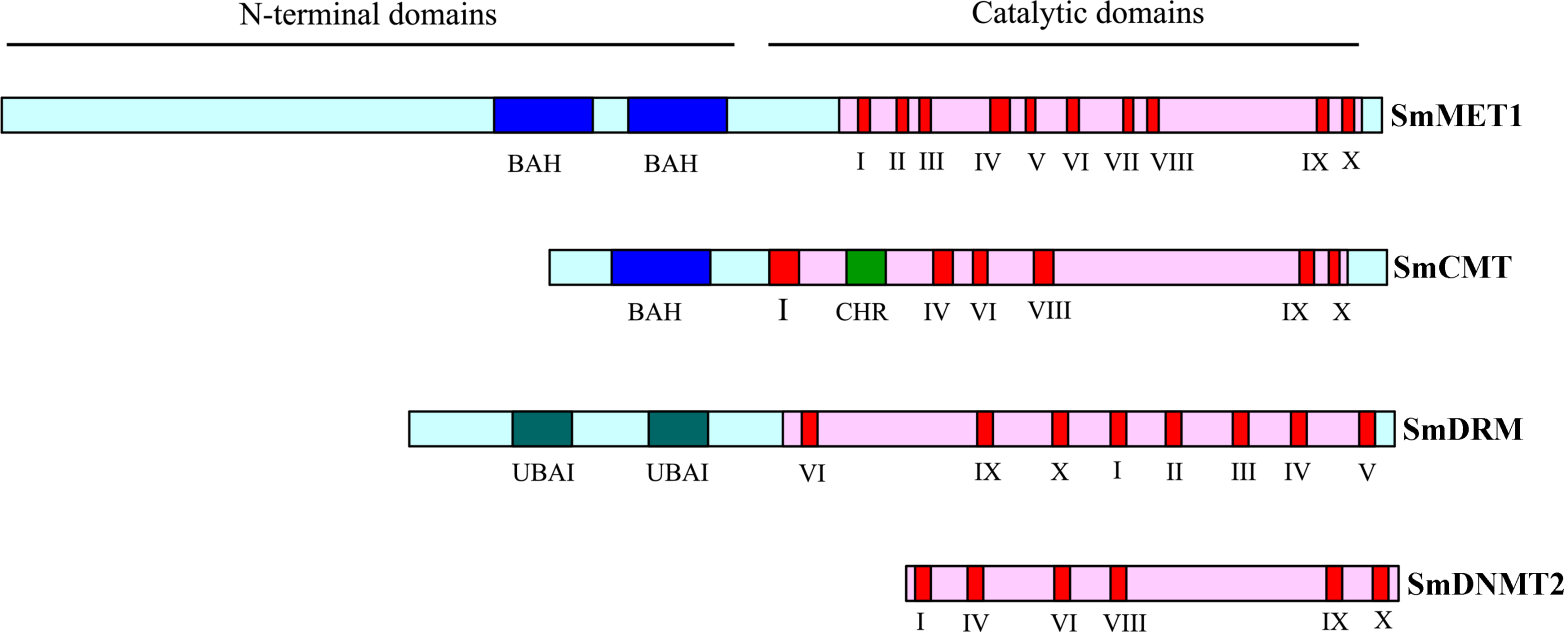
Schematic structures of SmC5-MTase proteins.

Blast analysis of eleven *Arabidopsis* cytosine DNA methyltransferase proteins against the whole *S. miltiorrhiza* 99-3 genome sequence resulted in the identification of eight *SmC5-MTase* genes. The number of *SmC5-MTase* genes in *S. miltiorrhiza* is comparable with that in other plants, such as *Arabidopsis* (Ashapkin, Kutueva & Vanyushin, 2016), rice (Ahmad et al., 2014) and maize (Qian et al., 2014), which contain eleven, ten and eight *C5-MTases*, respectively. The presence and distribution of conserved domains and motifs was confirmed by PFAM analysis of protein sequences (Fig. 1). Although the conserved catalytic C-terminal domains with conserved motifs are ubiquitous in all identified SmC5-MTase proteins, the N-terminals have diverse combinations of conserved domains among subfamilies (Fig. 1). It allows us to easily name newly identified genes. C5-MTases with two bromo adjacent homology (BAH) domains in the N-terminal belong to the MET subfamily, whereas C5-MTases having only one BAH domain belong to the CMT subfamily. C5-MTases containing ubiquitin associated (UBA) domains in the N-terminal were classified as the DRM subfamily. In addition, C5-MTases without N-terminal conserved domain belong to the DNMT2 subfamily. Accordingly, the eight *SmC5-MTase* genes were named *SmMET1*, *SmCMT1*, *SmCMT2a*, *SmCMT2b*, *SmCMT3*, *SmDRM1*, *SmDRM2*, and *SmDNMT2*, respectively (Table 1).

**Figure 2:**
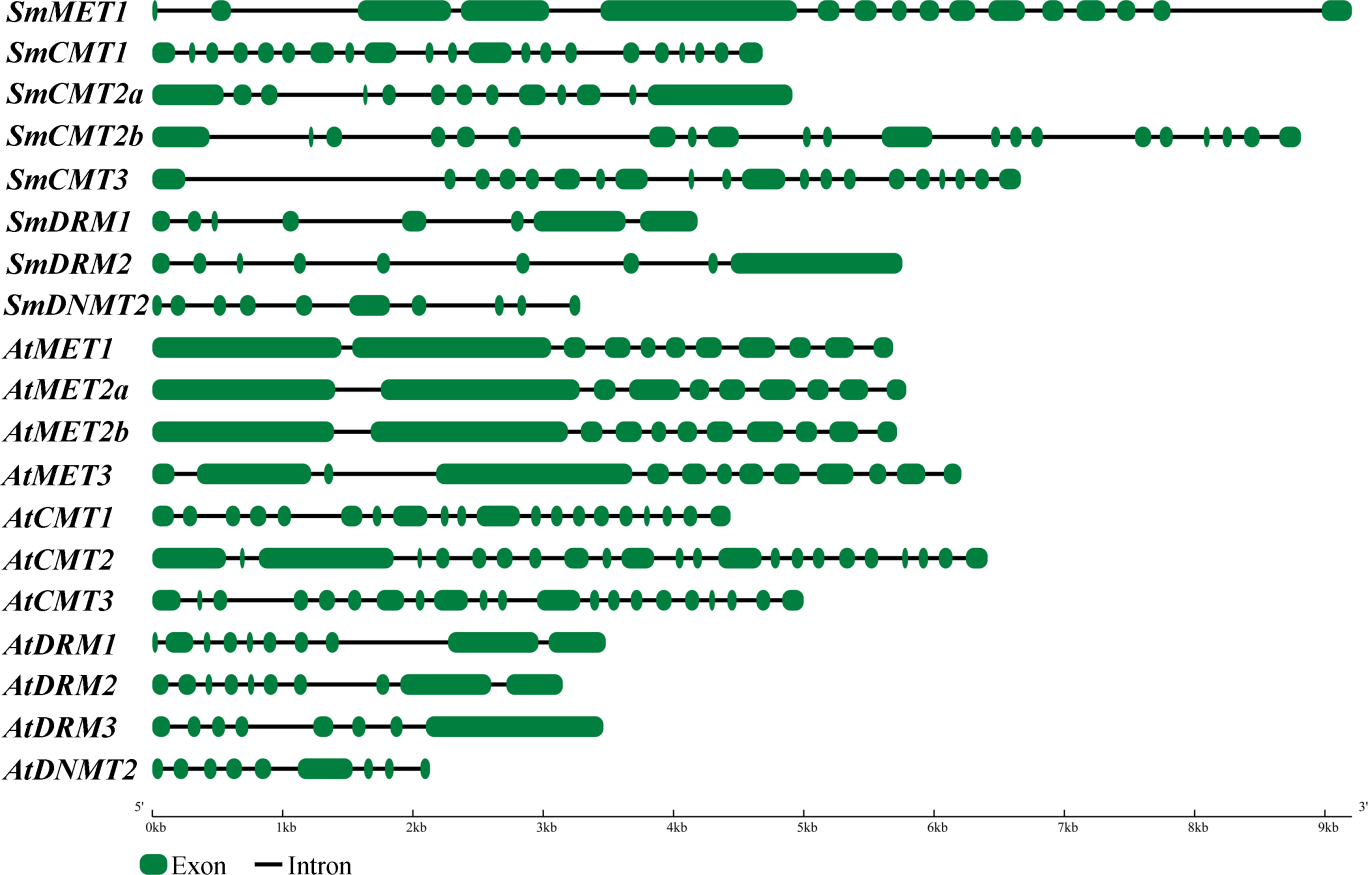
Gene structures of *C5-MTases.* in *S.miltiorrhiza* and *Arabidopsis*.

The length of ORFs of *SmC5-MTase* genes varies from 1,152 bp to 5,034 bp (Table 1). The deduced proteins vary between 383 amino acids and 1,677 amino acids, respectively. The theoretical isoelectric point (p*I*) varies from 4.89 (SmCMT3) to 8.03 (SmCMT2a) and the molecular weight (Mw) varies from 43.7 kDa (SmDNMT2) to 189.4 kDa (SmMET1) (Table 1). These sequence features were similar to their counterparts in *Arabidopsis*. On the other hand, the genomic DNA length of *SmC5-MTase* genes was obviously greater than their *Arabidopsis* counterparts (Table 1). Gene structure analysis showed that introns were responsible for the enlargement of *SmC5-MTase* genes (Fig. 2). The underlying mechanism of intron size expansion is unknown.

**Table 1:** Sequence features and intron numbers of C5-MTases in *S. miltiorrhiza* and *Arabidopsis*.

Gene name	Gene model	Gene length	No. of intron	ORF (bp)	Protein (aa)	MW (kDa)	*p*I
*SmMET1*	MG602207	9,207	15	5,034	1,677	189.4	7.54
*SmCMT1*	MG602211	4,685	20	2,448	815	91.8	6.30
*SmCMT2a*	MG602209	4,913	12	2,874	957	107.7	8.03
*SmCMT2b*	MG602210	8,816	20	2,808	935	105.3	6.97
*SmCMT3*	MG602208	6,666	19	2,460	819	91.6	4.89
*SmDRM1*	MG602212	4,185	7	1,830	609	68.6	4.97
*SmDRM2*	MG602213	5,757	8	2,070	689	76.5	5.10
*SmDNMT2*	MG602214	3,284	9	1,152	383	43.7	5.61
*AtMET1*	AT5G49160.1	5,686	10	4,605	1,534	172.4	5.97
*AtMET2a*	AT4G14140.2	5,787	9	4,638	1,545	174.8	6.09
*AtMET2b*	AT4G08990.1	5,716	10	4,539	1,512	171.2	5.79
*AtMET3*	AT4G13610.1	6,210	12	4,215	1,404	160.2	7.24
*AtCMT1*	AT1G80740.1	4,438	19	2,376	791	89.2	5.99
*AtCMT2*	AT4G19020.1	6,411	22	3,888	1,295	145.0	8.64
*AtCMT3*	AT1G69770.1	4,999	20	2,520	839	94.9	6.57
*AtDRM1*	AT5G15380.1	3,480	9	1,875	624	70.9	5.16
*AtDRM2*	AT5G14620.1	3,150	9	1,881	626	70.4	5.12
*AtDRM3*	AT3G17310.2	3,462	7	2,133	710	79.7	4.87
*AtDnmt2*	AT5G25480.1	2,131	8	1,152	383	43.7	5.97

### Sequence alignment and conserved motif analysis in C5-MTases

C5-MTases contain N- and C-terminal domains, which are necessary for establishment and maintenance of cytosine DNA methylation at distinct sequence contexts. In order to understand the conservation and divergence of these conserved domains in SmC5-MTases, multiple sequence alignment was performed for C5-MTases from *S. miltiorrhiza* and *Arabidopsis*. Four highly conserved motifs, including I, IV, VI and X (Malone, Blumenthal & Cheng, 1995), were found in the C-terminal methyltransferase domain of all C5-MTases analyzed (Figs. S1–S5). Among them, motifs I and X are involved in S-adenosyl-L-methionine (AdoMet) binding, motif IV has the proline-cysteine doublet acting as the functional catalytic site, and motif VI has a glutamic acid involved in target cytosine binding (Malone, Blumenthal & Cheng, 1995). In addition to the highly conserved motifs, various less conserved motifs were found in the C-terminal domain (Figs. S1–S5). These motifs could be associated with gene-specific functions. Compared with the C-terminal domain, the N-terminal domain is more diverse. The N-terminal of SmMET1 contains two replication foci domains (RFD) and two BAH domains (Fig. S1). Except for SmCMT2a, which lacks the Chr domain, the N-terminal of SmCMTs contains a BAH domain and a chromo (Chr) domain (Figs. S2–S3). The N-terminal of SmDRMs has two conserved UBA domains (Fig. S4). The divergence of the N-terminal domains could be important for distinct roles of different SmC5-MTases.

**Figure 3:**
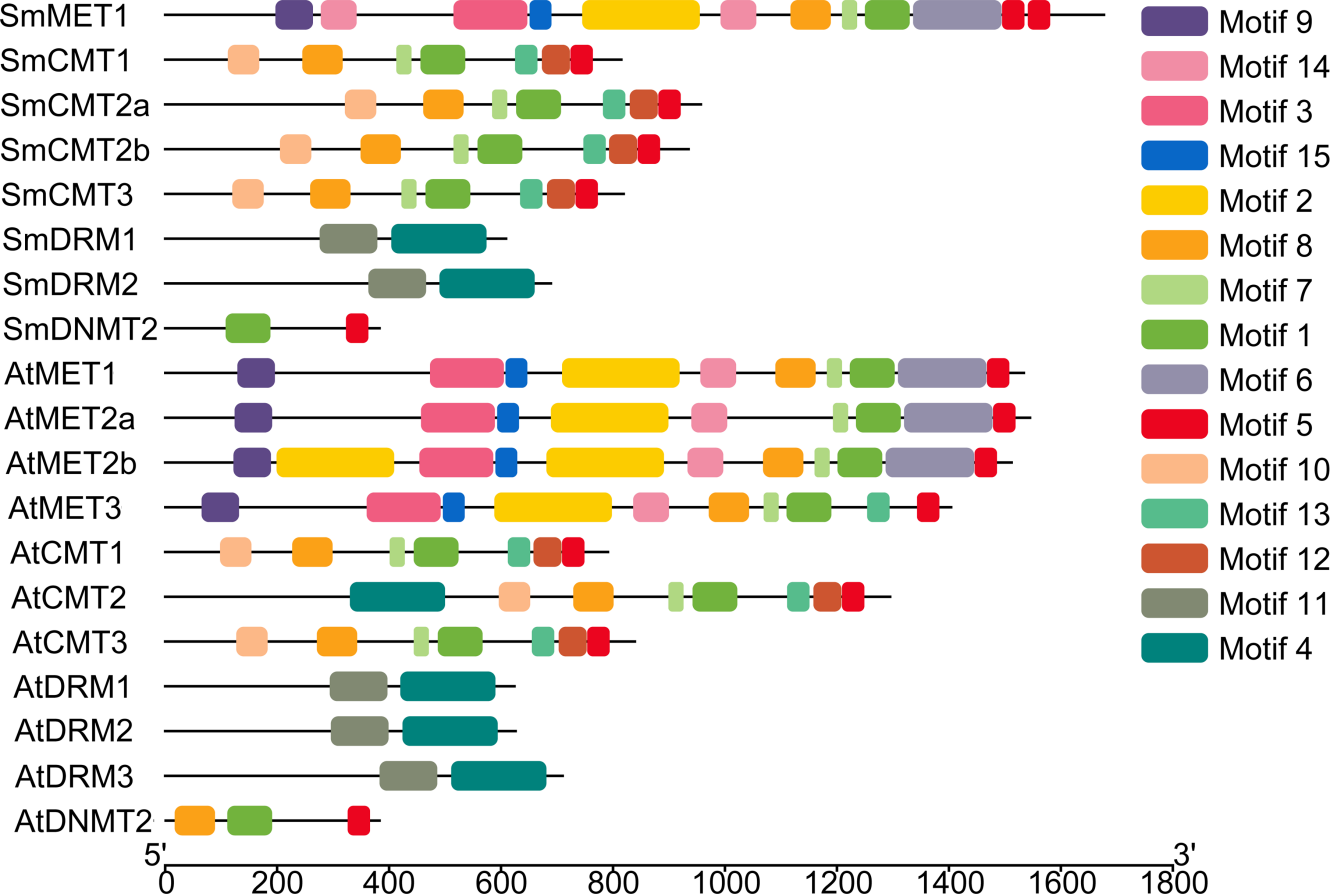
Conserved motifs of *S. miltiorrhiza* and *Arabidopsis* C5-MTase proteins identified using the MEME search tool.

To further explore sequence conservation and divergence of SmC5-MTases, MEME motif search tool was used to identify conserved motifs in SmC5-MTases and AtC5-MTases. A total of 15 conserved motifs were identified. The length of motifs ranges from 28 to 100 amino acids (Table 2). The number of motifs in each C5-MTase varies between 2 and 10. Motifs 1, 4, 5, 6, 7, 8, 12 and 13 are located in the C-terminal DNA methyltransferase domain (Fig. 3). Among them, motifs 1, 5, 7 and 8 are highly conserved in 12–14 C5-MTases, whereas motifs 4, 6, 12 and 13 are conserved in less than ten C5-MTases. The other seven, including motifs 2, 3, 9, 10, 11, 14 and 15, are located in the N-terminal regions of C5-MTases. Among them, motifs 2, 3, 9, 14 and 15 only exist in the MET subfamily. Motifs 2 and 14 correspond to two BAH domains, whereas motifs 3 and 9 are located in the two RFD domains. Motifs 10 and 11 only exist in the CMT and the DRM subfamily, respectively. Motif 10 is specific to the BAH domain of the CMT subfamily, however motif 11 is not the UBA domain of the DRM subfamily (Fig. 3). Motifs commonly existing in C5-MTases are probably associated with the conserved functions of C5-MTases, whereas those specific to a few C5-MTases seem to be related to gene-specific functions.

**Table 2:** Consensus sequences of fifteen motifs identified in *S. miltiorrhiza* and *Arabidopsis* C5-MTases.

Motif	Width	Best possible match
1	81	FLDIVDYLKPKYVLMENVVDFVRFNKGQLFRYALASLLEMGYQVRFGIMAAGAYGLPQFRKRVFIWAAAPEEVLPZWPLPT
2	100	EIKWDGEILGKTSAGEPLYGRALVGGEKVVVGGAVILEVDDPDETPLIYFVEYMFESSDHSKMLHGKLLQRGSETVLGTAANERELFLTNECLTVQLKDI
3	100	MIFVSJRTDMAWYRLGKPSKQYAPWFEPVLKTVRVGISILTMLKRESRVAKLSYADVIKRLCGLEENDKAYISSKLLDVERYVVVHGQIILQLFEEYPDD
4	100	VQRYVLEQCKKWNLVWVGKNKLAPLEPDELEKILGFPKNHTRGGGISLTERYKSLGNSFQVDTVAYHLSVLKPJFPNGINVLSLFSGIGGAEVALHRLGI
5	41	LHPEQDRVLSVRECARLQGFPDSYKFSGTIKEKYRQIGNAV
6	100	PGLKISLPRGLHYDAVRNTKFGAPFRPITVRDTIGDLPPVENGESKTNKEYKTTPVSWFQKKIRGNMSVLTDHICKGLNELNLIRCKKIPKRPGADWRDL
7	28	ILPLPGQVDFICGGPPCQGISGYNRFRB
8	73	LLDLYSGCGAMSTGLCJGAKJSGVKLVTKWAVDJNSYACESLKLNHPETQVRNEAAEDFLDLLKEWEKLCKKY
9	68	GVRFQSFGRVENWNISGYEDGSPVIWISTALADYDCRKPAKKYKKJYDYFFEKACACVEVYKSLSKNP
10	57	FIGKIVEFFETTDGESYFRVQWFYRAEDTVIKNQASLIDKKRVFYSEIMBDNPLDCJ
11	100	HRSLPDKARGPPYFYYENVALTPKGVWAKISRFLYDIQPEFVDSKYFSAAARKRGYIHNLPIENRFQIQPPPPLTIQEAFPLSKRWWPSWDKRKKLNCIL
12	51	ERVKLPSGKPLVPDYALSFEDGKSKKPFGRLWWDETVPTVVTRPEPHNQAI
13	41	LYDHRPLKLNEDDYERVCQIPKRKGANFRDLPGVIVGPDNV
14	65	LKESRKASKASFQVKVRRFYRPEDISAEKAYASDIQELYYSQDTYILPPEAIZGKCEVRKKSDMP
15	40	NLNPRAGIAPVVSKMKAMQATTTRLVNRIWGEFYSIYSPE

### Phylogenetic analysis of C5-MTases in *S. miltiorrhiza* and other plant species

**Figure 4:**
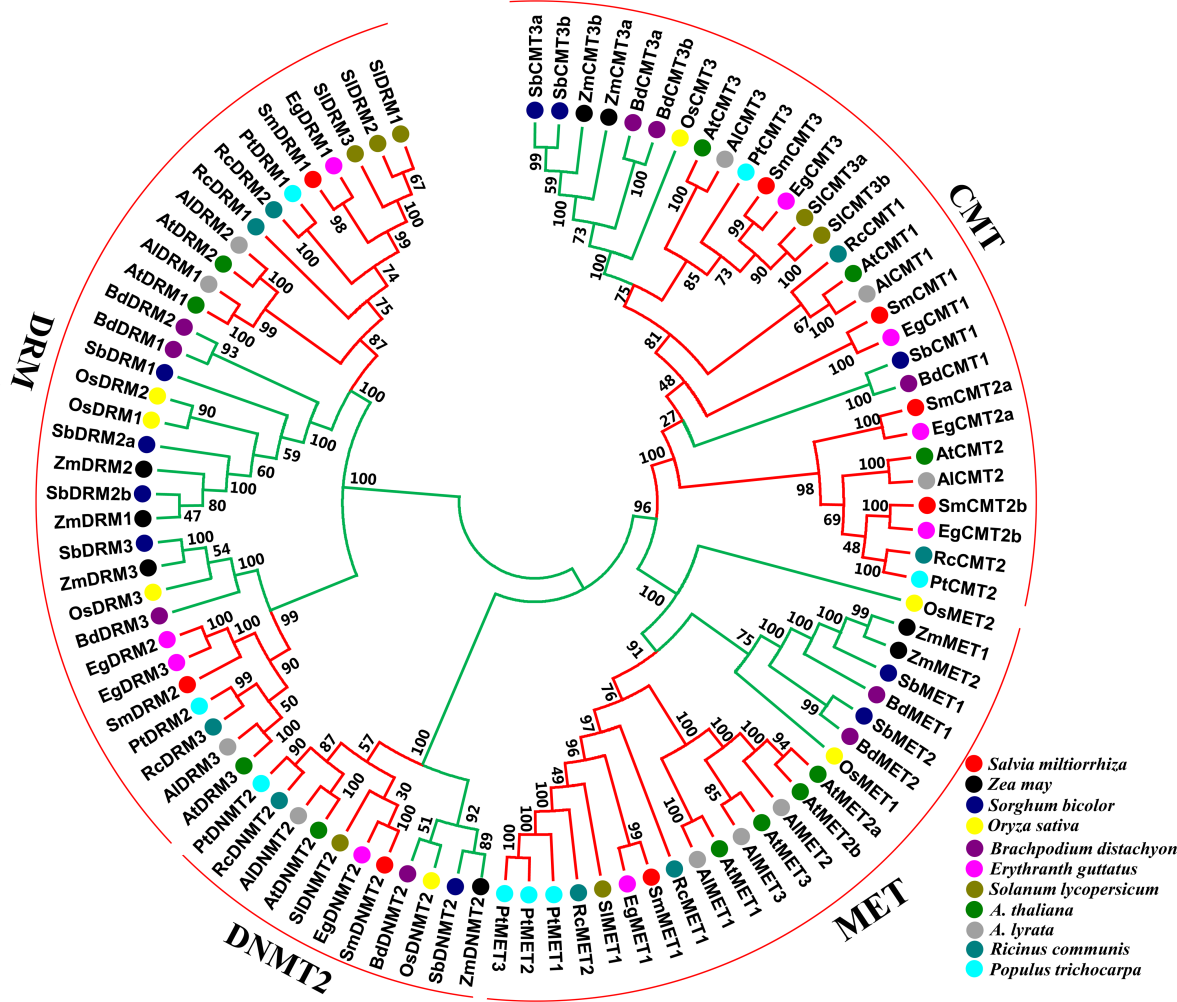
Phylogenetic analysis of C5-MTase proteins from eleven plant species. Dicots and monocots are shown with red and green colors, respectively.

To understand the phylogenetic relationships and evolutionary history of plant C5-MTases, an unrooted neighbor-joining phylogenetic tree was constructed for full-length protein sequences of C5-MTases from *S. miltiorrhiza* and other 10 plant species (Table  S2). Four subfamilies, including MET, DNMT2, CMT and DRM, are exhibited in the tree (Fig. 4). It is consistent with previous studies on other plants (Cao et al., 2014; Qian et al., 2014), confirming the domain based classification and nomenclature. The MET subfamily contains SmMET1 and can be further divided into dicot and monocot groups. The CMT subfamily can be grouped into three clades, including CMT1, CMT2 and CMT3. Among them, CMT1 and CMT3 can be further divided into dicot and monocot groups, whereas CMT2 exists in dicots only. The DRM subfamily can be divided into two dicot and two monocot groups. Similarly, The DNMT2 subfamily can be divided into dicot and monocot groups. The results imply that each subfamily is phylogenetically monophyletic and includes dicot and monocot evolutionary lineages. Taken together, each of the MET and the DNMT2 subfamilies contains a SmC5-MTase, whereas the CMT and the DRM subfamilies include four and two SmC5-MTases, respectively. SmC5-MTases have close phylogenetic relationships with the counterparts from *Erythranthe guttatus* (Fig. 4). It is consistent with the fact that both *S. miltiorrhiza* and *E. guttatus* are members of the Lamiales order.

### Transcript abundance analysis of *SmC5-MTase* genes in *S. miltiorrhiza*

**Figure 5:**
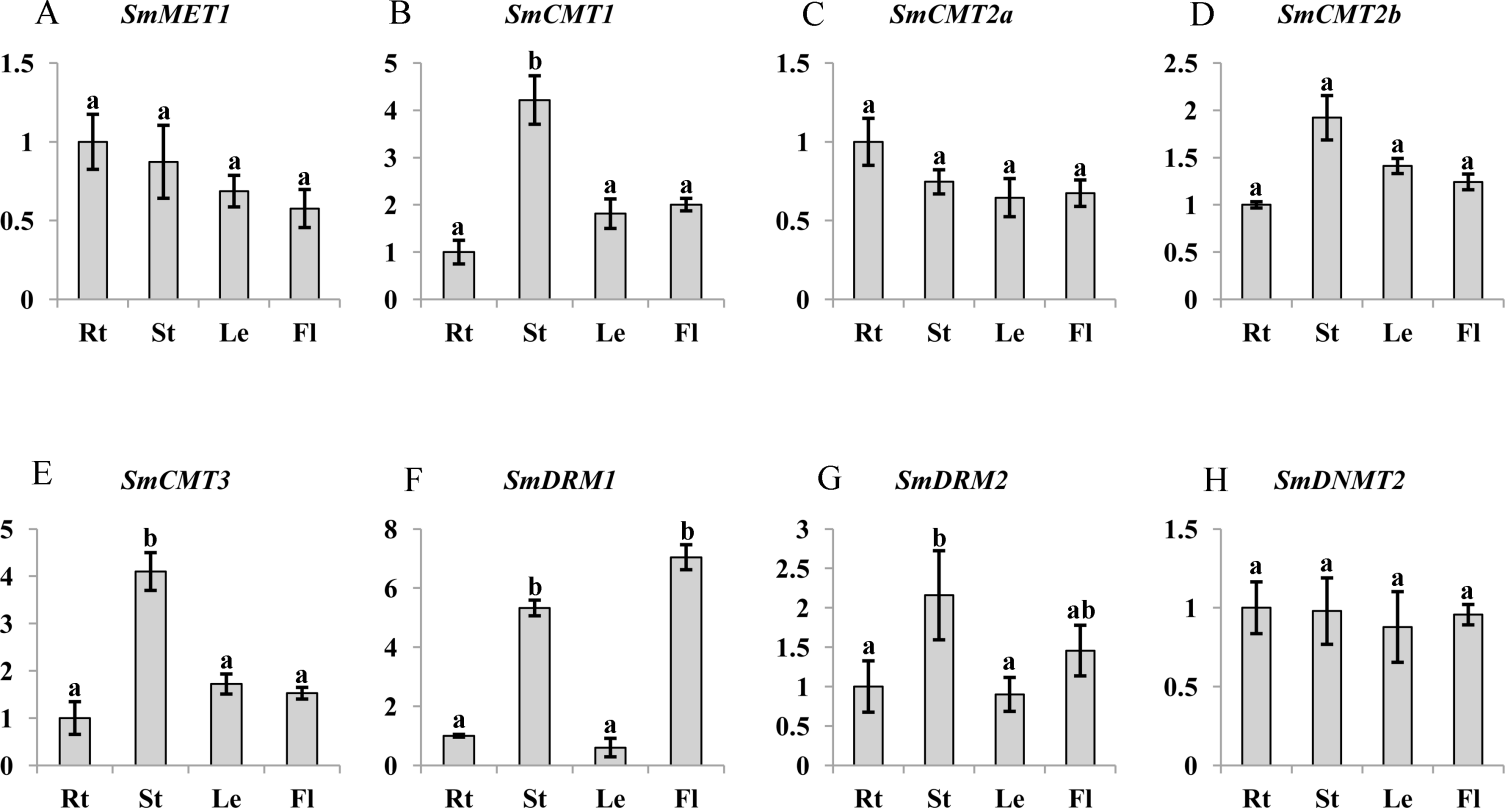
Transcript abundance of *SmMET1* (A), *SmCMT1* (B), *SmCMT2a* (C), *SmCMT2b* (D), *SmCMT3* (E), *SmDRM1* (F), *SmDRM2* (G), and *SmDNMT2* (H) in roots (Rt), Stems (St), leaves (Le) and flowers (Fl) of *S. miltiorrhiza*. Transcript level was analyzed using quantitative real time RT-PCR. *SmUBQ10* was used as a reference. qRT-PCR was performed in triplicates for each independent biological replicate. Transcript levels in roots were arbitrarily set to 1 and the levels in other tissues were given relative to this. One-way ANOVA was performed using IBM SPSS 20 software. *P* < 0.05 was considered statistically significant and represented by different letters. Error bars indicate the standard deviations for three biological replicates.

*C5-MTases* are important for plant growth and development (Cao & Jacobsen, 2002a; Finnegan, Peacock & Dennis, 1996; Hu et al., 2014). To preliminarily understand the function of *SmC5-MTases*, transcript abundance of genes in roots, stems, leaves and flowers of 2-year-old, field nursery-grown *S. miltiorrhiza* plants were analyzed. Transcripts of all eight *SmC5-MTase* genes could be detected in organs analyzed, although differential transcript levels were observed. *SmMET1*, which may be responsible for CG methylation maintenance, shows relatively uniform transcript levels in roots, stems, leaves and flowers of *S. miltiorrhiza* (Fig. 5). It is consistent with the transcript abundance patterns of *AtMET1* in vegetative organs (Ashapkin, Kutueva & Vanyushin, 2016). Among the four *CMT* subfamily members, *SmCMT1* and *SmCMT3* exhibited similar transcript abundance patterns, showing more stem-specific pattern, whereas no significant difference was observed for the transcript levels of *SmCMT2a* and *SmCMT2b* in the organs analyzed (Fig. 5). The transcript abundance patterns of *SmCMTs* are consistent with their phylogenetic relationships (Fig. 4). The transcript level of *SmDRM1* was significantly higher in flower and stem than root and leaf (Fig. 5). *SmDRM2* was higher in stem, followed by flower, and less in root and leaf (Fig. 5). The transcript abundance patterns of *SmDRMs* are consistent with the role of *DRM* subfamily members in *de novo* methylation (Cao & Jacobsen, 2002a; Cao & Jacobsen, 2002b; Law & Jacobsen, 2010). *SmDNMT2* exhibited similar transcript levels in all tissues analyzed, suggesting its importance in plant development.

**Figure 6:**
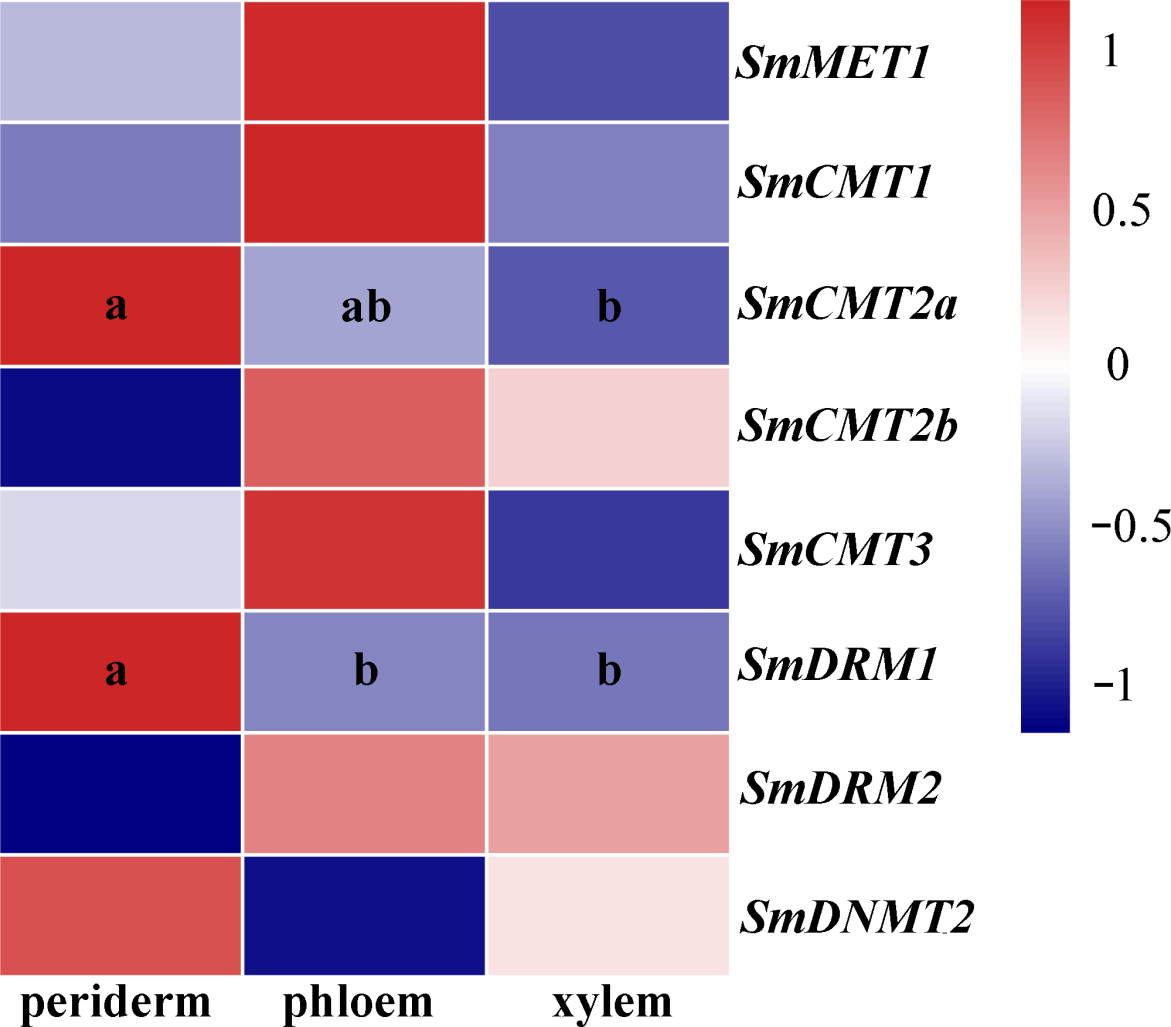
Transcript levels of *SmC5-MTases* in periderm, phloem and xylem of *S.miltiorrhiza* roots. Differential transcript abundance was analyzed using cufflinks software. *P* < 0.05 was considered statistically significant and was indicated by different letters. Absence of letters indicates that no significant different was observed among three tissues.

*S. miltiorrhiza* roots are major medicinal materials of various TCMs. To further investigate possible roles of *SmC5-MTases* in *S. miltiorrhiza*, RNA-seq data from periderm, phloem and xylem of roots were analyzed. Differential transcript abundance was observed for *SmCMT2a* and *SmDRM1* (Fig. 6, Table S3), indicating the role of *SmCMT2a* and *SmDRM1* in the biosynthesis of bioactive compounds.

### Responses of *SmC5-MTases* to yeast extract and methyl jasmonate treatments

**Figure 7:**
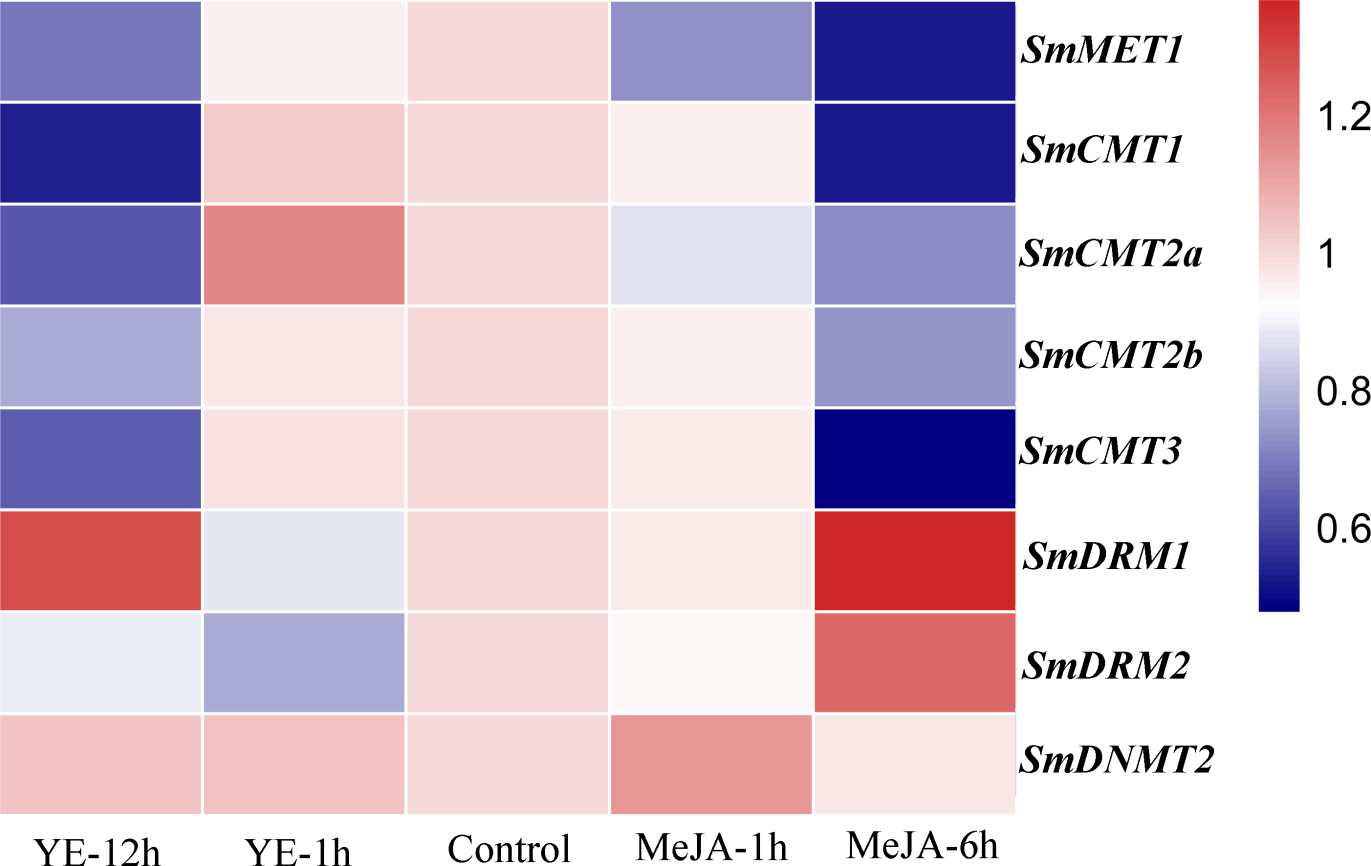
Responses of *SmC5-MTase* genes to YE and MeJA treatments in *S. miltiorrhiza* hair roots. Transcript levels were analyzed at 0, 1 and 12 h of YE treatment and at 0, 1 and 6 h of MeJA treatment. Differential analysis was performed using cufflinks software. *P* < 0.05 was considered statistically significant.

Yeast extract (YE) and methyl jasmonate (MeJA) are effective elicitors for tanshinone and phenolic acid production in *S. miltiorrhiza* (Chen et al., 2010; Kai et al., 2012; Zhou et al., 2017). The responses of *SmC5-MTases* to YE and MeJA treatments were analyzed using RNA-seq data from *S. miltiorrhiza* hairy roots (Zhou et al., 2017). The transcripts of all *SmC5-MTase* genes were detected in hairy roots (Fig. 7, Table S3). Compared with non-treated control, five *SmC5-MTases*, including *SmMET1*, *SmCMT1*, *SmCMT2a*, *SmCMT2b* and *SmCMT3*, were slightly down-regulated at the time-point of 12 h of YE treatment and at the time-point of 6 h of MeJA treatment. *SmDRM1* and *SmDRM2* were slightly down-regulated at the time-point of 1 h after YE and MeJA treatments, whereas they were up-regulated with treatment time extension. *SmDNMT2* was slightly up-regulated at the time-point of 1 h after two treatments, whereas it was down-regulated after 6 h MeJA treatment. Although all *SmC5-MTases* exhibited differential transcript abundance compared with control, the variance was not significant. It could be due to the short treatment time. Further analysis of *SmC5-MTase* genes in response to treatment with extended treatment time will help to elucidate the role of *SmC5-MTases* in secondary metabolite biosynthesis.

### Salicylic acid-responsive *SmC5-MTases*

**Figure 8:**
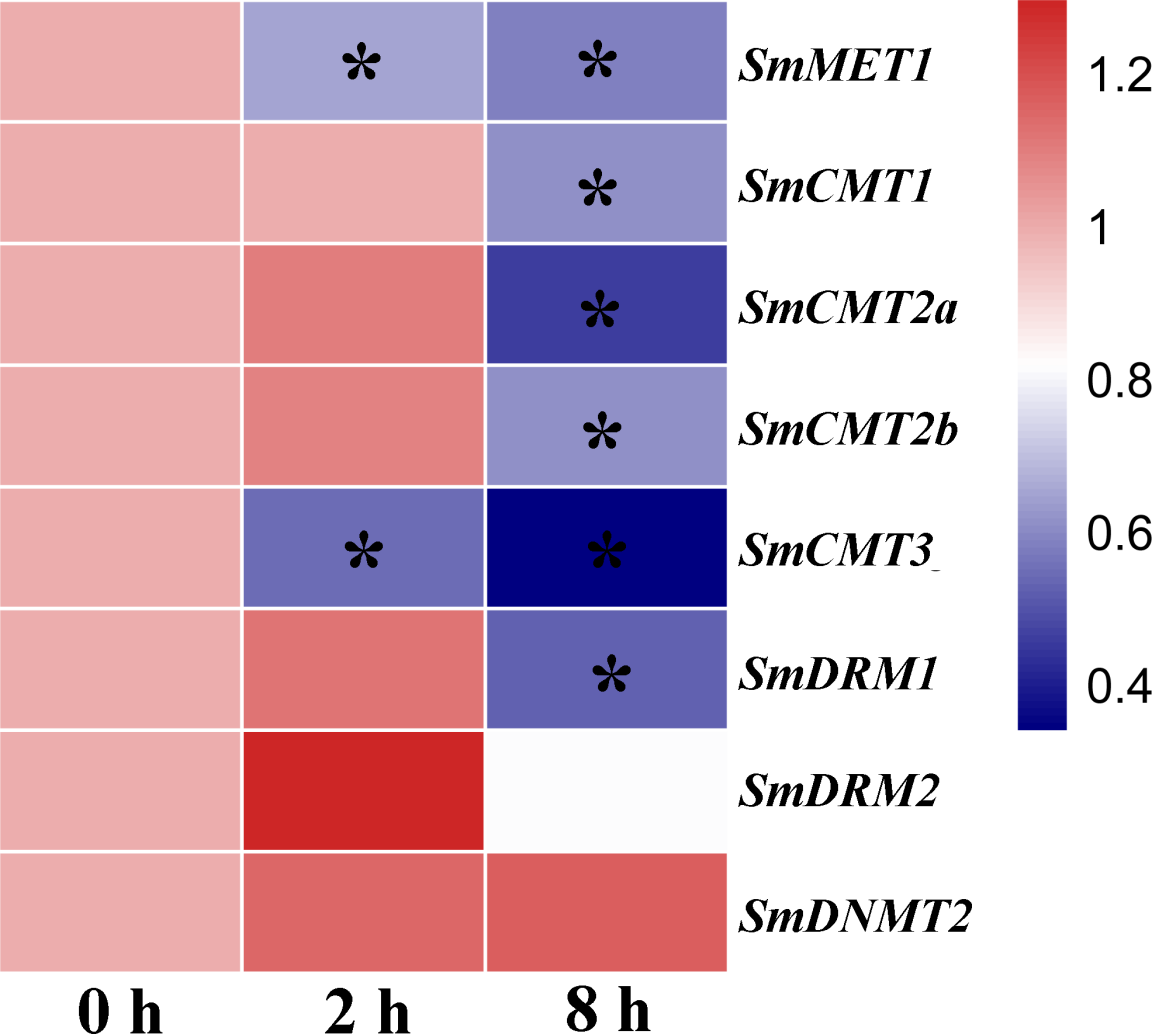
Responses of SmC5-MTase genes to SA treatment in *S. miltiorrhiza* cell cultures. Transcript levels were detected at 0, 2 and 8 h of SA treatment. Differential analysis was performed using cufflinks software. *P* < 0.05 was considered statistically significant. ^∗^presents significant differential transcript abundance compared with 0 h.

DNA methylation plays a vital role in plant defense by regulating the expression of a subset of stress responsive genes and is involved in priming of salicylic acid (SA)-dependent immunity (López Sánchez et al., 2016; Yu et al., 2013). In order to investigate the potential role of *SmC5-MTases* in plant defense, RNA-seq data from SA-treated *S. miltiorrhiza* cell cultures was analyzed (Zhang et al., 2016). The transcripts of all *SmC5-MTase* genes were determined in *S. miltiorrhiza* cell cultures and the majority of them were significantly suppressed at 8 h after SA treatment (Fig. 8, Table S3), suggesting the significance of *SmC5-MTases* in SA-dependent defenses.

## Discussion

Lipophilic tanshinones and hydrophilic phenolic acids are two main bioactive compounds from the roots/rhizome of *S. miltiorrhiza*. Understanding the regulatory mechanism of their biosynthesis and metabolism is important for *S. miltiorrhiza* quality improvement. Most of the studies about *S. miltiorrhiza* gene function concentrate on key enzyme genes and transcription factors associated with secondary metabolism (Li et al., 2015; Ma et al., 2012; Xu et al., 2015). The significance of DNA methylation has not been revealed in *S. miltiorrhiza*. C5-MTases are core elements of DNA methylation and play important roles in various biological processes (Candaele et al., 2014; Xing et al., 2015). As the first step to elucidate DNA methylation regulation in *S. miltiorrhiza*, we carried out genome-wide identification of *SmC5-MTase* genes. A total of eight such genes were identified and characterized. Integrative analysis of gene structures, sequence features, conserved domains, conserved motifs and phylogenetic relationships showed that *SmC5-MTase* genes could be divided into four subfamilies, including *MET*, *CMT*, *DRM* and *DNMT2* (Cao et al., 2014; Qian et al., 2014). *Arabidopsis* has four *MET* genes, whereas *S. miltiorrhiza* contains only one (Fig. 4), suggesting the loss of *MET* genes during *S. miltiorrhiza* evolution. Although all types of *CMTs*, including *CMT1*, *CMT2* and *CMT3*, exit in *S. miltiorrhiza* genome, *CMT2* possesses two paralogs, *SmCMT2a* and *SmCMT2b*. It indicates the occurrence of *CMT2* gene duplication. Gene loss and duplication imply functional redundancy and divergence of *SmC5-MTase* genes.

Although the number of each *S. miltiorrhiza SmC5-MTase* gene subfamily is different from that of *Arabidopsis*, the conserved domains are similar (Figs. S1–S5). Gene structures and sequence features also show the conservation of *S. miltiorrhiza* and *Arabidopsis C5-MTase* genes belonging to a subfamily, although intron size of *SmC5-MTase* genes exhibits apparent enlargement compared with their *Arabidopsis* counterparts (Table 1, Fig. 2). The results are confirmed by phylogenetic analysis of C5-MTase full-length protein sequences from sixteen plants (Fig. 4). Based on the phylogenetic tree, each C5-MTase subfamilies can be further divided into dicot and monocot groups. It indicates the origin of *C5-MTase* genes before dicot and monocot divergence.

DNA methyltransferases are responsible for establishment and maintenance of DNA methylation (Niederhuth & Schmitz, 2014). The transcript abundance of *SmC5-MTases* exhibited specific spatiotemporal patterns in *S. miltiorrhiza* (Fig. 5). It may result in variance of genome-wide cytosine DNA methylation in different *S. miltiorrhiza* tissues. During the development of *Arabidopsis* flowers, a number of cytosine sites were methylated *de novo* (Yang et al., 2015). *De novo* methylation could be caused by DRMs through the RdDM process (Cao & Jacobsen, 2002a; Cao & Jacobsen, 2002b). Significantly high transcript levels of *SmDRMs* in flowers and stems may result in *de novo* methylation of cytosine sites during *S. miltiorrhiza* flower and stem development. Sequence alignment (Fig. S4) and phylogenetic analysis (Fig. 4) showed that SmDRM1 and SmDRM2 were counterparts of AtDRM2 and AtDRM3, respectively. AtDRM2 has been showed to require catalytically mutated AtDRM3 for normal RdDM process in *Arabidopsis* (Henderson et al., 2010). It shows that SmDRM1 probably also requires assistance of SmDRM2 to accomplish RdDM process in *S. miltiorrhiza*. CMTs are plant-specific DNA methylation enzymes with the CHR domain. All of the CMTs identified and functionally characterized possess the CHR domain (Bewick et al., 2017). However, SmCMT2a lacks the CHR domain compared with other SmCMTs (Figs. S2–S3) in *S. miltiorrhiza*. Since other conserved domains exist (Fig. S3), SmCMT2a could play a role differing from the canonical CMTs. It is also possible that SmCMT2a has lost its biochemical activity. The function of SmCMT2a remains to be elucidated. Although DNMT2 contains highly conserved C-terminal methyltransferase domain and is able to interact with type-2 histone deacetylases (AtHD2s) in *Arabidopsis* (Song et al., 2010), its function remains largely unclear (Goll et al., 2006; Ponger & Li, 2005; Vieira et al., 2017). In this study, we found that *SmDNMT2* showed no differential transcript abundance in all tissues analyzed and various treatments (Figs. 5– 8). It is different from other *SmC5-MTase* genes, implying functional specificity of *SmDNMT2*.

Recent studies suggest that DNA methylation is involved in regulation of secondary metabolism (Bharti et al., 2015; Conde et al., 2017). Consistently, *SmCMT2a* and *SmDRM1* exhibited significant differential transcript abundance in periderm, phloem and xylem (Fig.  6, Table S3). Although the transcript abundance difference is not statistically significant, difference was observed for all *SmC5-MTase* genes in response to YE and MeJA treatment (Fig. 7, Table S3). Dynamic changes of *SmC5-MTases* could regulate the expression of functional genes, including many secondary metabolism-related genes. In addition, the majority of *SmC5-MTase* genes were significantly down-regulated by SA (Fig.  8). Analysis of DNA methylation mutant showed that DNA methylation could enhance resistance to the biotrophic pathogen *Hyaloperonospora arabidopsidis* (Hpa) through regulating SA-dependent defense genes in *trans* or *cis* (López Sánchez et al., 2016). Similar mechanism could also exist in *S. miltiorrhiza*.

## Conclusions

Eight *SmC5-MTase* genes were identified using whole genome sequence and RNA-seq data from *S. miltiorrhiza*. Based on phylogenetic tree and conserved domain distribution, *SmC5-MTase* genes were divided into four subfamilies, including *SmMET*, *SmCMT*, *SmDRM* and *SmDNMT2*. Comparative analysis of *SmC5-MTases* and *AtC5-MTases* revealed the conservation and divergence of plant *C5-MTases*. The transcript abundance analysis of *SmC5-MTase* genes suggests functional importance of *SmC5-MTases* in secondary metabolism and stress response in *S. miltiorrhiza.* The results provide useful information for understanding the role of DNA methylation in medicinal plant development and bioactive compound biosynthesis.

##  Supplemental Information

10.7717/peerj.4461/supp-1 Table S1 Primers used for qRT-PCR analysis of *SmC5-MTase* genes

10.7717/peerj.4461/supp-2 Table S2 *C5-MTase* genes identified in other species

10.7717/peerj.4461/supp-3 Table S3 The expression (FPKM) patterns of *SmC5-MTases* in various tissues and treatments of *S.miltiorrhiza*

10.7717/peerj.4461/supp-4 Figure S1 Sequence alignment of MET1 protein sequences from *S. miltiorrhiza* and *Arabidopsis*

10.7717/peerj.4461/supp-5 Figure S2 Sequence alignment of CMT1 and CMT3 protein sequences from *S. miltiorrhiza* and *Arabidopsis*

10.7717/peerj.4461/supp-6 Figure S3 Sequence alignment of CMT2 protein sequences from *S. miltiorrhiza* and *Arabidopsis*

10.7717/peerj.4461/supp-7 Figure S4 Sequence alignment of DRM protein sequences from *S. miltiorrhiza* and *Arabidopsis*

10.7717/peerj.4461/supp-8 Figure S5 Sequence alignment of DNMT2 protein sequences from *S. miltiorrhiza* and *Arabidopsis*

10.7717/peerj.4461/supp-9 Data S1 Obtained genomic sequences , ORFs, and deduced protein sequences Raw data.

## Additional Information and Declarations

### Data Availability

The following information was supplied regarding data availability: GenBank: accession numbers MG602207–MG602214.
