# Supplementary figures and images for: Identification and characterization of the cytosine-5 DNA methyltransferase gene family in Salvia miltiorrhiza
Source: PeerJ. 2018 Mar 5;6:e4461. doi: 10.7717/peerj.4461 (PMC5842782; doi:10.7717/peerj.4461)

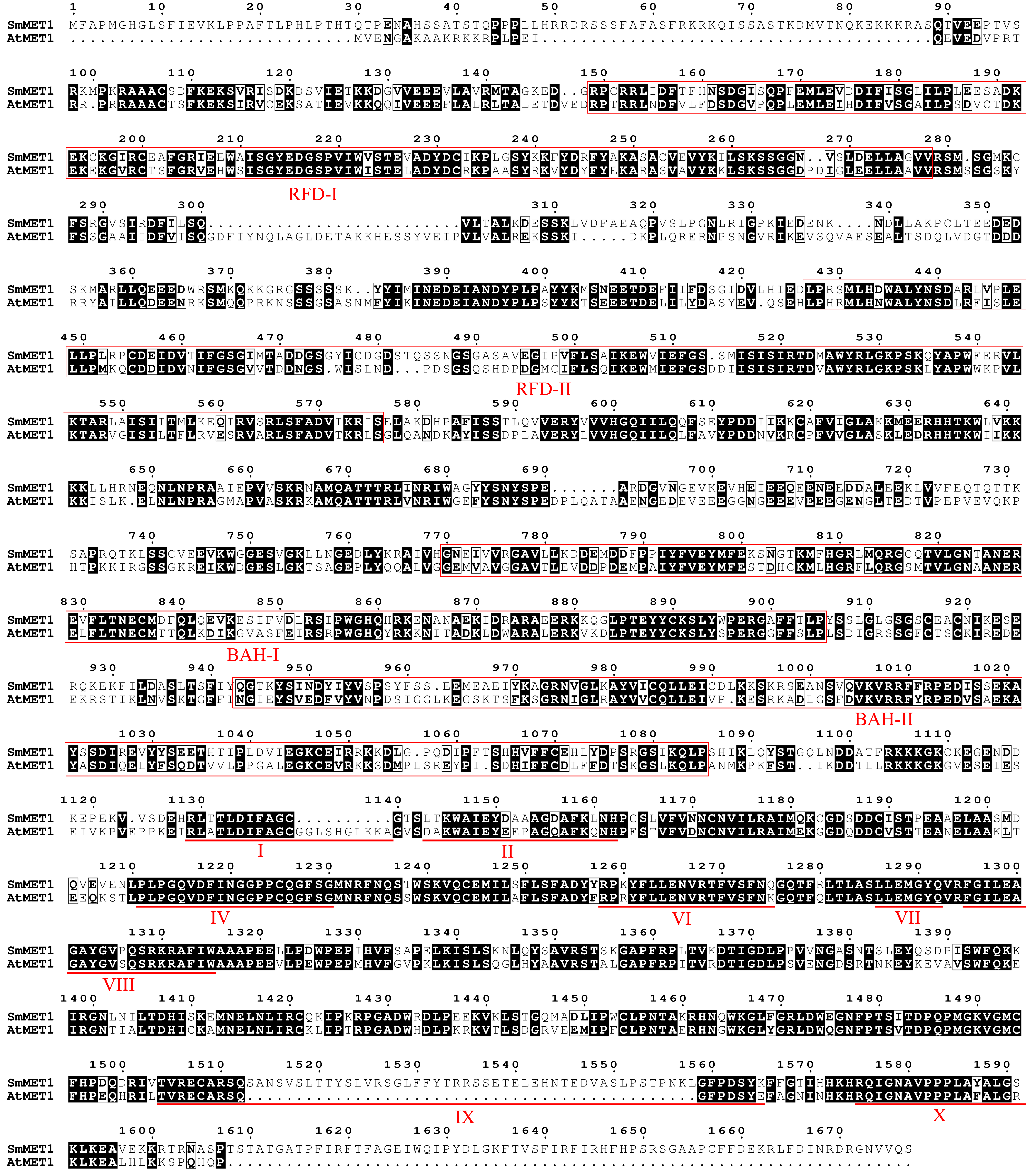

Supplement: Figure S1 [file peerj-06-4461-s004.png]

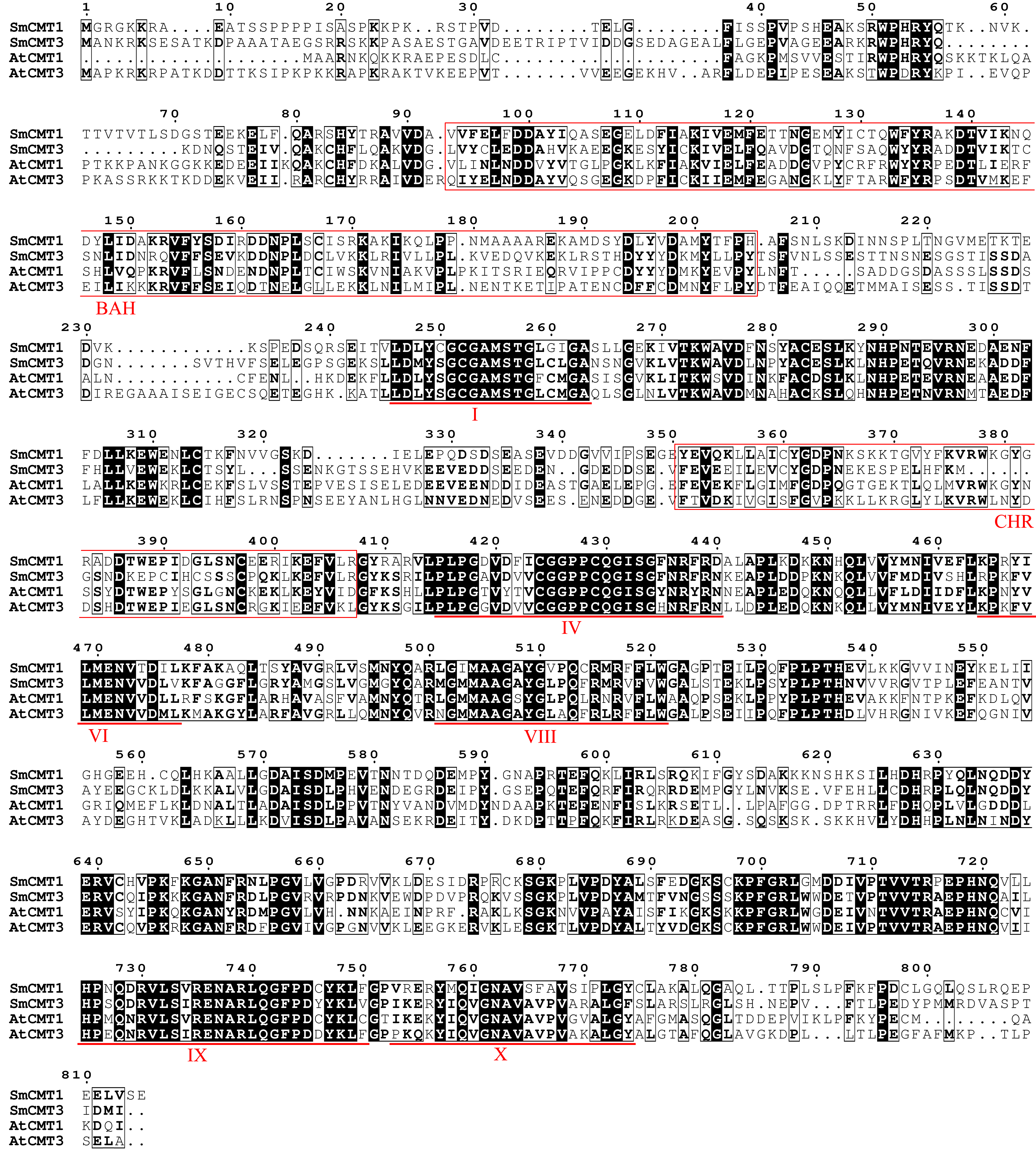

Supplement: Figure S2 [file peerj-06-4461-s005.png]

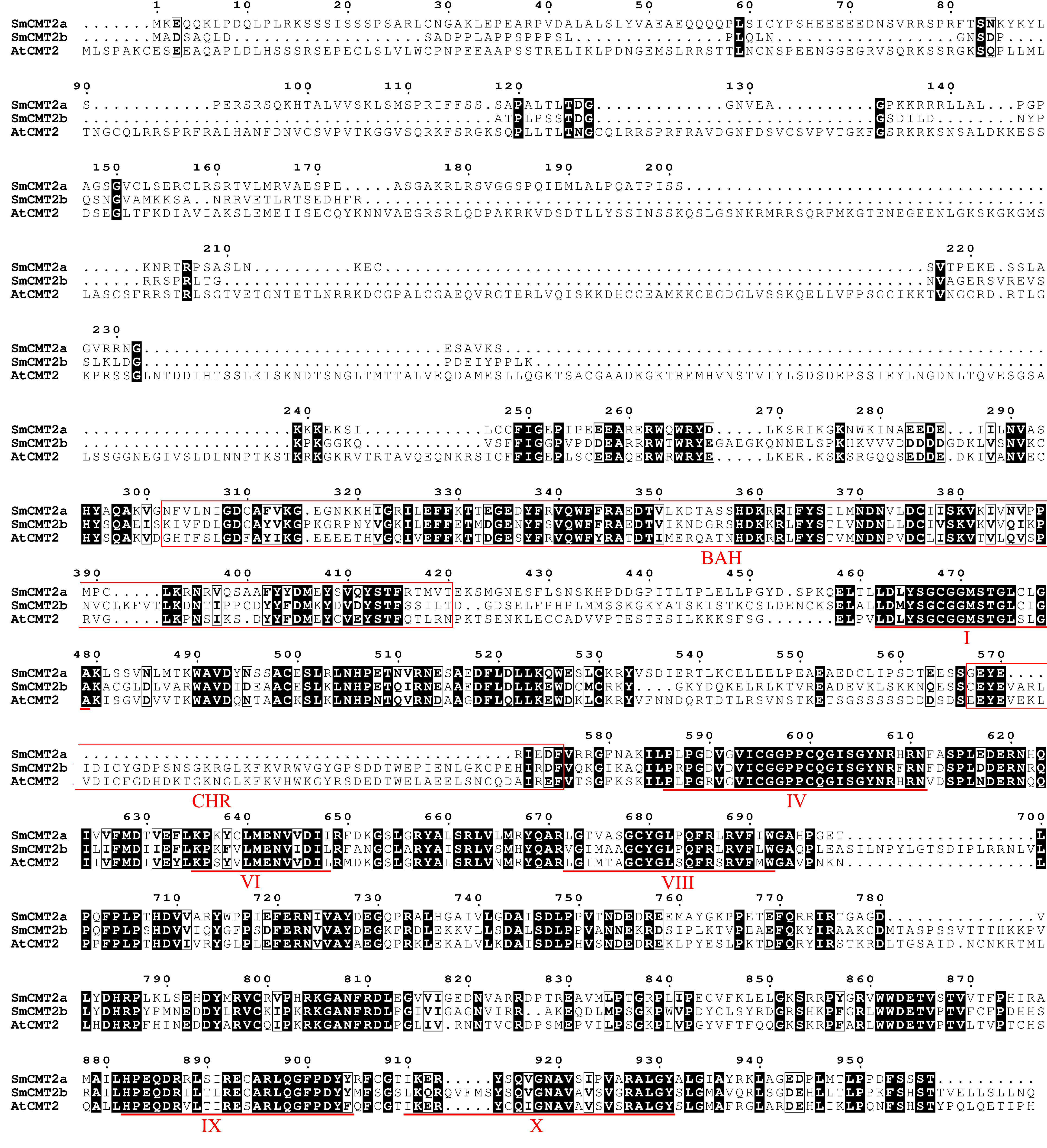

Supplement: Figure S3 [file peerj-06-4461-s006.png]

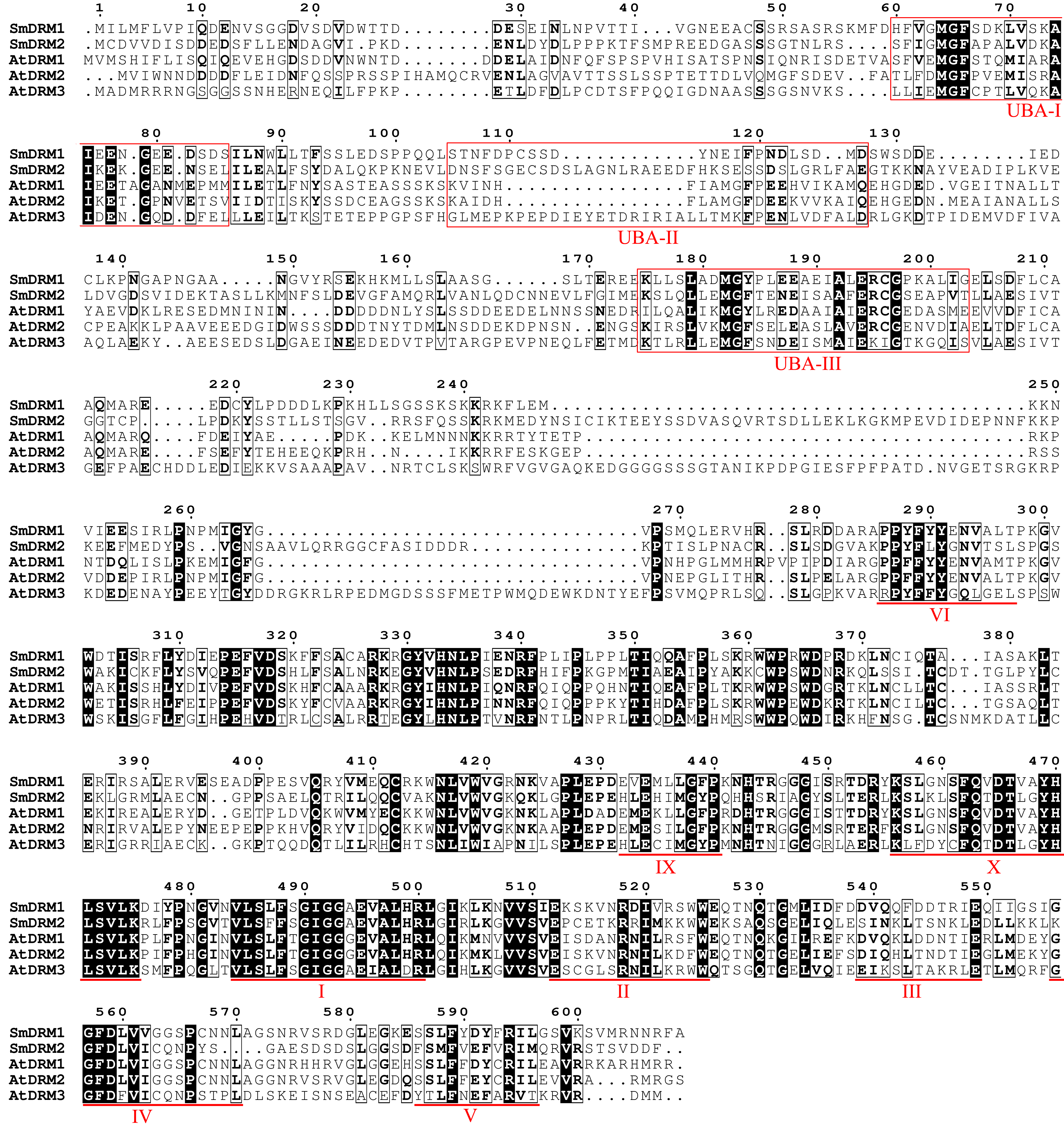

Supplement: Figure S4 [file peerj-06-4461-s007.png]

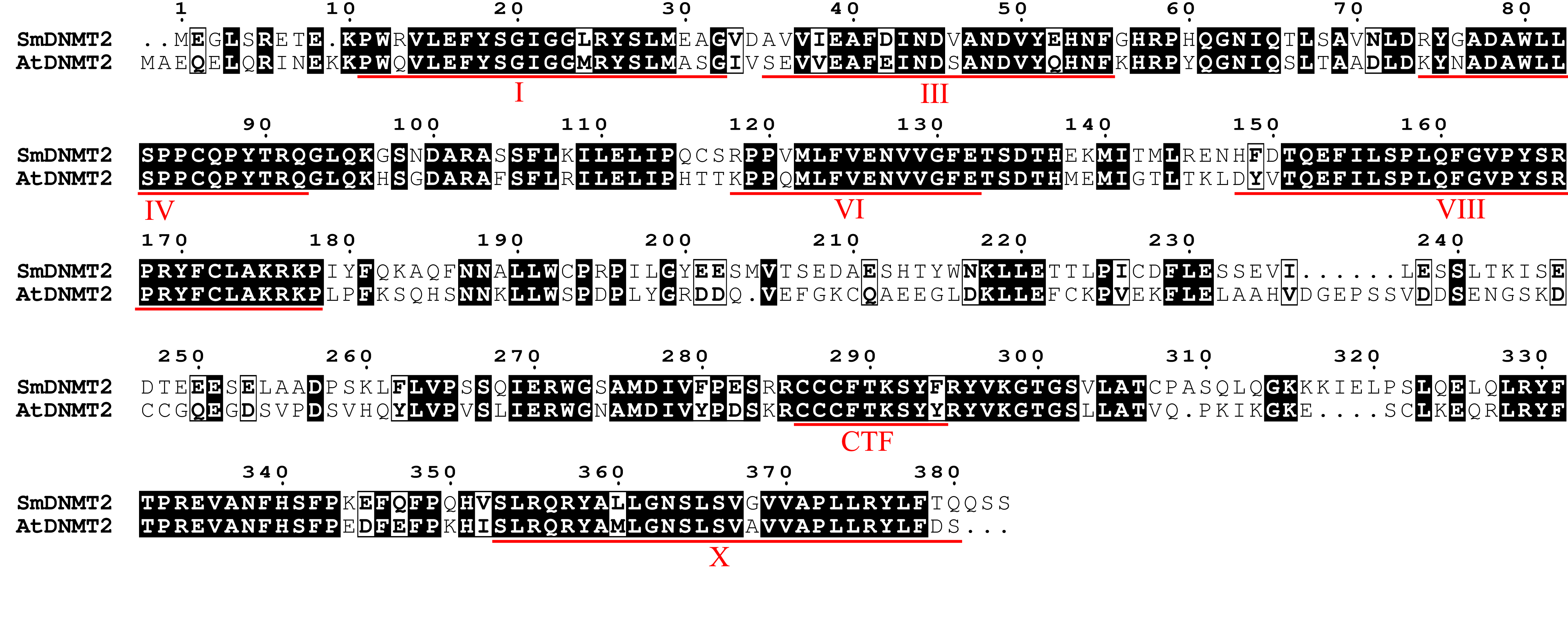

Supplement: Figure S5 [file peerj-06-4461-s008.png]
