# Supplementary material for: Identification and characterization of the cytosine-5 DNA methyltransferase gene family in Salvia miltiorrhiza
Source: PeerJ. 2018 Mar 5;6:e4461. doi: 10.7717/peerj.4461 (PMC5842782; doi:10.7717/peerj.4461)
Supplement: Data S1 — Raw data. [file peerj-06-4461-s009.docx]

>SmMET1 genomic sequence

1 ATGTTTGCTC CCATGGGTCA TGGCTTATCT TTCATTGAAG TATGACTTTC CATTTCGATT

61 ACACGATTTA ACTTGTTAAG GTAGCATCTT TTTAAACTAA AATAAAATTT TAACAATAAT

121 ACAAAAGAAG AAACATTTGA GTTTTCAAAT TTCACCTAAG GAAAAAGAAG GCCGCGTTTG

181 ATCTGGTGAT GGTATTGGCG CGACAGAACA AAAAATTGAA ATGTTAGCGC CATTTGTCTC

241 CCTTCCTTCC CCCTAAATTT TCAGGCTTTT ATTTAGTGGT TGGGCTAGGG TTCCGATCTC

301 TCCCTCTCTT TCTCTCTCTC ATCAATCTCC GCTGCCTGCT CTCTTCCTTG TTCCGGCATA

361 TAGGGCAGCC ACCGGGACTT GCTCTCACTC TCAGTCGCCC ATTCTTGATT TTTCATCCTT

421 TTCATTCTTG ATTTTCATCA TCGCCTTCAA CAGGTCAAGC TACCACCCGC CTTCACCCTC

481 CCTCATCTCC CGACGCACAC ACAGACACCT GAAAACGCAC ACAGCTCGGC CACCTCTACT

541 CAACCACCGC CGCTGCTCCA TCGCCGGGAC CGGTCATCCT CTTTTGCCTT CGCCAGTTTC

601 CGGTAAGTTT AAGTTTTTCC TTTGTTTTTT TTTTTTTTTT TGTAGGAAAT TGAATCAATT

661 TTGTTGTTAA ATCTTACCAT CAAAATGAAC ATATTCTGAG CACTTAAGTT TTTCCTTTTT

721 TTTTTTATAA GTCTTTTCAG CGTTGTTTTA TTTTGGTAAA GACTAATTTA TCTGAATTGT

781 AATTCTTTTC TTTTCTTTGT GACATTGAAT TGCAACCTTG ATGCTTCATT TCATTTCCGG

841 AAAGATTTGA GAATAATAAT TGTAGCTGTT GTCTATGTTT TCTTGTCAGT TTCCAATTGA

901 TTTGCTTGCA TGGAGAATTT GATGCTATAT ATGTATATAT TTTGCTTTGT CATTGATCTA

961 GAAATGATTT GGAAAATAAT GTACGAATCA GCGCAATTAA GCTCGGATAT AATGAGTTGC

1021 TATCAACTTA CGCTCAGCCT GGCACTGTTT GGGATGACTC TGCTCACTAA CACAGATATT

1081 GGCTCTTCTT TGGTACATCA TAAGGCCACC CAGTGGCTAT GGATTCCTAG AGCAATTTTA

1141 ACAACATTAA GTCGTTTTGT CTGGAGGAAC ACAATGATAT ATTGTAACGG AATTTTGAGT

1201 TTTTTTGGTG GCTAGCATAT ACATTTTTCA AGTAAATAAT TACTAAAACC ATTCACCCAG

1261 ATTTATGCCG AGTCTGGATT TTTGCTCACT GATTGATGGA TGGCCATAAT CTCCATTTCT

1321 GTTATTCAAC ATCTTTCTTT AAATGAAACT AATAACATTT ATTTTCTATG AAATGAAATT

1381 TACCTCTAAC TTGAAACATT TTCCATTTGC ACTTTTAATG TATTCTGTTC AATCTTTAAC

1441 TAGGACTTTT ATGTGCCTTT AATTCACTGA TTGCCTAGCT CTATGCACTC CTTCATTTAT

1501 GGGATATAAT ATATTGTAGT TTTTCAATAA CATTAGTAGT TTGTTCCTGG AATTTCTCTT

1561 GGTTTTCCTA GGGATCAGAA AGAGGAAACA GATCAGTTCT GCATCTACGA AAGATATGGT

1621 GACTAATCAA AAAGAAAAAA AGAAGCGTGC TTCTCAAACT GTTGAAGAGC CCACTGTTTC

1681 TCGTAAAATG CCAAAGAGAG CTGCTGCTTG TTCAGATTTC AAGGAGAAAT CTGTTCGAAT

1741 ATCCGATAAA GATTCCGTAA TTGAAACAAA AAAAGATGGA GTTGTTGAGG AAGAAGTCCT

1801 AGCTGTTCGT ATGACTGCTG GAAAGGAGGA TGGTCGCCCA TGTAGACGGC TCATAGATTT

1861 CACATTTCAT AACTCAGATG GCATCTCGCA GCCCTTTGAA ATGCTGGAAG TGGATGATAT

1921 CTTCATCTCT GGCCTTATCT TGCCACTCGA GGAAAGTGCT GACAAGGAGA AATGTAAAGG

1981 AATAAGATGC GAAGCATTTG GGCGCATTGA AGAATGGGCT ATATCTGGTT ATGAAGATGG

2041 ATCGCCTGTA ATATGGGTTT CCACAGAGGT TGCCGATTAT GATTGCATTA AGCCCTTGGG

2101 AAGCTATAAG AAGTTCTATG ACCGTTTTTA TGCTAAAGCT AGTGCTTGTG TTGAGGTCTA

2161 TAAGATATTA TCAAAATCTT CTGGAGGCAA TGTAAGCCTT GATGAGTTGC TTGCTGGGGT

2221 CGTGCGTTCC ATGAGTGGGA TGAAGTGTTT TTCCCGTGGT GTATCTATCA GGGATTTTAT

2281 TCTTTCTCAG GGTGAATTCA TTTATAACCA ACTTATTGGC TTGGATGAGA CTTAAAAAAA

2341 GAGTGAAAAC CTTTTTGTTG GGCTACAAGT CCTAACTGCT CTTAAAGATG AAAGTAGTAA

2401 GTTGGTAGAC TTTGCAGAAG CTCAACCTGT CTCTTTGCCA GGGAATTTGA GAATTGGCCC

2461 CAAAATTGAG GATGAAAACA AAAATGATTT ACTGGCTAAA CCTTGTCTGA CAGAAGAAGA

2521 TGAAGATTCA AAAATGGCAA GGCTGTTACA GGAAGAAGAG GACTGGCGCT CAATGAAGCA

2581 GAAGAAGGGC CGGGGTTCCT CTTCATCGAG CAAATATTAC ATTATGATCA ATGAGGATGA

2641 AATAGCAAAT GACTATCCTC TGCCTGCCTA TTACAAGATG TCAAATGAAG AAACTGATGA

2701 GTTTATTATT TTTGACAGTG GCATAGACGT GCTACATATT GAAGATCTGC CCCGAAGCAT

2761 GCTCCACGAC TGGGCTTTGT ACAATTCAGA TGCTAGGCTG GTTCCGTTAG AGCTCCTGCC

2821 CCTGAGACCC TGTGATGAAA TTGATGTTAC TATATTTGGG TCAGGCATAA TGACAGCAGA

2881 TGATGGATCT GGATACATTT GTGATGGTGA TTCTACTCAG TCATCAAATG GTTCTGGGGC

2941 TTCTGCAGTT GAAGGCATCC CTGTTTTCTT GAGTGCAATA AAAGAGTGGG TGATTGAGTT

3001 TGGGTCCTCG ATGATTTCCA TTTCAATACG AACGGATATG GCCTGGTATG TTACTCCCTC

3061 TGTGTCTCAT TTGAGAATAT ATGTTAATGC AGTATATTCT GTTTAAGTGC GCAGTTTTAA

3121 TTGTTCTATA TTTATTGATT GGTTGGCAAT ATGAATTTTA TCGTTATGAG TTGGATCATC

3181 TTGCTTGTTC GATCTGTCCT AAATGATATT GCATTAAATA TTGAAGTACT AATTGTGGTA

3241 GTATAGGATA CTAAAGCCTA AATTTAACAG CATAGATGAA ATACTAAAGA AAGTACAGGC

3301 TGTAATGTTG CATTTACGGA ACTTCACATC TTAGATTTGT CCCAGTTAAT CAATAATGTC

3361 TAATGATTTC CTCGTTTTAC ATGATTTGGA GGTTTTGAAG TTTCTTGTGC ATAAATGCAT

3421 TGTTTCTTTC TCTTCTTCAG GTACAGGCTT GGAAAGCCTT CAAAACAATA CGCTCCTTGG

3481 TTTGAGCGAG TCTTGAAAAC TGCGAGGCTT GCCATCAGTA TCATTACCAT GTTGAAGGAG

3541 CAGATCCGGG TGTCAAGACT TTCTTTTGCT GATGTAATCA AGAGAATCTC AGAACTTGCC

3601 AAGGATCACC CTGCTTTTAT ATCATCTACT CTACAAGTAG TGGAGAGATA TGTGGTTGTG

3661 CATGGTCAGA TTATTCTGCA GCAATTTTCT GAATATCCTG ATGATATTAT AAAGAAGTGT

3721 GCTTTTGTAA TTGGCCTTGC CAAGAAAATG GAAGAGAGGC ACCATACCAA ATGGCTTGTC

3781 AAGAAAAAGA AGCTTCTGCA TAGGAATGAA CAGAACTTGA ATCCCAGGGC TGCTATTGAG

3841 CCTGTTGTGT CCAAAAGGAA CGCTATGCAG GCAACTACAA CAAGGCTAAT CAACAGAATC

3901 TGGGCAGGAT ACTATTCAAA TTACTCACCC GAGGCACGAG ATGGAGTGAA TGGTGAAGTA

3961 AAAGAGGTTC ATGAAATCGA AGAGCAAGAG GAAAACGAAG AGGATGATGC TTTGGAGGAG

4021 AAATTGGTTG TATTTGAACA AACTCAGACA ACTAAGTCGG CTCCAAGGCA AACCAAGTTA

4081 AGTTCATGTG TAGAGGAAGT AAAGTGGGGT GGGGAATCTG TAGGCAAACT ACTTAATGGT

4141 GAGGATTTAT ATAAGAGGGC CATAGTTCAT GGTAATGAAA TTGTGGTCAG GGGGGCTGTT

4201 CTTCTGAAAG ATGATGAAAT GGATGATTTT CCACCCATCT ATTTTGTAGA ATACATGTTT

4261 GAAAAGTCAA ATGGAACTAA AATGTTTCAC GGGAGATTGA TGCAACGGGG CTGCCAAACT

4321 GTGCTTGGAA ACACAGCTAA TGAAAGGGAG GTATTTTTGA CAAATGAGTG TATGGATTTC

4381 CAACTACAAG AAGTCAAAGA AAGTATATTT GTTGATCTAA GATCAATTCC TTGGGGTCAT

4441 CAGCACAGAA AAGAAAATGC TAATGCTGAG AAAATTGATA GAGCAAGAGC AGAGGAAAGA

4501 AAGAAGCAAG GATTGCCTAC TGAATATTAC TGCAAAAGTC TGTATTGGCC TGAGAGAGGT

4561 GCCTTCTTTA CTCTTCCATA TAGTTCGTTG GGGCTTGGAT CTGGCTCTTG TGAGGCTTGC

4621 AATATAAAAG AAAGTGAAAG GCAAAAGGAA AAGTTTATAT TGGATGCATC TTTGACCAGT

4681 TTTATATACC AGGGAACTAA ATATTCTATA AATGACTATA TCTATGTGAG TCCTTCATAC

4741 TTTTCATCAG AAGAAATGGA GGCTGAGATT TACAAGGCTG GGAGAAATGT CGGATTGAAG

4801 GCGTATGTAA TATGCCAGCT GCTTGAAATT TGTGATCTCA AGAAGTCCAA AAGGAGCGAA

4861 GCCAATTCTG TTCAGGTCAA AGTCAGGAGA TTTTTCAGGC CGGAGGATAT ATCATCAGAG

4921 AAGGCATACT CGTCGGATAT TCGAGAGGTG ATACTTCTTC TATCCATGTC AAAATATCAT

4981 TGAATATCTA GCGTTTTATT GGCATTTTGA GCGCTTGCAA TTTACAATGT AGAAATTTGT

5041 TTGTGTTTGT CATTAATGTT TATCAATATT ATTTCTGACC ACTTACGGTT TTCTGAATGA

5101 TTTTTCAGGT CTATTATAGT GAGGAGACAC ACACCATACC TCTAGATGTT ATTGAAGGAA

5161 AATGTGAGAT CAGAAGAAAG AAGGATCTTG GGCCTCAAGA TATCCCCTTT ACCTCTCACC

5221 ATGTTTTCTT CTGTGAACAT CTGTATGATC CTTCCAGAGG ATCCATTAAG CAGGTTTCCC

5281 CCTTCCCCTT TTCTTGAGGG CAGAACTGAA TTTGGCCATT TACATCTTAT TATGATAATA

5341 GTATCATTTT ATTTTCTAAT CAATTGTCTT TTGTTATTGT CCATGCACAG TTGCCATCAC

5401 ATATTAAATT ACAGTACTCA ACCGGGCAGC TGAATGATGA TGCCACATTT AGGAAAAAGA

5461 AAGGGAAATG CAAAGAAGGA GAGAATGATG ACAAAGAACC TGAGAAAGTA GTATCTGATG

5521 AGCACCGCTT GACTACTTTG GATATTTTTG CTGGCTGTGG TGGCTTGTCT GAAGGACTGG

5581 AGCAATCAGG CAGGCTTATA ACTGATATCC TACATTACAT TAAGGCTTAT AATTCCCAGT

5641 AAGTATAATC TGTTATTTTG GGTTTTAATG CCTGCAGGTA CCTCTCTCAC AAAATGGGCT

5701 ATTGAGTATG ATGCTGCTGC TGGAGATGCA TTTAAACTCA ATCATCCTGG GTCTTTGGTA

5761 TTTGTAAACA ACTGCAATGT TATCCTCAGG TAGATTTCTT GCATTGAATT TAAAATCCTT

5821 GTCGTGGAAA CTTTAGCAAC TTAGCACAAA CCAGCATGGA TATAGGAGAA CTAAAATCAT

5881 GTAAATGCAG GGCAATCATG CAAAAGTGTG GGGATTCAGA TGATTGCATT TCCACCCCAG

5941 AGGCTGCTGA ATTGGCTGCA TCAATGGACC AAGTGGAAGT TGAGAACCTT CCACTACCCG

6001 GACAAGTTGA TTTTATTAAT GGTGGGCCTC CTTGTCAGGT CAGTTAAACA ACACAATTTC

6061 AGATTTCCAG CGAATCATTA AGTTACATTG CTAAAAGTAT TCAGAAATCT CTTGTAGGGT

6121 TTTTCTGGAA TGAATAGGTT CAATCAAAGC ACATGGAGTA AAGTCCAATG CGAGATGATT

6181 TTGTCATTTT TATCCTTTGC TGATTACTAC CGTCCAAAGT ATTTCCTTCT CGAGAATGTT

6241 AGGACCTTTG TATCTTTCAA CCAAGGGCAG ACCTTTCGCT TAACTTTAGC TTCACTCCTT

6301 GAGATGGGAT ATCAGGTATG CACTGTTACT CATTTTTTCA GAATAGAATC TCTGGATCTA

6361 CATTTCCTAG TGATTTTATT TTTTTATTTT TTTTATTTAT ATTTTATGTT TATGGAAGGT

6421 GAGATTTGGT ATCCTTGAAG CTGGTGCATA TGGAGTTCCT CAATCTAGAA AGAGAGCTTT

6481 CATATGGGCA GCGGCTCCTG AAGAGCTGCT TCCAGATTGG CCAGAGCCAA TTCATGTCTT

6541 TTCAGCACCA GAGCTGAAAA TCTCATTGTC GAAAAACTTG CAATACTCTG CTGTCAGGAG

6601 TACTTCAAAA GGTGCTCCAT TCCGTCCTCT TACAGTCAAA GATACAATTG GAGATCTTCC

6661 ACCAGTGGTC AACGGTGCAT CTAACACAAG CTTAGAGGTG AATTTTGAGT GCCATCCAGA

6721 AATAAAAAAA TTTATTGCTC TTACATCAAT GGTCCATACA ATTTAACACT AGGCAATGCT

6781 AGGCCACTTT CTCACTACTA GTTATATCAT GTTCTGTTGT CTATTAATGC AGTATCAAAG

6841 CGATCCAATT TCGTGGTTCC AGAAGAAAAT CCGTGGTAAC TTGAACATAT TAACAGACCA

6901 CATATCAAAA GAAATGAATG AGCTTAATCT GATCAGGTGT CAGAAAATAC CCAAGCGCCC

6961 TGGTGCTGAT TGGAGAGATC TGCCCGAGGA GAAGGTGTCA ACAAAACCTT ATTGGTCTTA

7021 GTTTTGCTTC TGAATCTTGT ATTTTCTGTG AACATTGGAT CAAATGTCTA ACTTGTCTTC

7081 TAAAAAATTT ACAGGTCAAG TTATCTACTG GCCAAATGGC TGATCTGATA CCATGGTGCT

7141 TGCCCAATAC GGCCAAGAGG CACAATCAGT GGAAGGGACT TTTTGGCAGG TTGGACTGGG

7201 AGGGGAATTT TCCAACTTCA ATAACTGACC CTCAACCAAT GGGTAAAGTT GGAATGTGCT

7261 TTCATCCTGA TCAAGACAGG ATTGTGACAG TCCGTGAATG TGCACGTTCT CAAGTAAGCA

7321 TGCTCCAACT TTTCAACAAA AGAAAGTTTG CATCATGGAG TTTGCCAAAC GATAACTGGT

7381 TTTTATTGGG TCGTCTATTT CTTTAGTCTG CAAATTCTGT TTCCCTGACC ACATATTCCC

7441 TTGTACGTTC TGGCTTGTTC TTTTACACCA GAAGATCTTC AGAAACTGAG CTAGAACACA

7501 ATACAGAAGA TGTGGCTTCA CTTCCTTCTA CTCCTAATAA ACTTGTGCAT ATAAAACCTA

7561 ATATCTTCAT TGCACTAGAC ACTGATTCTG CAGCATTATA GAAGTGTAGA GAAATAAGTA

7621 TGGGCTGGAT TTCTTCATTC TATACTCGAC TTGTCTATTC TAATCAGTCT ACTTGTTTGT

7681 GCAGGGTTTC CCGGACAGCT ATAAATTTTT TGGTACTATT CACCACAAGC ACAGACAGAT

7741 CGGAAATGCT GTTCCTCCTC CTCTGGCTTA TGCCCTTGGA AGCAAACTGA AGGAAGCTGT

7801 CGAGAAGAAA AGAAGTTAGA AGTCTGTAGA TGACAATCAT GCCAATGGTC GTCGTTAAAG

7861 AACAAAATTG CAATGTGCTG ATGGTTTGTC TCGTCCATTC CTTGGAATGT TGATAATGTA

7921 GATGTGATTC CTTTAGTAAA CAATCAATTT GAGCAAAGTT GTATAGATCG GCTCAACATT

7981 ATTAGTTGGA AATTTCGGAT TGAGGGTGAC TCGCCTTTGA AGATTTGAGT ATGTATGTAC

8041 CAGTTACTCT TAGTTGGGGG ATCTACCCAA AATATATCGA ATTTGAGGGG ATTTTTTTGG

8101 ATTAATTATA CTCTATAGTT TGAATTTTAA TTTTGTAGGT TATTTTATGG TCTGAATTTA

8161 CAAAGTTACA AAAATGGTTT GAACTTTGCC TCGGTGTTCA CTTTATAGTT GGGGAAAAAG

8221 AATTTGACTA CGGAAAAATG ATGTGGACTG TTCGAGTTTG ATGTGAATTT TTGCCTAGTA

8281 GAAATGAAAT TAGATTAGAT GGAAATTATA TTGTGTCAAC TATACTTTAT GGCTCAAAGT

8341 TTGGTTTGTT GATAACTTTA TGACCTAATT TTATGAATTT GAAAAATATT TTTGAAATAT

8401 TTCCGGCTAT GAAAGAATTT GAAAAGACGA CTCTAATTGT ATGCATATCA CTAAATACAA

8461 TTTCTAAAAT ACCTGTATAT AGTTGAAAGT TTTGCTAATT ACGCAAATAC ATAAATGCTG

8521 GATAGAATGA TAGAACAATC AATCCAGCCT ATACTTATTT CACAAAATAC CTCTCAGTGT

8581 AGAATCATTA TTGTAAACAT ACATTCAATA CTTAAGTAGA AATTCTGTCC AAATCAATAT

8641 AATAAAGGGT TAATAGTCGA AAAATACACG AACTTTTATG AAATTTACAA ATTGCACGCG

8701 AGCTTCAAAA ATAGCCCAGA ATACATCACT TTTAATTTTG TCGTAAATTG CACACGCGAA

8761 AGTTCCGGTA GCTCTTAAGT TTGACCGGCG ATGACGTGGA CAATATTTAA CATTACATGG

8821 CATTACTCGT GGCTTTTTTT TACACGTGGG CAAAACGACG TCGTTTTGAT ATTTTGAAGA

8881 ATTTAGTAAA AAAAAAATTT GCCCCCCTAC CGCCTCCCTC TTCCACTCAA CCGCCACCTC

8941 CACCCTTCTA GATCTGGAGC GCCCCCTGCT TCTCCAGCGA GGAACGCCTC TCCGACATCA

9001 ACCGCGACAG GGGCAACGCC GTTCATCCGT TTCACCTTTG CTGGAGAAAT CTGGCAAATT

9061 CCTTATGATT TGGGAAAATT TACAGTGTCG TTCATCCGCT TTATCCGCCA CTTCCATCCT

9121 TCCAGATCTG GAGCCGCCCC CTGCTTCTTC GATGAGAAAC GCCTCTTCGA CATCAACCGT

9181 GACAGGGGCA ACGTTGTTCA ATCATAA

>SmMET1 ORF sequence

1 ATGTTTGCTC CCATGGGTCA TGGCTTATCT TTCATTGAAG TCAAGCTACC ACCCGCCTTC

61 ACCCTCCCTC ATCTCCCGAC GCACACACAG ACACCTGAAA ACGCACACAG CTCGGCCACC

121 TCTACTCAAC CACCGCCGCT GCTCCATCGC CGGGACCGGT CATCCTCTTT TGCCTTCGCC

181 AGTTTCCGAA AGAGGAAACA GATCAGTTCT GCATCTACGA AAGATATGGT GACTAATCAA

241 AAAGAAAAAA AGAAGCGTGC TTCTCAAACT GTTGAAGAGC CCACTGTTTC TCGTAAAATG

301 CCAAAGAGAG CTGCTGCTTG TTCAGATTTC AAGGAGAAAT CTGTTCGAAT ATCCGATAAA

361 GATTCCGTAA TTGAAACAAA AAAAGATGGA GTTGTTGAGG AAGAAGTCCT AGCTGTTCGT

421 ATGACTGCTG GAAAGGAGGA TGGTCGCCCA TGTAGACGGC TCATAGATTT CACATTTCAT

481 AACTCAGATG GCATCTCGCA GCCCTTTGAA ATGCTGGAAG TGGATGATAT CTTCATCTCT

541 GGCCTTATCT TGCCACTCGA GGAAAGTGCT GACAAGGAGA AATGTAAAGG AATAAGATGC

601 GAAGCATTTG GGCGCATTGA AGAATGGGCT ATATCTGGTT ATGAAGATGG ATCGCCTGTA

661 ATATGGGTTT CCACAGAGGT TGCCGATTAT GATTGCATTA AGCCCTTGGG AAGCTATAAG

721 AAGTTCTATG ACCGTTTTTA TGCTAAAGCT AGTGCTTGTG TTGAGGTCTA TAAGATATTA

781 TCAAAATCTT CTGGAGGCAA TGTAAGCCTT GATGAGTTGC TTGCTGGGGT CGTGCGTTCC

841 ATGAGTGGGA TGAAGTGTTT TTCCCGTGGT GTATCTATCA GGGATTTTAT TCTTTCTCAG

901 GTCCTAACTG CTCTTAAAGA TGAAAGTAGT AAGTTGGTAG ACTTTGCAGA AGCTCAACCT

961 GTCTCTTTGC CAGGGAATTT GAGAATTGGC CCCAAAATTG AGGATGAAAA CAAAAATGAT

1021 TTACTGGCTA AACCTTGTCT GACAGAAGAA GATGAAGATT CAAAAATGGC AAGGCTGTTA

1081 CAGGAAGAAG AGGACTGGCG CTCAATGAAG CAGAAGAAGG GCCGGGGTTC CTCTTCATCG

1141 AGCAAATATT ACATTATGAT CAATGAGGAT GAAATAGCAA ATGACTATCC TCTGCCTGCC

1201 TATTACAAGA TGTCAAATGA AGAAACTGAT GAGTTTATTA TTTTTGACAG TGGCATAGAC

1261 GTGCTACATA TTGAAGATCT GCCCCGAAGC ATGCTCCACG ACTGGGCTTT GTACAATTCA

1321 GATGCTAGGC TGGTTCCGTT AGAGCTCCTG CCCCTGAGAC CCTGTGATGA AATTGATGTT

1381 ACTATATTTG GGTCAGGCAT AATGACAGCA GATGATGGAT CTGGATACAT TTGTGATGGT

1441 GATTCTACTC AGTCATCAAA TGGTTCTGGG GCTTCTGCAG TTGAAGGCAT CCCTGTTTTC

1501 TTGAGTGCAA TAAAAGAGTG GGTGATTGAG TTTGGGTCCT CGATGATTTC CATTTCAATA

1561 CGAACGGATA TGGCCTGGTA CAGGCTTGGA AAGCCTTCAA AACAATACGC TCCTTGGTTT

1621 GAGCGAGTCT TGAAAACTGC GAGGCTTGCC ATCAGTATCA TTACCATGTT GAAGGAGCAG

1681 ATCCGGGTGT CAAGACTTTC TTTTGCTGAT GTAATCAAGA GAATCTCAGA ACTTGCCAAG

1741 GATCACCCTG CTTTTATATC ATCTACTCTA CAAGTAGTGG AGAGATATGT GGTTGTGCAT

1801 GGTCAGATTA TTCTGCAGCA ATTTTCTGAA TATCCTGATG ATATTATAAA GAAGTGTGCT

1861 TTTGTAATTG GCCTTGCCAA GAAAATGGAA GAGAGGCACC ATACCAAATG GCTTGTCAAG

1921 AAAAAGAAGC TTCTGCATAG GAATGAACAG AACTTGAATC CCAGGGCTGC TATTGAGCCT

1981 GTTGTGTCCA AAAGGAACGC TATGCAGGCA ACTACAACAA GGCTAATCAA CAGAATCTGG

2041 GCAGGATACT ATTCAAATTA CTCACCCGAG GCACGAGATG GAGTGAATGG TGAAGTAAAA

2101 GAGGTTCATG AAATCGAAGA GCAAGAGGAA AACGAAGAGG ATGATGCTTT GGAGGAGAAA

2161 TTGGTTGTAT TTGAACAAAC TCAGACAACT AAGTCGGCTC CAAGGCAAAC CAAGTTAAGT

2221 TCATGTGTAG AGGAAGTAAA GTGGGGTGGG GAATCTGTAG GCAAACTACT TAATGGTGAG

2281 GATTTATATA AGAGGGCCAT AGTTCATGGT AATGAAATTG TGGTCAGGGG GGCTGTTCTT

2341 CTGAAAGATG ATGAAATGGA TGATTTTCCA CCCATCTATT TTGTAGAATA CATGTTTGAA

2401 AAGTCAAATG GAACTAAAAT GTTTCACGGG AGATTGATGC AACGGGGCTG CCAAACTGTG

2461 CTTGGAAACA CAGCTAATGA AAGGGAGGTA TTTTTGACAA ATGAGTGTAT GGATTTCCAA

2521 CTACAAGAAG TCAAAGAAAG TATATTTGTT GATCTAAGAT CAATTCCTTG GGGTCATCAG

2581 CACAGAAAAG AAAATGCTAA TGCTGAGAAA ATTGATAGAG CAAGAGCAGA GGAAAGAAAG

2641 AAGCAAGGAT TGCCTACTGA ATATTACTGC AAAAGTCTGT ATTGGCCTGA GAGAGGTGCC

2701 TTCTTTACTC TTCCATATAG TTCGTTGGGG CTTGGATCTG GCTCTTGTGA GGCTTGCAAT

2761 ATAAAAGAAA GTGAAAGGCA AAAGGAAAAG TTTATATTGG ATGCATCTTT GACCAGTTTT

2821 ATATACCAGG GAACTAAATA TTCTATAAAT GACTATATCT ATGTGAGTCC TTCATACTTT

2881 TCATCAGAAG AAATGGAGGC TGAGATTTAC AAGGCTGGGA GAAATGTCGG ATTGAAGGCG

2941 TATGTAATAT GCCAGCTGCT TGAAATTTGT GATCTCAAGA AGTCCAAAAG GAGCGAAGCC

3001 AATTCTGTTC AGGTCAAAGT CAGGAGATTT TTCAGGCCGG AGGATATATC ATCAGAGAAG

3061 GCATACTCGT CGGATATTCG AGAGGTCTAT TATAGTGAGG AGACACACAC CATACCTCTA

3121 GATGTTATTG AAGGAAAATG TGAGATCAGA AGAAAGAAGG ATCTTGGGCC TCAAGATATC

3181 CCCTTTACCT CTCACCATGT TTTCTTCTGT GAACATCTGT ATGATCCTTC CAGAGGATCC

3241 ATTAAGCAGT TGCCATCACA TATTAAATTA CAGTACTCAA CCGGGCAGCT GAATGATGAT

3301 GCCACATTTA GGAAAAAGAA AGGGAAATGC AAAGAAGGAG AGAATGATGA CAAAGAACCT

3361 GAGAAAGTAG TATCTGATGA GCACCGCTTG ACTACTTTGG ATATTTTTGC TGGCTGTGGT

3421 ACCTCTCTCA CAAAATGGGC TATTGAGTAT GATGCTGCTG CTGGAGATGC ATTTAAACTC

3481 AATCATCCTG GGTCTTTGGT ATTTGTAAAC AACTGCAATG TTATCCTCAG GGCAATCATG

3541 CAAAAGTGTG GGGATTCAGA TGATTGCATT TCCACCCCAG AGGCTGCTGA ATTGGCTGCA

3601 TCAATGGACC AAGTGGAAGT TGAGAACCTT CCACTACCCG GACAAGTTGA TTTTATTAAT

3661 GGTGGGCCTC CTTGTCAGGG TTTTTCTGGA ATGAATAGGT TCAATCAAAG CACATGGAGT

3721 AAAGTCCAAT GCGAGATGAT TTTGTCATTT TTATCCTTTG CTGATTACTA CCGTCCAAAG

3781 TATTTCCTTC TCGAGAATGT TAGGACCTTT GTATCTTTCA ACCAAGGGCA GACCTTTCGC

3841 TTAACTTTAG CTTCACTCCT TGAGATGGGA TATCAGGTGA GATTTGGTAT CCTTGAAGCT

3901 GGTGCATATG GAGTTCCTCA ATCTAGAAAG AGAGCTTTCA TATGGGCAGC GGCTCCTGAA

3961 GAGCTGCTTC CAGATTGGCC AGAGCCAATT CATGTCTTTT CAGCACCAGA GCTGAAAATC

4021 TCATTGTCGA AAAACTTGCA ATACTCTGCT GTCAGGAGTA CTTCAAAAGG TGCTCCATTC

4081 CGTCCTCTTA CAGTCAAAGA TACAATTGGA GATCTTCCAC CAGTGGTCAA CGGTGCATCT

4141 AACACAAGCT TAGAGTATCA AAGCGATCCA ATTTCGTGGT TCCAGAAGAA AATCCGTGGT

4201 AACTTGAACA TATTAACAGA CCACATATCA AAAGAAATGA ATGAGCTTAA TCTGATCAGG

4261 TGTCAGAAAA TACCCAAGCG CCCTGGTGCT GATTGGAGAG ATCTGCCCGA GGAGAAGGTC

4321 AAGTTATCTA CTGGCCAAAT GGCTGATCTG ATACCATGGT GCTTGCCCAA TACGGCCAAG

4381 AGGCACAATC AGTGGAAGGG ACTTTTTGGC AGGTTGGACT GGGAGGGGAA TTTTCCAACT

4441 TCAATAACTG ACCCTCAACC AATGGGTAAA GTTGGAATGT GCTTTCATCC TGATCAAGAC

4501 AGGATTGTGA CAGTCCGTGA ATGTGCACGT TCTCAATCTG CAAATTCTGT TTCCCTGACC

4561 ACATATTCCC TTGTACGTTC TGGCTTGTTC TTTTACACCA GAAGATCTTC AGAAACTGAG

4621 CTAGAACACA ATACAGAAGA TGTGGCTTCA CTTCCTTCTA CTCCTAATAA ACTTGGTTTC

4681 CCGGACAGCT ATAAATTTTT TGGTACTATT CACCACAAGC ACAGACAGAT CGGAAATGCT

4741 GTTCCTCCTC CTCTGGCTTA TGCCCTTGGA AGCAAACTGA AGGAAGCTGT CGAGAAGAAA

4801 AGAACGAGGA ACGCCTCTCC GACATCAACC GCGACAGGGG CAACGCCGTT CATCCGTTTC

4861 ACCTTTGCTG GAGAAATCTG GCAAATTCCT TATGATTTGG GAAAATTTAC AGTGTCGTTC

4921 ATCCGCTTTA TCCGCCACTT CCATCCTTCC AGATCTGGAG CCGCCCCCTG CTTCTTCGAT

4981 GAGAAACGCC TCTTCGACAT CAACCGTGAC AGGGGCAACG TTGTTCAATC ATAA

>SmMET1 protein sequence

1 MFAPMGHGLS FIEVKLPPAF TLPHLPTHTQ TPENAHSSAT STQPPPLLHR RDRSSSFAFA

61 SFRKRKQISS ASTKDMVTNQ KEKKKRASQT VEEPTVSRKM PKRAAACSDF KEKSVRISDK

121 DSVIETKKDG VVEEEVLAVR MTAGKEDGRP CRRLIDFTFH NSDGISQPFE MLEVDDIFIS

181 GLILPLEESA DKEKCKGIRC EAFGRIEEWA ISGYEDGSPV IWVSTEVADY DCIKPLGSYK

241 KFYDRFYAKA SACVEVYKIL SKSSGGNVSL DELLAGVVRS MSGMKCFSRG VSIRDFILSQ

301 VLTALKDESS KLVDFAEAQP VSLPGNLRIG PKIEDENKND LLAKPCLTEE DEDSKMARLL

361 QEEEDWRSMK QKKGRGSSSS SKYYIMINED EIANDYPLPA YYKMSNEETD EFIIFDSGID

421 VLHIEDLPRS MLHDWALYNS DARLVPLELL PLRPCDEIDV TIFGSGIMTA DDGSGYICDG

481 DSTQSSNGSG ASAVEGIPVF LSAIKEWVIE FGSSMISISI RTDMAWYRLG KPSKQYAPWF

541 ERVLKTARLA ISIITMLKEQ IRVSRLSFAD VIKRISELAK DHPAFISSTL QVVERYVVVH

601 GQIILQQFSE YPDDIIKKCA FVIGLAKKME ERHHTKWLVK KKKLLHRNEQ NLNPRAAIEP

661 VVSKRNAMQA TTTRLINRIW AGYYSNYSPE ARDGVNGEVK EVHEIEEQEE NEEDDALEEK

721 LVVFEQTQTT KSAPRQTKLS SCVEEVKWGG ESVGKLLNGE DLYKRAIVHG NEIVVRGAVL

781 LKDDEMDDFP PIYFVEYMFE KSNGTKMFHG RLMQRGCQTV LGNTANEREV FLTNECMDFQ

841 LQEVKESIFV DLRSIPWGHQ HRKENANAEK IDRARAEERK KQGLPTEYYC KSLYWPERGA

901 FFTLPYSSLG LGSGSCEACN IKESERQKEK FILDASLTSF IYQGTKYSIN DYIYVSPSYF

961 SSEEMEAEIY KAGRNVGLKA YVICQLLEIC DLKKSKRSEA NSVQVKVRRF FRPEDISSEK

1021 AYSSDIREVY YSEETHTIPL DVIEGKCEIR RKKDLGPQDI PFTSHHVFFC EHLYDPSRGS

1081 IKQLPSHIKL QYSTGQLNDD ATFRKKKGKC KEGENDDKEP EKVVSDEHRL TTLDIFAGCG

1141 TSLTKWAIEY DAAAGDAFKL NHPGSLVFVN NCNVILRAIM QKCGDSDDCI STPEAAELAA

1201 SMDQVEVENL PLPGQVDFIN GGPPCQGFSG MNRFNQSTWS KVQCEMILSF LSFADYYRPK

1261 YFLLENVRTF VSFNQGQTFR LTLASLLEMG YQVRFGILEA GAYGVPQSRK RAFIWAAAPE

1321 ELLPDWPEPI HVFSAPELKI SLSKNLQYSA VRSTSKGAPF RPLTVKDTIG DLPPVVNGAS

1381 NTSLEYQSDP ISWFQKKIRG NLNILTDHIS KEMNELNLIR CQKIPKRPGA DWRDLPEEKV

1441 KLSTGQMADL IPWCLPNTAK RHNQWKGLFG RLDWEGNFPT SITDPQPMGK VGMCFHPDQD

1501 RIVTVRECAR SQSANSVSLT TYSLVRSGLF FYTRRSSETE LEHNTEDVAS LPSTPNKLGF

1561 PDSYKFFGTI HHKHRQIGNA VPPPLAYALG SKLKEAVEKK RTRNASPTST ATGATPFIRF

1621 TFAGEIWQIP YDLGKFTVSF IRFIRHFHPS RSGAAPCFFD EKRLFDINRD RGNVVQS

>SmCMT1 genomic sequence

1 ATGGGAAGAG GTAAAAAGCG AGCGGAGGCA ACATCCTCGC CGCCGCCACC TATTTCTGCA

61 TCACCGAAGA AGCCGAAGAG ATCCACGCCA GTCGACACCG AGCTCGGTTT CATCAGTTCT

121 CCAGTTCCCT CCCACGAAGC CAAATCTAGA TGGCCTCATA GATATCAAAC TAAGGTTCAG

181 CTGTTTAACT GAACCGAAGC TTTATATTCT GCGCTTCATT TAAGCTGTCT TCGTATTTGA

241 GTCAATCTTC CATTACCATT TTTCTTGTTT TGAATTTTAC AGAATGTGAA GACTACAGTA

301 ACTGTTACTT TATCAGATGG CTCGACTGAG TAAGCTTGAC CTATTATGTG CCCATTCCTG

361 TGCCTTATGC TGTTTTTAGT TGTTGAAATG TTTTCTGTTG CAATCTTTTA ACAGGGAAAA

421 AGAGCTTTTT CAAGCTAGAT CTCACTATAC AAGAGCTGTT GTTGATGCGG TTGTGTTTGA

481 ACTCTTTGAC GATGCTTACA TTCAGGTTAT TCAACTTTTT TTTCTGTTTG GGGCTTGAGA

541 GAAGATATAG TTTTCTTAAA TTTTGATTTT CTAGTAGTCA CTCTTCACAT ATAGTTGATG

601 TTGATTTGAT GACATACTTC CAGGCTTCAG AAGGTGAACT GGATTTTATT GCAAAGATTG

661 TGGAAATGTT CGAAACCACG AATGGCGAAA TGTATATTTG CACCCAATGG TTTTATCGAG

721 CCAAAGATAC GGTGCGAGAA CTTTTGTATA ATAGACCGAG TTCAACTGCT TATGCAAAAT

781 GGTGAAATAA TTTTTGGCTT TTATGCTTTC AGGTCATAAA GAATCAAGAT TACCTAATTG

841 ATGCAAAACG AGTCTTTTAC TCGGACATAA GGGATGACAA CCCGTTAAGC TGTATCAGTA

901 GAAAAGCTAA AATTAAGCAA CTTCCACCAA ATGTAAGGAA CAAAAATTTG TCCCTTTTAA

961 CCACTTCATT TTTATTTGAG CTGCAATCTT TGAGCAGATG GCTGCTGCTG CAAGAGAAAA

1021 AGCTATGGAC TCCTATGATC TTTATGTTGA TGCGATGTAC ACTTTCCCAC ATGCTTTTTC

1081 AAATTTAAGC AAAGGTAAAC TGTTGCTAGA TGTCCATTAT ACTAATGATA ATCAGTTTTA

1141 CTTTATTTCC CCCTTTTATC TTTTGGTTGT TAATGTGATA TATTTTTTTT GTTTAAAAAA

1201 AAACTAAAAC GCAGACATAA ATAACTCACC ATTAACTAAT GGAGTGATGG AAACAAAAAC

1261 TGAAGATGTC AAAAAATCTC CAGAAGATTC TCAGCGATCA GAAATTACTG TATTGGATTT

1321 GTACTGTGGT TGTGGTGCAA TGTCCACTGG ACTTGGTATT GGTGCATCTT TGCTCGGAGA

1381 GAAAATTGTC ACGGTAAGGA AAAGTGACTA AAAGTAGCAA CTACAAACTT TTCTTGATAT

1441 TTTATCATAG TTTTCACTTG GCATTCCTTT TTTGATAAAC AGAAGTGGGC TGTTGATTTC

1501 AATTCCTATG CCTGCGAGAG TTTAAAATAT AACCATCCAA ATACAGAGGT AGGCTGTCAC

1561 ATTTTTGTTG AGTTGCAAAA GGTTATTACT TTTATGGCAG TATAAAACAA ATGTTAGTGA

1621 ATTTTTCAGG TAAGAAATGA AGATGCAGAG AACTTTTTTG ATCTTCTAAA AGAATGGGAA

1681 AACCTTTGCA CAAAGTTCAA TGTGGTGGGA TCAAAAGATA TTGAACTAGA GCCTCAAGAT

1741 TCAGACAGTG AGGCATCTGA AGTAGACGAT GGAGTGGTGA TTCCATCTGA AGGAGAGTAT

1801 GAAGTGCAGA AGTTGCTCGC CATTTGCTAT GGGGATCCAA ATAAAAGCAA AAAAACTGGT

1861 GTATATTTCA AGGTACTTTC TTTATTGTTA TTAACTAGCA TACGGTCCAC TTAGGAGCTA

1921 TTGTACATAA AAGATGGTTA ATGGGGTTTT GTTTGTACTA AATTTTCATT AATATCTAAC

1981 CATAGAAGGG TAAGAGCTAT TGGTTTCGCA AAGACACAAT GGGTTATGTT TTGCACAGAG

2041 CATAGGGAAA TTGCTTTTTA CCTAACTTTT AAGCAAATTC TTGTTTTTAA ATTTTTAGGT

2101 ACGCTGGAAA GGTTATGGCC GTGCTGATGA CACTTGGGAG CCTATTGATG GCCTAAGGTT

2161 TGAACTCTAA TATTTCCTCT ATTACCCCAT CGGCCACCAG TCACTACAGC TAAATGATAT

2221 CTTCATGTTT CTAACCATCT TTTCTCATAT TCTGTTATTT TATGAAAAAG TAACTGCGAA

2281 GAAAGGATAA AGGAGTTCGT CTTAAGAGGT TATAGAGCTA GAGTATTACC TCTTCCGGTA

2341 AGTTTTATTC CTTTCCATGA ATTGTGCTTA AGTTTAACTT CATCTTGTTC CTTTCATGTA

2401 GCTTTTTGTT TCCTAATGTT TTGTCAGGGT GATGTTGACT TTATATGTGG TGGTCCTCCA

2461 TGTCAAGGAA TTAGTGGCTT CAACCGATTT AGAGATGCTC TTGCTCCTTT AAAGGACAAA

2521 AAGAATCACC AACTTGTCGT CTACATGAAC ATTGTGGAGT TTTTGAAACC AAGATATATA

2581 TTGATGGAGA ACGTTACAGA TATTTTGAAA TTTGCAAAAG CACAGTTGAC TAGTTATGCT

2641 GTTGGGCGAC TAGTTTCTAT GAATTACCAA GCCAGATTAG GTATAATGGC TGCTGGTGCA

2701 TATGGAGTTC CACAATGCCG AATGCGATTT TTTCTTTGGG GCGCTGGGCC AACAGAGGTA

2761 AGTTTATTAC TCTTGATGAG CTAGTTAATA CCACTAATGC TCATATCCAT AACTAGTTAT

2821 TTGACTTTCC AGATTTTACC TCAATTTCCA CTGCCCACAC ATGAAGTGCT GAAAAAAGGA

2881 GTTGTCATAA ATGAATATAA GGTGTGGCTT ATATCAAGTC TGCTTAAAAC ATTCTGTCAT

2941 TTGTACTGTT CTAGAACATC ATTTTGACTT TGATATAGGA ACTTATAATC GGACATGGAG

3001 AAGAGCATTG CCAGTTGCAT AAGGCTGCTT TACTAGGGGA TGCTATTTCT GATATGCCAG

3061 AGGTACAAAC CTCTATTGAT TTAGAAATTT TGAACCTAAT TTTTTTCTTA TTCTTGTTCA

3121 CCAAATTTTT ATTACCATAC TTATTTCTTT CATGCTTATG TCTCTTTAGG TGACAAATAA

3181 CACAGATCAA GATGAAATGC CTTATGGTAA CGCTCCTCGT ACTGAATTTC AGAAGCTCAT

3241 TAGATTGAGC AGACAAAGTG AGTATAATTA TGAAGACTTC TTAGAAATTG TCTCACTTCT

3301 TTCAATTATC ACGTCTGGAG TTGAATATCT CTCTGTATAT GTAATAGTTT GATATTGTCT

3361 AAATATACAT GATATCAAGT TGACGTGTCT TCTCTTGAAT TATAAATGTT TTCAATTAAA

3421 TATAGTTTTT CACTTTTTTC CTACAAGTCC ATAATCTTAG TTTGAGGTGC TTCTAATGCT

3481 TAGAAATGAT TCAAAGGAAA GATTCTCTTA AGTCCACTAG TGTGGCAAAT AATTGAATAT

3541 TGATTATGTA TTCCTTTGAG ATGCATATTT CTGAGGATTG ATCTTTCTTT ATTCTTTTAT

3601 CATTTTCTCT CCAGAGATAT TTGGTTATTC TGATGCTAAA AAGAAAAATT CCCACAAGTC

3661 CATATTGCAT GATCATCGTC CTTATCAATT GAATCAAGAT GATTATGAAA GGGTTTGCCA

3721 TGTTCCCAAG TTCAAGGTTG GAGATTTTTT GTTTGTTTCT AAATATTAGC AACTAGAATT

3781 AATTTGTCAA TTGGACTTCC TTGTTGCTTC TGTATAGCTT TTTGCTACAA AATAATTCTG

3841 ATTATTTCAT AACTACAGGG AGCTAACTTT AGAAACTTAC CCGGTGTATT GGTTGGACCG

3901 GATAGAGTCG TAAAACTTGA TGAATCTATC GATAGACCTC GGTGCAAGTC AGGCAAACCT

3961 TTGGTATTCA TCATTGCAGA ACTATTTGAT CATCTTCGGA CCTTTTGAAG TTCATTCTAG

4021 GTTAACTTAT GTTATGTATA AACAGGTACC AGATTATGCC TTAAGTTTTG AAGATGGGAA

4081 GTCTTGCAAG TAAGATTTTG TGATTTGATG TGTCTTTAGC CATTCTTGAC ACTGCACTAT

4141 CTAACCTGAA ACCTTATGGT CTATAGGCCT TTTGGTCGAT TAGGGATGGA TGACATCGTG

4201 CCCACTGTAG TTACAAGGCC CGAACCCCAT AATCAGGTTT GGATTGTGAT AAATAGTCAA

4261 TACTGAGGAA TGCATTCAAT ATTAACTACT TCAAATTTAT TTTGTTTTTC TTAATAGGTT

4321 CTGTTGCATC CAAATCAAGA TAGAGTGCTC TCTGTCCGAG AAAATGCAAG GCTACAAGGA

4381 TTTCCAGATT GCTATAAGCT TTTCGGACCA GTGAGAGAGA GGTAGGATTC GATTGTAATA

4441 TGTTACAAGT GTAGTGTGCT AGTGCTTCAT GTAGATATCT TACGTTGTAT TTCATTTGTT

4501 TCAGGTACAT GCAAATCGGA AATGCTGTTT CGTTTGCTGT TTCAATACCA TTAGGCTATT

4561 GCTTGGCTAA AGCACTGCAA GGTGCACAAC TCACAACACC ATTGAGCCTT CCATTCAAGT

4621 TCCCCGACTG CCTCGGCCAG TTGCAGTCTC TTCGACAAGA ACCAGAAGAG CTTGTTTCAG

4681 AGTGA

>SmCMT1 ORF sequence

1 ATGGGAAGAG GTAAAAAGCG AGCGGAGGCA ACATCCTCGC CGCCGCCACC TATTTCTGCA

61 TCACCGAAGA AGCCGAAGAG ATCCACGCCA GTCGACACCG AGCTCGGTTT CATCAGTTCT

121 CCAGTTCCCT CCCACGAAGC CAAATCTAGA TGGCCTCATA GATATCAAAC TAAGAATGTG

181 AAGACTACAG TAACTGTTAC TTTATCAGAT GGCTCGACTG AGGAAAAAGA GCTTTTTCAA

241 GCTAGATCTC ACTATACAAG AGCTGTTGTT GATGCGGTTG TGTTTGAACT CTTTGACGAT

301 GCTTACATTC AGGCTTCAGA AGGTGAACTG GATTTTATTG CAAAGATTGT GGAAATGTTC

361 GAAACCACGA ATGGCGAAAT GTATATTTGC ACCCAATGGT TTTATCGAGC CAAAGATACG

421 GTCATAAAGA ATCAAGATTA CCTAATTGAT GCAAAACGAG TCTTTTACTC GGACATAAGG

481 GATGACAACC CGTTAAGCTG TATCAGTAGA AAAGCTAAAA TTAAGCAACT TCCACCAAAT

541 ATGGCTGCTG CTGCAAGAGA AAAAGCTATG GACTCCTATG ATCTTTATGT TGATGCGATG

601 TACACTTTCC CACATGCTTT TTCAAATTTA AGCAAAGACA TAAATAACTC ACCATTAACT

661 AATGGAGTGA TGGAAACAAA AACTGAAGAT GTCAAAAAAT CTCCAGAAGA TTCTCAGCGA

721 TCAGAAATTA CTGTATTGGA TTTGTACTGT GGTTGTGGTG CAATGTCCAC TGGACTTGGT

781 ATTGGTGCAT CTTTGCTCGG AGAGAAAATT GTCACGAAGT GGGCTGTTGA TTTCAATTCC

841 TATGCCTGCG AGAGTTTAAA ATATAACCAT CCAAATACAG AGGTAAGAAA TGAAGATGCA

901 GAGAACTTTT TTGATCTTCT AAAAGAATGG GAAAACCTTT GCACAAAGTT CAATGTGGTG

961 GGATCAAAAG ATATTGAACT AGAGCCTCAA GATTCAGACA GTGAGGCATC TGAAGTAGAC

1021 GATGGAGTGG TGATTCCATC TGAAGGAGAG TATGAAGTGC AGAAGTTGCT CGCCATTTGC

1081 TATGGGGATC CAAATAAAAG CAAAAAAACT GGTGTATATT TCAAGGTACG CTGGAAAGGT

1141 TATGGCCGTG CTGATGACAC TTGGGAGCCT ATTGATGGCC TAAGTAACTG CGAAGAAAGG

1201 ATAAAGGAGT TCGTCTTAAG AGGTTATAGA GCTAGAGTAT TACCTCTTCC GGGTGATGTT

1261 GACTTTATAT GTGGTGGTCC TCCATGTCAA GGAATTAGTG GCTTCAACCG ATTTAGAGAT

1321 GCTCTTGCTC CTTTAAAGGA CAAAAAGAAT CACCAACTTG TCGTCTACAT GAACATTGTG

1381 GAGTTTTTGA AACCAAGATA TATATTGATG GAGAACGTTA CAGATATTTT GAAATTTGCA

1441 AAAGCACAGT TGACTAGTTA TGCTGTTGGG CGACTAGTTT CTATGAATTA CCAAGCCAGA

1501 TTAGGTATAA TGGCTGCTGG TGCATATGGA GTTCCACAAT GCCGAATGCG ATTTTTTCTT

1561 TGGGGCGCTG GGCCAACAGA GATTTTACCT CAATTTCCAC TGCCCACACA TGAAGTGCTG

1621 AAAAAAGGAG TTGTCATAAA TGAATATAAG GAACTTATAA TCGGACATGG AGAAGAGCAT

1681 TGCCAGTTGC ATAAGGCTGC TTTACTAGGG GATGCTATTT CTGATATGCC AGAGGTGACA

1741 AATAACACAG ATCAAGATGA AATGCCTTAT GGTAACGCTC CTCGTACTGA ATTTCAGAAG

1801 CTCATTAGAT TGAGCAGACA AAAGATATTT GGTTATTCTG ATGCTAAAAA GAAAAATTCC

1861 CACAAGTCCA TATTGCATGA TCATCGTCCT TATCAATTGA ATCAAGATGA TTATGAAAGG

1921 GTTTGCCATG TTCCCAAGTT CAAGGGAGCT AACTTTAGAA ACTTACCCGG TGTATTGGTT

1981 GGACCGGATA GAGTCGTAAA ACTTGATGAA TCTATCGATA GACCTCGGTG CAAGTCAGGC

2041 AAACCTTTGG TACCAGATTA TGCCTTAAGT TTTGAAGATG GGAAGTCTTG CAAGCCTTTT

2101 GGTCGATTAG GGATGGATGA CATCGTGCCC ACTGTAGTTA CAAGGCCCGA ACCCCATAAT

2161 CAGGTTCTGT TGCATCCAAA TCAAGATAGA GTGCTCTCTG TCCGAGAAAA TGCAAGGCTA

2221 CAAGGATTTC CAGATTGCTA TAAGCTTTTC GGACCAGTGA GAGAGAGGTA CATGCAAATC

2281 GGAAATGCTG TTTCGTTTGC TGTTTCAATA CCATTAGGCT ATTGCTTGGC TAAAGCACTG

2341 CAAGGTGCAC AACTCACAAC ACCATTGAGC CTTCCATTCA AGTTCCCCGA CTGCCTCGGC

2401 CAGTTGCAGT CTCTTCGACA AGAACCAGAA GAGCTTGTTT CAGAGTGA

>SmCMT1 protein sequence

1 MGRGKKRAEA TSSPPPPISA SPKKPKRSTP VDTELGFISS PVPSHEAKSR WPHRYQTKNV

61 KTTVTVTLSD GSTEEKELFQ ARSHYTRAVV DAVVFELFDD AYIQASEGEL DFIAKIVEMF

121 ETTNGEMYIC TQWFYRAKDT VIKNQDYLID AKRVFYSDIR DDNPLSCISR KAKIKQLPPN

181 MAAAAREKAM DSYDLYVDAM YTFPHAFSNL SKDINNSPLT NGVMETKTED VKKSPEDSQR

241 SEITVLDLYC GCGAMSTGLG IGASLLGEKI VTKWAVDFNS YACESLKYNH PNTEVRNEDA

301 ENFFDLLKEW ENLCTKFNVV GSKDIELEPQ DSDSEASEVD DGVVIPSEGE YEVQKLLAIC

361 YGDPNKSKKT GVYFKVRWKG YGRADDTWEP IDGLSNCEER IKEFVLRGYR ARVLPLPGDV

421 DFICGGPPCQ GISGFNRFRD ALAPLKDKKN HQLVVYMNIV EFLKPRYILM ENVTDILKFA

481 KAQLTSYAVG RLVSMNYQAR LGIMAAGAYG VPQCRMRFFL WGAGPTEILP QFPLPTHEVL

541 KKGVVINEYK ELIIGHGEEH CQLHKAALLG DAISDMPEVT NNTDQDEMPY GNAPRTEFQK

601 LIRLSRQKIF GYSDAKKKNS HKSILHDHRP YQLNQDDYER VCHVPKFKGA NFRNLPGVLV

661 GPDRVVKLDE SIDRPRCKSG KPLVPDYALS FEDGKSCKPF GRLGMDDIVP TVVTRPEPHN

721 QVLLHPNQDR VLSVRENARL QGFPDCYKLF GPVRERYMQI GNAVSFAVSI PLGYCLAKAL

781 QGAQLTTPLS LPFKFPDCLG QLQSLRQEPE ELVSE

>SmCMT2a genomic sequence

1 ATGAAGGAAC AGCAGAAGCT ACCGGACCAA CTCCCGCTCA GAAAATCCTC AAGCATTTCT

61 TCTTCGCCTT CAGCTCGTCT CTGCAACGGA GCAAAACTCG AACCCGAAGC CCGGCCCGTT

121 GATGCATTGG CCCTTTCATT GTATGTGGCG GAGGCGGAGC AGCAGCAGCA GCCACTGAGC

181 ATCTGCTACC CTTCTCATGA AGAGGAAGAA GAAGATAATT CGGTCCGCAG ATCGCCGAGA

241 TTTACATCCA ACAAGTACAA ATATCTCTCG CCGGAACGAA GCAGATCGCA GAAGCATACC

301 GCATTGGTTG TATCGAAATT GAGTATGTCG CCGAGAATCT TCTTCTCTTC ATCGGCTCCG

361 GCGTTGACTT TGACGGACGG TGGGAATGTA GAAGCGGGGC CCAAGAAAAG GCGCCGGCTT

421 CTTGCATTGC CCGGACCGGC GGGGAGTGGA GTTTGTTTGA GCGAGCGGTG CTTGAGATCG

481 AGAACTGTCT TGATGCGAGT CGCAGAATCG CCGGAGGCTT CTGGTGCGAA GAGGTTGAGG

541 TCAGTAGGTG AGAGGAGAGA GAAAGGGGAA GCAGCTGCAG AAAATGCTGG TAATTATGCT

601 TTGATTGATT GCGTCAAAAT GAGGTGGAAG CCCACAAATT GAGATGCTCG CATTGCCTCA

661 GGCAACCCCA ATTTCAAGCA AGAACCGCAC AAGACCAAGT GCTTCTTTGA ATAAGGAGTG

721 CTCAGTGACT CCGGAAAAGG AGTCATCTTT AGCGGGAGTG AGTGAGAAGC AGTTGAGGTC

781 TAGAAAATTC AGTTTAAACA GAATGTGGCT GAGAATGCTG ATCCGAATGG AGGAGGGCGT

841 AATGGAGAGA GCGCGGTGAA GTCGAAGAAA AAGGAGAAGA GCATTTTGTG CTGTTTTATA

901 GGAGAGCCAA TTCCGGAGGA AGAAGCTCGG GAAAGATGGC AATGGAGATA TGATTTAAAG

961 GTGAGTGCTT GCTGATTAAG TCATTCCAGT GTAATGATAT GTGTAGCTTA TTGTTGGTCT

1021 TGCTACTGAA TTTGGGTCCT CAAATTAGTT GATTTAACAT GCATGGTTGA AAAAAGCCTC

1081 TGTTAATTGA TATCATTCTT GTCCATGTAC TCTTAATACA CAAGAAACTG TAGAATAATA

1141 CACAAGAAAT CTAAGAGAAT GAAAGCCATA GGCTCTGCTG CCAAATACAT GATTGAGGAT

1201 GATCTATAGT ACTTCCCTTG ATTCCAGACA TGTCCTTTTC TAATGCTGCT TGGACATGCC

1261 ATCCATCTGT AGCTATTTGG AGCAAAATAA TGGTATCGAG CATGTGAGAT TCGTACAACT

1321 TTTGTTAGCG CTAGGGAACT GCAATGTGTG ACGTATATAC TTCCTTTTTG AAAGAAAAGT

1381 CTCAGTTATT ATATGGACTA GTAAAGCCGA AGTAGACTGT CTGTTTGTTT TCGTTCAGCA

1441 GTCCCCGATA GACTGTAGCC GAGTGCTCAT CATTGAAGAA AGAACATATG TTCATAGTTT

1501 TCATTGTCTT GAAAGAGTCA CTAAGTGTTG TGTGTATTAT TGCATTTATC TGGCTTATCA

1561 GAATTGTTCT AGACAGTGAC ATGAACATTT CTAACTACAA TTGATTTTGG ACTCCACAGA

1621 GCAGAATAAA GGGCAAAAAC TGGAAAATAA AGTGAGTGAA CCCTACGAAC ATATGTTTCT

1681 GGTGGTAACA TAATATGGCC CTCTACACCT TCATCTCTCA TATTTTCAAG TGTTTCTAAA

1741 TCTTATAATT TGCTTATTGA CTTTCAGCGC TGAAGAAGAT GAAATCATAC TGAATGTGGC

1801 AAGCCATTAC GCTCAAGCCA AAGTAGGGAA CTTTGTCTTG AATATTGGAG ATTGTGCGTT

1861 TGTAAAGGTA AATCTCGTGT CATTTCTTAT GTCTTGTCAC GTTTGTTACT CTTTAGTGCT

1921 GCTGTAAGTT TTAGTCGTAG AAAATGTTTC ACACTATTGT CTGTAGAATA TAACTAAAGG

1981 TGATGATTCC CATTTTCTCA TGGCACCATA TGAATTCGGT GAAACTTCAT CTTTCGTTTG

2041 ATTCTTAGTT ATTGGAATTC TAGACAGTAT GCGATTAGCA ATCACTTATC TGGTTATGGT

2101 TTAATATTGC AAGATTGATG TCCTAAGCAA TTCCTTCAGG GTGAAGGGAA CAAGAAACAC

2161 ATAGGGAGGA TCTTGGAATT CTTCAAAACG ACGGAGGGAG AAGACTACTT TAGGGTTCAA

2221 TGGTTTTTCA GAGCCGAAGA TACGGTTAGT ATAACATGCT TCCTGTTTTT GTTTCCTTCA

2281 TTCTATTCTC ACCTGTTTCA TGTTCTTTGT TTGCCCGTGG ACCATCATTC ATCAGGTTCT

2341 GAAAGACACT GCTTCATCGC ATGACAAAAG GCGCATATTC TACTCCATCT TAATGAACGA

2401 CAACGTGCTA GATTGCATCA TTTCAAAAGT CAAGATAGTA AACGTACCCC CTATGGTATA

2461 TTACCCTTTG TTACCCTCTG TCGTCTATCT TATTTACTCA TTTCTATTAC AGTCTTTGCC

2521 ACTTTTTACT ATTCTACGGT TTTTCTTCTC ATCAAACTGC AGCCGTGCTT AAAAAGAAAC

2581 CGTGTCCAGT CAGCTGCATT TTATTATGAT ATGGAGTACT CTGTGCAATA TTCAACATTT

2641 CGTACAATGG TTACAGGTTA GTAGTACGAT AAAAAACACG TTCTTTTCTC AGTGCACTCG

2701 TTGCTTGCTT CATAATGTGG CCAGTACATT ACTGTCTAGC TGCTCTCTGC TTCTCCTGAT

2761 CAGCTAGATT TCTGTTTTGA TTAGCTTATA TTCTATTGTC ACATTATATT TCAGAAAAGT

2821 CTATGGGGAA TGAGTCATTC TTGTCGAACT CAAAACATCC CGATGATGGG CCAATTACTT

2881 TGACACCCTT GGAGCTTTTG CCTGGTTACG ATTCTCCCAA ACAAGAATTA ACACTACTGG

2941 ACCTGTACTC TGGATGTGGT GGAATGTCCA CTGGATTATG CCTTGGTGCC AAACTGTCGA

3001 GTGTAAATCT CATGACGGTA ATCACGAAGA TGAAACTTCA ATTGTAATTC ATGATTTAAA

3061 TCCTGGTGAT CTTATCATCA GTTTAACTCA CTTCCTCCTT GTTTTTCAGA AATGGGCTGT

3121 TGATTATAAC AGCTCTGCTT GTGAGAGTTT GAGGCTGAAC CATCCAGAAA CAAATGTATG

3181 TAACTTGAAA ATCACATTTT GATGAAGTTA TTCTGTTTGA TTATGTAAGG TTACCTTAGA

3241 TATCTGAATT CTTGTACAGG TAAGAAATGA ATCTGCAGAG GATTTCCTAG ACCTACTAAA

3301 GCAATGGGAG AGTTTATGTA AGAGATACGT CTCTGATATA GAAAGAACAC TGAAATGTGA

3361 GTTAGAGGAA CTTCCAGAAG CCGAAGCCGA AGACTGTCTG ATTCCATCGG ATACAGAGGA

3421 ATCTAGTGGG GAATACGAGG TCTCTCGCCT AGTTGATATT TGCTACGGCG ATCCTAACAA

3481 CAGTGGAAAA CGCGGGCTAC ACTTCAAGGT ACGTTGGAAG GGGTACGGCC CGGATGATGA

3541 TACATGGGAA GCAGCTGAAG GATTGAGGTG AGGGACTATA CCTAACATAT ACAGAAGCAA

3601 TCCTTTATGT CGATTCACTT CTTTCTAACG CGTCTTGTCC GTTTTCAGCA ATTGCCAAGA

3661 GCGGATAGAG GACTTTGTTC GAAGAGGATT CAACGCTAAA ATCCTGCCTC TCCCTGTGAG

3721 TTCAATATGT CAAGCAGCAA TGCAGCAAGT AGTGTTGATT GTGTTAAATA AGAAGCAATG

3781 TTGGTTGTGT TGGTGTTGTG CAGGGCGATG TGGGTGTTAT CTGCGGAGGG CCGCCGTGCC

3841 AGGGCATAAG CGGCTACAAT CGTCACAGAA ACTTCGCGTC TCCGTTGGAA GACGAAAGAA

3901 ACCACCAAAT CGTGGTGTTC ATGGACACAG TGGAGTTCTT GAAACCCAAG TATTGCTTGA

3961 TGGAAAACGT GGTCGACATC ATAAGGTTCG ACAAGGGCTC TCTGGGTCGA TACGCTCTAA

4021 GCCGTCTGGT GCTCATGCGA TACCAAGCGA GGCTCGGCAC CGTCGCCTCC GGCTGCTACG

4081 GCCTCCCGCA GTTCCGCCTG CGCGTCTTCA TCTGGGGGGC GCACCCGGGC GAGACCCTGC

4141 CGCAGTTCCC GCTCCCCACG CACGATGTCG TCGCCAGGTA CTGGCCTCCC ATCGAGTTTG

4201 AGCGGAACAT CGTGGCGTAC GACGAGGGCC AGCCCCGCGC CCTGCACGGC GCCATCGTCC

4261 TCGGGGACGC CATCTCCGAT CTCCCGCCCG TCACCAACGA CGAGGACCGG GAGGAGATGG

4321 CGTACGGGAA GCCCCCGGAG ACGGAGTTCC AGAGGCGCAT CCGGACGGGG GCCGGGGACG

4381 TCCTCTACGA CCACCGGCCG CTGAAGCTGT CGGAGCACGA CTACATGCGC GTGTGCCGAG

4441 TCCCGCACAG GAAGGGGGCC AACTTCAGAG ACCTCGAGGG GGTGGTGATC GGAGAGGACA

4501 ACGTGGCGCG GCGCGATCCG ACGAGGGAGG CGGTGATGCT GCCGACGGGG AGGCCGCTGA

4561 TCCCGGAGTG CGTGTTCAAG TTGGAGCTGG GGAAGTCGAG AAGGCCTTAT GGGAGAGTAT

4621 GGTGGGATGA GACGGTGTCG ACGGTGGTGA CGTTTCCTCA TATTCGAGCA ATGGCGATAC

4681 TGCATCCGGA GCAGGATAGG AGGCTGAGCA TAAGGGAATG CGCGAGGCTG CAGGGATTCC

4741 CGGACTACTA CAGATTCTGC GGCACTATAA AAGAGCGGTA CAGCCAGGTA GGGAACGCCG

4801 TCTCAATTCC GGTGGCGCGG GCCTTGGGCT ACGCGCTCGG GATAGCCTAC CGGAAGCTCG

4861 CCGGAGAAGA CCCGCTCATG ACGCTGCCGC CGGACTTCTC TTCTTCTACT TGA

>SmCMT2a ORF sequence

1 ATGAAGGAAC AGCAGAAGCT ACCGGACCAA CTCCCGCTCA GAAAATCCTC AAGCATTTCT

61 TCTTCGCCTT CAGCTCGTCT CTGCAACGGA GCAAAACTCG AACCCGAAGC CCGGCCCGTT

121 GATGCATTGG CCCTTTCATT GTATGTGGCG GAGGCGGAGC AGCAGCAGCA GCCACTGAGC

181 ATCTGCTACC CTTCTCATGA AGAGGAAGAA GAAGATAATT CGGTCCGCAG ATCGCCGAGA

241 TTTACATCCA ACAAGTACAA ATATCTCTCG CCGGAACGAA GCAGATCGCA GAAGCATACC

301 GCATTGGTTG TATCGAAATT GAGTATGTCG CCGAGAATCT TCTTCTCTTC ATCGGCTCCG

361 GCGTTGACTT TGACGGACGG TGGGAATGTA GAAGCGGGGC CCAAGAAAAG GCGCCGGCTT

421 CTTGCATTGC CCGGACCGGC GGGGAGTGGA GTTTGTTTGA GCGAGCGGTG CTTGAGATCG

481 AGAACTGTCT TGATGCGAGT CGCAGAATCG CCGGAGGCTT CTGGTGCGAA GAGGTTGAGG

541 TCAGTAGGTG GAAGCCCACA AATTGAGATG CTCGCATTGC CTCAGGCAAC CCCAATTTCA

601 AGCAAGAACC GCACAAGACC AAGTGCTTCT TTGAATAAGG AGTGCTCAGT GACTCCGGAA

661 AAGGAGTCAT CTTTAGCGGG AGTGAGGCGT AATGGAGAGA GCGCGGTGAA GTCGAAGAAA

721 AAGGAGAAGA GCATTTTGTG CTGTTTTATA GGAGAGCCAA TTCCGGAGGA AGAAGCTCGG

781 GAAAGATGGC AATGGAGATA TGATTTAAAG AGCAGAATAA AGGGCAAAAA CTGGAAAATA

841 AACGCTGAAG AAGATGAAAT CATACTGAAT GTGGCAAGCC ATTACGCTCA AGCCAAAGTA

901 GGGAACTTTG TCTTGAATAT TGGAGATTGT GCGTTTGTAA AGGGTGAAGG GAACAAGAAA

961 CACATAGGGA GGATCTTGGA ATTCTTCAAA ACGACGGAGG GAGAAGACTA CTTTAGGGTT

1021 CAATGGTTTT TCAGAGCCGA AGATACGGTT CTGAAAGACA CTGCTTCATC GCATGACAAA

1081 AGGCGCATAT TCTACTCCAT CTTAATGAAC GACAACGTGC TAGATTGCAT CATTTCAAAA

1141 GTCAAGATAG TAAACGTACC CCCTATGCCG TGCTTAAAAA GAAACCGTGT CCAGTCAGCT

1201 GCATTTTATT ATGATATGGA GTACTCTGTG CAATATTCAA CATTTCGTAC AATGGTTACA

1261 GAAAAGTCTA TGGGGAATGA GTCATTCTTG TCGAACTCAA AACATCCCGA TGATGGGCCA

1321 ATTACTTTGA CACCCTTGGA GCTTTTGCCT GGTTACGATT CTCCCAAACA AGAATTAACA

1381 CTACTGGACC TGTACTCTGG ATGTGGTGGA ATGTCCACTG GATTATGCCT TGGTGCCAAA

1441 CTGTCGAGTG TAAATCTCAT GACGAAATGG GCTGTTGATT ATAACAGCTC TGCTTGTGAG

1501 AGTTTGAGGC TGAACCATCC AGAAACAAAT GTAAGAAATG AATCTGCAGA GGATTTCCTA

1561 GACCTACTAA AGCAATGGGA GAGTTTATGT AAGAGATACG TCTCTGATAT AGAAAGAACA

1621 CTGAAATGTG AGTTAGAGGA ACTTCCAGAA GCCGAAGCCG AAGACTGTCT GATTCCATCG

1681 GATACAGAGG AATCTAGTGG GGAATACGAG CGGATAGAGG ACTTTGTTCG AAGAGGATTC

1741 AACGCTAAAA TCCTGCCTCT CCCTGGCGAT GTGGGTGTTA TCTGCGGAGG GCCGCCGTGC

1801 CAGGGCATAA GCGGCTACAA TCGTCACAGA AACTTCGCGT CTCCGTTGGA AGACGAAAGA

1861 AACCACCAAA TCGTGGTGTT CATGGACACA GTGGAGTTCT TGAAACCCAA GTATTGCTTG

1921 ATGGAAAACG TGGTCGACAT CATAAGGTTC GACAAGGGCT CTCTGGGTCG ATACGCTCTA

1981 AGCCGTCTGG TGCTCATGCG ATACCAAGCG AGGCTCGGCA CCGTCGCCTC CGGCTGCTAC

2041 GGCCTCCCGC AGTTCCGCCT GCGCGTCTTC ATCTGGGGGG CGCACCCGGG CGAGACCCTG

2101 CCGCAGTTCC CGCTCCCCAC GCACGATGTC GTCGCCAGGT ACTGGCCTCC CATCGAGTTT

2161 GAGCGGAACA TCGTGGCGTA CGACGAGGGC CAGCCCCGCG CCCTGCACGG CGCCATCGTC

2221 CTCGGGGACG CCATCTCCGA TCTCCCGCCC GTCACCAACG ACGAGGACCG GGAGGAGATG

2281 GCGTACGGGA AGCCCCCGGA GACGGAGTTC CAGAGGCGCA TCCGGACGGG GGCCGGGGAC

2341 GTCCTCTACG ACCACCGGCC GCTGAAGCTG TCGGAGCACG ACTACATGCG CGTGTGCCGA

2401 GTCCCGCACA GGAAGGGGGC CAACTTCAGA GACCTCGAGG GGGTGGTGAT CGGAGAGGAC

2461 AACGTGGCGC GGCGCGATCC GACGAGGGAG GCGGTGATGC TGCCGACGGG GAGGCCGCTG

2521 ATCCCGGAGT GCGTGTTCAA GTTGGAGCTG GGGAAGTCGA GAAGGCCTTA TGGGAGAGTA

2581 TGGTGGGATG AGACGGTGTC GACGGTGGTG ACGTTTCCTC ATATTCGAGC AATGGCGATA

2641 CTGCATCCGG AGCAGGATAG GAGGCTGAGC ATAAGGGAAT GCGCGAGGCT GCAGGGATTC

2701 CCGGACTACT ACAGATTCTG CGGCACTATA AAAGAGCGGT ACAGCCAGGT AGGGAACGCC

2761 GTCTCAATTC CGGTGGCGCG GGCCTTGGGC TACGCGCTCG GGATAGCCTA CCGGAAGCTC

2821 GCCGGAGAAG ACCCGCTCAT GACGCTGCCG CCGGACTTCT CTTCTTCTAC TTGA

>SmCMT2a protein sequence

1 MKEQQKLPDQ LPLRKSSSIS SSPSARLCNG AKLEPEARPV DALALSLYVA EAEQQQQPLS

61 ICYPSHEEEE EDNSVRRSPR FTSNKYKYLS PERSRSQKHT ALVVSKLSMS PRIFFSSSAP

121 ALTLTDGGNV EAGPKKRRRL LALPGPAGSG VCLSERCLRS RTVLMRVAES PEASGAKRLR

181 SVGGSPQIEM LALPQATPIS SKNRTRPSAS LNKECSVTPE KESSLAGVRR NGESAVKSKK

241 KEKSILCCFI GEPIPEEEAR ERWQWRYDLK SRIKGKNWKI NAEEDEIILN VASHYAQAKV

301 GNFVLNIGDC AFVKGEGNKK HIGRILEFFK TTEGEDYFRV QWFFRAEDTV LKDTASSHDK

361 RRIFYSILMN DNVLDCIISK VKIVNVPPMP CLKRNRVQSA AFYYDMEYSV QYSTFRTMVT

421 EKSMGNESFL SNSKHPDDGP ITLTPLELLP GYDSPKQELT LLDLYSGCGG MSTGLCLGAK

481 LSSVNLMTKW AVDYNSSACE SLRLNHPETN VRNESAEDFL DLLKQWESLC KRYVSDIERT

541 LKCELEELPE AEAEDCLIPS DTEESSGEYE RIEDFVRRGF NAKILPLPGD VGVICGGPPC

601 QGISGYNRHR NFASPLEDER NHQIVVFMDT VEFLKPKYCL MENVVDIIRF DKGSLGRYAL

661 SRLVLMRYQA RLGTVASGCY GLPQFRLRVF IWGAHPGETL PQFPLPTHDV VARYWPPIEF

721 ERNIVAYDEG QPRALHGAIV LGDAISDLPP VTNDEDREEM AYGKPPETEF QRRIRTGAGD

781 VLYDHRPLKL SEHDYMRVCR VPHRKGANFR DLEGVVIGED NVARRDPTRE AVMLPTGRPL

841 IPECVFKLEL GKSRRPYGRV WWDETVSTVV TFPHIRAMAI LHPEQDRRLS IRECARLQGF

901 PDYYRFCGTI KERYSQVGNA VSIPVARALG YALGIAYRKL AGEDPLMTLP PDFSSST

>SmCMT2b genomic sequence

1 ATGGCGGATT CTGCCCAACT CGACTCGGCT GATCCGCCAC TAGCTCCGCC TTCACCACCG

61 CCTTCGCTTC CGCTGCAGCT GAATGGAAAT TCTGATCCTG CGACTCCACT TCCAAGCAGT

121 ACCGATGGAG GAAGTGATAT TTTGGATAAT TATCCTCAAT CCAATGGCGT CGCGATGAAA

181 AAATCAGCTA ATCGGAGAGT GGAGACCCTG AGGACCTCAG AGGATCACTT TCGCCGGCGT

241 TCGCCAAGAC TGACTGGAAA TGTTGCAGGT GAGCGCTCCG TTAGAGAGGT GAGTTCTCTC

301 AAATTGGATG GTCCGGATGA GATATATCCG CCGCTTAAGA AGCCTAAAGG TGGAAAGCAG

361 GTATCTTTCT TCATCGGCGG GCCGGTACCT GATGATGAGG CTCGCCGCCG GTGGACATGG

421 CGGTATGAGG GAGCTGAGGT AATGTGGTCT GACTTTTTAG TTGAAATATT TCTACATTAT

481 CATGAATTAT TGTGAAATGT AACTGAACTG GATTTTTATT TGGTTATGAG AAATTTGATG

541 ACATCGTTGT GGCAAATTTG GTAGGTGTAG AAGCATGATA TACGTAGATC TTGTAGAAGA

601 AGGACATTAG TTCGTTAATT ATACAAATGC AATGAAAACC TGCTCATTCT CACATTGGAG

661 CCACGAAGTC ATTCGTTGTA TGTGAACTTT TTTTTAAAAT GTTAAGATTT CACTGCAACA

721 CAATAAGATG TATTTCAGAA CATGTCATTG ATGAAACTAA TTTGCAGGAA ATTTTGTTTT

781 TAAGCAATAT TATCTTTTAA ATTTCCCATG GATTGTTTGT ATTTCTAATG CTCATTAATT

841 TTGTTAGGGA TATTGTGAAC GCATTTGAAC TCCAATGCAT GATGAAATCT TATAAACACG

901 AAAGTTGGTT GGCTCAAAAG TTTAAATGAT TGGGACTAGT CCTTCATCTA GTATTTGCAA

961 ATATCTCTTT CCTCTAAGAG AAGAACTCAA AAGAAGAAAC GGCATTCACT TAAATATATT

1021 ACCTGAATAA GAGCCAACAA ATCACACATT TTTTGTTACA AAACTTCTAA ACAATAATAG

1081 ACAGTTCTGA AACCATGTAT GTGTTCATCT TGGTCATGTT TGGACTTCAA TTTGGTGGTT

1141 TTTGAATATT CACTTTGTTC ATGTATATTA GAGGACTTGA TTTTTGCTAT TGTTGTTGCA

1201 GGGAAAACAA AATAATGAAC TATCACCAAA ACATAAGTAA GTGCTATACT GCTATTAAGC

1261 TTCATTTATA GATTTGGGAT TTTAGTGATG TACTTTGTTG TTTTTCATCC ACATTGATGA

1321 TGTTGCAACT TTTGTAGGGT TGTTGTTGAT GATGATGATG ATGGTGATAA ACTTGTTTCA

1381 AATGTAAAGT GTCACTATTC TCAAGCTGAA ATATCGAAGA TTGTATTTGA TCTCGGTGAT

1441 TGTGCCTATG TCAAAGTAAG TTTGCTCTTA ATATTTTTTA TTGCCACATA TTTGAGATGT

1501 CACTGACAAA CTTGTTGTAT TGCAAATATC AATATGTGGC AACCAATTTT AATGCATTAC

1561 ATTCATATTA ATGTAAATAA GTTAGTACAG CTTAGTGAAT GCTTTTGTCA AAGTTGGTAA

1621 GAAAACCCAA GATGATATGT GTTTATATGA ATCGAGAAAT ATTGTGAATT CTTTGTATAA

1681 GTGTATATGT CTGAAGTGAC ATATAACTTG TTCACTTGAA CTGATCTTAG CATTGAGACT

1741 GGCATTCACC TAAATGAATA AATTAGTTTT AAAACTAAGA GGCAACCCAT AGATATTAAT

1801 AAGTTGCCCG AGGCTACTTA ACCAGTTTGA GATAAGAGAT GTTACTAAGG GCGCACACAC

1861 TTGGTGTGCC TCGAAACTTA GGTGAGGCTT GATGGCCTCA AAGTTGCATA ATCATAAATG

1921 TTTTCTCTTT TTCCCCTATT CCTTTTTATC AAATATATTG TGTCTAAATT TAAACATTTC

1981 ACATAATATT GAGACTAAAA AAACTCCAAA ATCTCTATCC ACTGTGGTTT TGTCTTTCTT

2041 GTAAAGTTGG TCAATTAGCT CCTCTTTTCA TTGCTGGTGT AGCTAGTAGT TACATTTTCC

2101 TATTCTCAAA TCTCACTCAT CTCTTTTCAA TGATTATAGG GCCCAAAGGG AAGACCTAAT

2161 TATGTTGGCA AAATTCTAGA ATTTTTTGAG ACAATGGATG GTGAAAACTA CTTTAGTGTT

2221 CAATGGTTTT TTCGAGCAGA AGATACGGTG ACAAAATAAT ACTTCTATCT TTTTTATACT

2281 TACTGGTGCC TTCTTTATTC CCTCTTTCTC TACCTTTACT AATTAGTTTA CCTTGCCAGG

2341 TTATAAAGAA TGATGGAAGG TCTCATGACA AGAAACGGTT ATTCTACTCT ACTCTCGTGA

2401 ACGACAACTT ATTAGACTGC ATAGTTTCAA AAGTTAAAGT TGTTCAAATA AAACCAAATG

2461 TATGTTTGAA ATTTGTACGC TCAACTTACA ATTATTTTCT ACTTATCTGT ATGTTAGTGA

2521 CCTCAATACT GTATTTTAAG GAGGGAGACA TATTTTTGGT CCATCATCTT TTCTCCCAAT

2581 TGAATATTGT TCTAGTTGTT CTTCTAACAA TATTGGAAGG ATAGGTACTA AAGTTTGGTG

2641 AAACTTCACC AATGAAGAGT GATAGAAATT ATTTCTTAAA TTGACTGATT ATATTCTTGT

2701 TTACATATGT TATTGACATT CACTTTCTTG TAGGTTACTT TGAAAGATAA TACAATACCA

2761 CCTTGCGACT ATTATTTTGA TATGAAATAC GATGTCGACT ACTCAACTTT CAGCTCAATT

2821 TTAACAGGTA CCTTGTACTA TTTTGCTTTT GAAAATTTGA GAAAGGCATG ATTCATGCTT

2881 TTGTATAGCT TGATATGCAT ACTTCTCTGC AAGTAATTTC TGTATGAAGT TCTGGTTTGA

2941 TTGCCACTAC CTTCAAGACT ACAATTGCGT GATGTCACCT TTGAAAAGTT TATAGTGCAT

3001 TTCCGTACTC TCTTATTAGT TAGCACATTA AGTTCTCCAA ATCCAATTTA AAACCACCTA

3061 AGAAACACAT TATGGGCACA TGCTCAAAGT TAGGCACACT GGATTTGGTT CAATTGGCTT

3121 TGTGTAGAAA CATTAATTGC AAAATGCTTT TAAAGAGTCT TGTGCAAATA GATTTTAATA

3181 GCATATTTGA CTTACTTTCC GTGTGGACGT AACTCAAAGT TGCTATGAAC TGCTAAAATA

3241 GTGATTTATA TCGTGTGATT AATGTTGTTA AACTAAAATA GAAGATATAC AATCTAGATA

3301 CAGTAGTTCA TTCGTCGATG ATGATTAATT AAGATACTCC ATCCGTTCAC GATAAGTGTG

3361 GTGTGGATGG AAGGGCACGG GTTTTAAGAA AATATTGGGA GATGTGTGTA AAGTATAAAA

3421 AGAGACCCAA CAAGTTGATT TGTTAGGTAA TTTATAAATG GTTTGTGAGA TGAGTTTTTT

3481 ATAAACATTG TGTGTGAATG GGGGTAAAAA TATAAGGGTA TATCAGTATA TGTCCTAAAG

3541 TGGAAAGATT ACACTTATTT TGGACGGACG AAAATGGCAA AAAGACCACA CTTATCGGGA

3601 ACGGAGGGAG TATAATCCAG TAACAATTGA GTTGGTAGAC ATACCTAGCT ATGAGAAATC

3661 AACCATTTTT TTTCTACCTT TCTTGTTTAA CTTCATCCTT CAAAATCTTA ATCTGCTGTA

3721 AAGCAATCAA TGTAATCACC TATTTTAGCC CCCATTTTCG CTGATCTGCA ACTAACACTC

3781 TATGTGACCA CCTACATACC GTTTTATTCA TGCAGATGGT GACAGTGAGT TATTTCCTCA

3841 TCCTTTGATG ATGTCATCCA AAGGCAAATA CGCAACCTCT AAAATTTCCA CCAAGTGTTC

3901 TTTGGATGAA AATTGTAAAT CTGAGCTAGC TTTACTGGAT ATGTATTCTG GCTGTGGAGG

3961 AATGTCCACT GGATTGTGTA TTGGTGCTAA AGCGTGTGGC TTGGATCTCG TGGCGGTAGT

4021 ATAGCAAGAC AGTTTAAACA TTTAGAAATA TGGTTATGTC AAGAAATAGT ATTTGTGTTT

4081 AATAGTCATG ACTAACTTCC ATACATAACA GAGATGGGCT GTTGACATTG ATGAAGCAGC

4141 ATGTGAGAGC TTGAAGCTGA ACCATCCAGA GACACAAGTT GTGTATTATA TTTTCTTTCT

4201 CTTCGCATTT TGAAGTCCCT AATTCATTAG AAAATTTTTA GTATTCAAAT GTCTTGTTGA

4261 ACAGATAAGG AATGAAGCTG CAGAAGACTT TCTTGATTTG TTGAAGGAAT GGGATTGCAT

4321 GTGCAGAAAA TACGGGAAGT ATGACCAGAA AGAACTCAGG TTAAAGACGG TTAGGGAAGC

4381 TGATGAAGTT AAACTATCAA AAAAGAATCA AGAAAGTTCA TGTGAATATG AAGTGGCAAG

4441 GCTGATAGAT ATATGTTATG GTGATCCTTC TAATTCAGGG AAGCGAGGGC TTAAATTTAA

4501 GGTGCAATTT TCTTATTCAT GCTTGACTAA TATGGTAAAT AGTCACTCTA ACATGTAGAT

4561 AGAAGAAACC CTAAGTATCT TATTGCTCGG AATAACTTCC TTAGAAGATG TTTCCCTTGA

4621 TTTATATGTA ACAAAAAATT ATCATTGCAG AATAGCTAAT ATAAAAAGCC AAGAAGGGAA

4681 AGTAGTATTT TCAGTTCAAT TGGCTTCTTT GTTTTCCATG TAGAAAATTA GTAGACCAAG

4741 TAATATAGAC ATCACAACAA GACTTTTGGT AGATGGATGT TACAGACTTA CAGTTTACTT

4801 GTTTTTTTTT ACTTAAAGTG ACAGGTAGTA AAGATATAAA ATAGTCATGT AAAATGAGCC

4861 TGGAACTTTA TTGTCTGTGT TGGCAAATGA GTTCATCTTC ATTGATGTAT AATGTGCATC

4921 ATCTAAATTG CTACTTGGTT AAATACGTTG ATGTATGCCT TTAGCCATTT AATGTCATGT

4981 TTATATTCTT TTAGGTGCGC TGGGTTGGAT ATGGCCCTAG TGATGATACA TGGGAGCCAA

5041 TTGAGAACTT GGGGTATGTT TCCATCATAT ATAATCAGCA GTTACAAATT ATCAAATGAA

5101 GTTATTTCAC TGATAACTTC TGACAAGCTT ATTCTGCTCT TCTCTACAGA AAATGCCCAG

5161 AGCACATAAG AGATTTTGTA CAAAAGGGAA TAAAAGCACA AATACTGCCT CGTCCGGTAT

5221 GTTTTGGTTT GTTTGTGAAG GTTACACATG CGCTATAATG GGGGAAAAAT CAGATCAGTT

5281 TTCTGATATT TGATAAAATT TATTCGATAG TTTGCTACAG AAGATATTTT GGAATATTTT

5341 TTTTAAATAA ACACCAAAAA CATGTTTTAG AAAGTAGATA ATTGCATGGT TATAAAAGTC

5401 TCAAGTAAAA AACACATCAC ATGTTTTCCT ATTGCACAAA TGGATAAGTT CATAGTGAGC

5461 TACAATACAT GTTTGTCTCA TTGAAGACTG TTTATCTGAA ACTAATCTTG TGGAAATATT

5521 TTGTGTCATG CAATCATTGT TTACTAAAAA AATACTATCA CCATCTAATA TTCCTCCATA

5581 TGCCATCAAC TTATCACAGG GAGATGTTGA TGTAATTTGT GGTGGTCCTC CATGTCAAGG

5641 AATTAGTGGC TACAACCGGT TCCGGAATTT CGATTCTCCT CTTGATGATG AAAGAAATCG

5701 CCAAATATTG ATTTTCATGG ATATTATTGA ATTCTTAAAA CCAAAGTTTG TTTTAATGGA

5761 AAATGTAATT GACATCTTGA GGTTTGCCAA CGGCTGTCTG GCAAGATATG CTATAAGCCG

5821 GTTGGTTAGT ATGCATTACC AAGCAAGGGT TGGGATAATG GCTGCTGGTT GCTATGGGCT

5881 TCCTCAATTC CGCTTACGAG TTTTTTTATG GGGTGCTCAG CCTCTTGAGG CAAGTATTCT

5941 CAATCCTTAT CTTGGCACTT CGGATATCCC TTTGCGACGA AATTTGGTAC TTTCCATTTT

6001 GTAACTAGTA TAGCTTGGTT ATTTGGTATA ATTTTGATTT TTACCAAACC ACTAGTATCT

6061 CTTAGTTTTG TTTAGTTCCT TCATTTGCCA TTTTTCGCAT CTTTAGTTCG TGTCATCCTT

6121 TTCTTTCTTG TCATGGAGTT AAATTAAAAG AGATGTGCAC CAATTGCAGA TTTTCCTGTT

6181 CGTGGTGTAT TAACATATAT TGAATATGTT AAAGCATGTT TTTCTGTATA TATCTACATA

6241 TTCAACCTTC CTCCTTATCG GCGGCCATTA TATTTATCAT GAATTCTCTC ATGACGAAAG

6301 GCTTTACATT TTATCTAATG TTGGCCTCAA GTTTGTTATC ATTCCTCTTG GTAGCCACTA

6361 GGGCAAACAG TCAAAATGGA AGTTTCATTT CCCTTGTAAA TTTACTAAAC CTGCAAGTGA

6421 CTGTCATTTT ATTGGCAGGT GCTACCCCAG TTTCCTCTTC CATCACATGA CGTGGTAATC

6481 CAGTATGGAT TTCCTAGTGA TTTTGAGGTA CTGAACGTTT ATCACATGAC ATTACGATGT

6541 AGTAACCCAA TAAGTATCTA CTTATAATCT GATTTCATCT TGCAGCGTAA TGTTGTTGCT

6601 TATGATGAAG GAAAATTCCG TGATCTAGAA AAAAAAGTTC TTCTCAGTGA TGCTCTTTCT

6661 GATCTTCCTC CTGTACGTTT AATTTGTTGC ACACTTTGAT TAATTCTGAA ATTGTAAAGT

6721 TTGACTTGTT TCATTTCTAT ATTCAGGTTG CAAATAATGA GAAACGTGAC AGCATTCCAC

6781 TCAAAACAGT ACCTGAAGCT GAGTTTCAAA AGTATATTAG AGCAGCTAAA TGTGGTAGGT

6841 CATTAATGGT CTATTTTTCA GTTCACTCTT ACCTATAGCT ACTCTGCTGC CTATCACTTT

6901 CACTATCCCA CAAGAACCTG ACTTGATCCA AGAACATTTG AACTAATTTT TAATGGGTAT

6961 TTCAAATGAA GCTAACTACT TAGAACATCC TCTGAAATGG CTATACAGAA GCTTGCCTCG

7021 TATGAGGTTT TTAATAAACA AATCCTCTGC AAGTAATTGG CTGGTGAGCT ATTGCCATGC

7081 CAAAAGCCTT ATCGCTCACA TGATCACATC TATACTTAAT CGACGGAGGG AATTTTCATC

7141 TTAGGATCTA AAATGCAAAG TATACCTCCT AGTTTGCATA AGTAAATACT CAAGACATCA

7201 GTATAACTCA TAGCTTTGCA AAGTTTACCT CCTAATTTGC AAAAGTAAAT ACTCAAGACA

7261 TCAGTATCAT ACGTGAAAAA ACTTGGTCAA ATATTCTGTC ATGTCAGCTT ACTGAATCCC

7321 AGAGACCGAA AATAGCTGCA AGGAAATGAG ATTCTAACTT CTATTGGAGT TTTATCTGCA

7381 AAAAGGAATA TATACTAATA CTATAAATTA AGTGCTTCCT TATCTAAAAA AATAAAAAAA

7441 TAAAAAAAAN ATCAATGCTT CCTTATATGC ATGAGACCAT CCAAATAGGG TTGGAACAAA

7501 AAATTTGGGC TAACTTTGTA GTTCCTTTAT CTCTACTCTC TAGATATGAC TGCTTCACCT

7561 TCCAGTGTCA CAACTACACA TAAGAAACCT GTTTTGTACG ATCACCGGCC TTATCCTATG

7621 AATGAGGATG ATTACCTGCG AGTCTGTAAA ATACCAAAAA GAAAGGTTAG TTCAGTGGGA

7681 TTCTTTGCCA CACATTCAGC ATCCTGGTTC TGATTCGGTA CTTCACTCAA CAGGGAGCAA

7741 ATTTTAGAGA CCTCCCAGGA ATTGTTATTG GAGCTGGCAA TGTGATTCGC CGAGCGAAGG

7801 AGCAAGACTT GATGCCATCT GGAAAGCCAT GGGTAATACT TATAAATTAG TCTAGCTTTG

7861 GCTCCAAACT CCTATATATC TAAATTTACT TCAAGTGAAT CTTTGCTCTT ATGTGACTGT

7921 TAATGTTTGT GAGTTTGCCA TCAATCCATG ATCTCTGCAT TTAGCTTTGC TTTTGAATGA

7981 CTTCACTTTA TTATAAAATT AGCATGAGGG TAAATGAATA TTAGACGAGT GTGTTGCTTT

8041 TTTAATGCGC TCTTGCTATT TCAATAATCA GGTGCCTGAC TATTGCCTTA GTTACCGCGA

8101 TGGAAGGTCC CATAAGTGAG TTACTTGGAA ATGAGCAGTA TAAATCAATT CAGTTTTTTT

8161 CATCCTTCCT CTTTTTCGCT TACTAACAAG ATCCTATCAA TAATCGAATA CTGCAGACCA

8221 TTCGGACGAC TGTGGTGGGA TGAGACCGTA CCAACTGTTT TCTGTTTTCC AGATCATCAC

8281 TCGCGGGTCT GCATCTTTTA CCTCATTTTC TTAAACTGGT TCGAAAAGTT TAGCCAATTA

8341 TTCATTTATC TTTCTTTTTT TTTCCTTCCT TCCGTTGAAG GCAATTTTGC ACCCGGAACA

8401 AGACAGGATC CTCACTTTAC GTGAATGTGC TAGACTACAA GGCTTCCCCG ACTACTACAT

8461 GTTCTCTGGA AGCTTGAAAC AGAGGCAAGT TTTCATGTCG TTAACTTGCT TGTTTAGGAT

8521 GTTGTGTACG ATACTGCAAT TTTCAATCAA ATCTCTAATA TTAGAGCATC GAAACTGATC

8581 ATACTACTTA AAAGACTCAT ACTTGACACA TAAGCACATA TTAATCTTTA TCCTTCTTCA

8641 TCTACAACAG ATATAGTCAG GTCGGAAATG CAGTAGCTGT TTCTGTTGGA CGCGCTCTAG

8701 GATACTCGTT AGGTATGGCA GTGCAGAGGC TGAGCGGAGA CGAACATCTT CTCACTCTTC

8761 CACCCAAGTT CTCCCATTCC ACAACTGTTG AGTTGCTATC TTTGCTCAAC CAGTGA

>SmCMT2b ORF sequence

1 ATGGCGGATT CTGCCCAACT CGACTCGGCT GATCCGCCAC TAGCTCCGCC TTCACCACCG

61 CCTTCGCTTC CGCTGCAGCT GAATGGAAAT TCTGATCCTG CGACTCCACT TCCAAGCAGT

121 ACCGATGGAG GAAGTGATAT TTTGGATAAT TATCCTCAAT CCAATGGCGT CGCGATGAAA

181 AAATCAGCTA ATCGGAGAGT GGAGACCCTG AGGACCTCAG AGGATCACTT TCGCCGGCGT

241 TCGCCAAGAC TGACTGGAAA TGTTGCAGGT GAGCGCTCCG TTAGAGAGGT GAGTTCTCTC

301 AAATTGGATG GTCCGGATGA GATATATCCG CCGCTTAAGA AGCCTAAAGG TGGAAAGCAG

361 GTATCTTTCT TCATCGGCGG GCCGGTACCT GATGATGAGG CTCGCCGCCG GTGGACATGG

421 CGGTATGAGG GAGCTGAGGG AAAACAAAAT AATGAACTAT CACCAAAACA TAAGGTTGTT

481 GTTGATGATG ATGATGATGG TGATAAACTT GTTTCAAATG TAAAGTGTCA CTATTCTCAA

541 GCTGAAATAT CGAAGATTGT ATTTGATCTC GGTGATTGTG CCTATGTCAA AGGCCCAAAG

601 GGAAGACCTA ATTATGTTGG CAAAATTCTA GAATTTTTTG AGACAATGGA TGGTGAAAAC

661 TACTTTAGTG TTCAATGGTT TTTTCGAGCA GAAGATACGG TTATAAAGAA TGATGGAAGG

721 TCTCATGACA AGAAACGGTT ATTCTACTCT ACTCTCGTGA ACGACAACTT ATTAGACTGC

781 ATAGTTTCAA AAGTTAAAGT TGTTCAAATA AAACCAAATG TATGTTTGAA ATTTGTTACT

841 TTGAAAGATA ATACAATACC ACCTTGCGAC TATTATTTTG ATATGAAATA CGATGTCGAC

901 TACTCAACTT TCAGCTCAAT TTTAACAGAT GGTGACAGTG AGTTATTTCC TCATCCTTTG

961 ATGATGTCAT CCAAAGGCAA ATACGCAACC TCTAAAATTT CCACCAAGTG TTCTTTGGAT

1021 GAAAATTGTA AATCTGAGCT AGCTTTACTG GATATGTATT CTGGCTGTGG AGGAATGTCC

1081 ACTGGATTGT GTATTGGTGC TAAAGCGTGT GGCTTGGATC TCGTGGCGAG ATGGGCTGTT

1141 GACATTGATG AAGCAGCATG TGAGAGCTTG AAGCTGAACC ATCCAGAGAC ACAAATAAGG

1201 AATGAAGCTG CAGAAGACTT TCTTGATTTG TTGAAGGAAT GGGATTGCAT GTGCAGAAAA

1261 TACGGGAAGT ATGACCAGAA AGAACTCAGG TTAAAGACGG TTAGGGAAGC TGATGAAGTT

1321 AAACTATCAA AAAAGAATCA AGAAAGTTCA TGTGAATATG AAGTGGCAAG GCTGATAGAT

1381 ATATGTTATG GTGATCCTTC TAATTCAGGG AAGCGAGGGC TTAAATTTAA GGTGCGCTGG

1441 GTTGGATATG GCCCTAGTGA TGATACATGG GAGCCAATTG AGAACTTGGG AAAATGCCCA

1501 GAGCACATAA GAGATTTTGT ACAAAAGGGA ATAAAAGCAC AAATACTGCC TCGTCCGGGA

1561 GATGTTGATG TAATTTGTGG TGGTCCTCCA TGTCAAGGAA TTAGTGGCTA CAACCGGTTC

1621 CGGAATTTCG ATTCTCCTCT TGATGATGAA AGAAATCGCC AAATATTGAT TTTCATGGAT

1681 ATTATTGAAT TCTTAAAACC AAAGTTTGTT TTAATGGAAA ATGTAATTGA CATCTTGAGG

1741 TTTGCCAACG GCTGTCTGGC AAGATATGCT ATAAGCCGGT TGGTTAGTAT GCATTACCAA

1801 GCAAGGGTTG GGATAATGGC TGCTGGTTGC TATGGGCTTC CTCAATTCCG CTTACGAGTT

1861 TTTTTATGGG GTGCTCAGCC TCTTGAGGCA AGTATTCTCA ATCCTTATCT TGGCACTTCG

1921 GATATCCCTT TGCGACGAAA TTTGGTGCTA CCCCAGTTTC CTCTTCCATC ACATGACGTG

1981 GTAATCCAGT ATGGATTTCC TAGTGATTTT GAGCGTAATG TTGTTGCTTA TGATGAAGGA

2041 AAATTCCGTG ATCTAGAAAA AAAAGTTCTT CTCAGTGATG CTCTTTCTGA TCTTCCTCCT

2101 GTTGCAAATA ATGAGAAACG TGACAGCATT CCACTCAAAA CAGTACCTGA AGCTGAGTTT

2161 CAAAAGTATA TTAGAGCAGC TAAATGTGAT ATGACTGCTT CACCTTCCAG TGTCACAACT

2221 ACACATAAGA AACCTGTTTT GTACGATCAC CGGCCTTATC CTATGAATGA GGATGATTAC

2281 CTGCGAGTCT GTAAAATACC AAAAAGAAAG GGAGCAAATT TTAGAGACCT CCCAGGAATT

2341 GTTATTGGAG CTGGCAATGT GATTCGCCGA GCGAAGGAGC AAGACTTGAT GCCATCTGGA

2401 AAGCCATGGG TGCCTGACTA TTGCCTTAGT TACCGCGATG GAAGGTCCCA TAAACCATTC

2461 GGACGACTGT GGTGGGATGA GACCGTACCA ACTGTTTTCT GTTTTCCAGA TCATCACTCG

2521 CGGGCAATTT TGCACCCGGA ACAAGACAGG ATCCTCACTT TACGTGAATG TGCTAGACTA

2581 CAAGGCTTCC CCGACTACTA CATGTTCTCT GGAAGCTTGA AACAGAGGCA AGTTTTCATG

2641 TCATATAGTC AGGTCGGAAA TGCAGTAGCT GTTTCTGTTG GACGCGCTCT AGGATACTCG

2701 TTAGGTATGG CAGTGCAGAG GCTGAGCGGA GACGAACATC TTCTCACTCT TCCACCCAAG

2761 TTCTCCCATT CCACAACTGT TGAGTTGCTA TCTTTGCTCA ACCAGTGA

>SmCMT2b protein sequence

1 MADSAQLDSA DPPLAPPSPP PSLPLQLNGN SDPATPLPSS TDGGSDILDN YPQSNGVAMK

61 KSANRRVETL RTSEDHFRRR SPRLTGNVAG ERSVREVSSL KLDGPDEIYP PLKKPKGGKQ

121 VSFFIGGPVP DDEARRRWTW RYEGAEGKQN NELSPKHKVV VDDDDDGDKL VSNVKCHYSQ

181 AEISKIVFDL GDCAYVKGPK GRPNYVGKIL EFFETMDGEN YFSVQWFFRA EDTVIKNDGR

241 SHDKKRLFYS TLVNDNLLDC IVSKVKVVQI KPNVCLKFVT LKDNTIPPCD YYFDMKYDVD

301 YSTFSSILTD GDSELFPHPL MMSSKGKYAT SKISTKCSLD ENCKSELALL DMYSGCGGMS

361 TGLCIGAKAC GLDLVARWAV DIDEAACESL KLNHPETQIR NEAAEDFLDL LKEWDCMCRK

421 YGKYDQKELR LKTVREADEV KLSKKNQESS CEYEVARLID ICYGDPSNSG KRGLKFKVRW

481 VGYGPSDDTW EPIENLGKCP EHIRDFVQKG IKAQILPRPG DVDVICGGPP CQGISGYNRF

541 RNFDSPLDDE RNRQILIFMD IIEFLKPKFV LMENVIDILR FANGCLARYA ISRLVSMHYQ

601 ARVGIMAAGC YGLPQFRLRV FLWGAQPLEA SILNPYLGTS DIPLRRNLVL PQFPLPSHDV

661 VIQYGFPSDF ERNVVAYDEG KFRDLEKKVL LSDALSDLPP VANNEKRDSI PLKTVPEAEF

721 QKYIRAAKCD MTASPSSVTT THKKPVLYDH RPYPMNEDDY LRVCKIPKRK GANFRDLPGI

781 VIGAGNVIRR AKEQDLMPSG KPWVPDYCLS YRDGRSHKPF GRLWWDETVP TVFCFPDHHS

841 RAILHPEQDR ILTLRECARL QGFPDYYMFS GSLKQRQVFM SYSQVGNAVA VSVGRALGYS

901 LGMAVQRLSG DEHLLTLPPK FSHSTTVELL SLLNQ

>SmCMT3 genomic sequence

1 ATGGCGAATA AGAGGAAGTC CGAATCGGCG ACGAAGGATC CGGCCGCAGC CACGGCGGAA

61 GGAAGCCGGC GGTCCAAGAA GCCAGCCTCT GCTGAATCAA CTGGGGCGGT GGACGAGGAG

121 ACAAGGATTC CAACTGTCAT AGACGACGGG AGCGAGGATG CAGGTGAGGC CCTGTTTTTA

181 GGTGAACCGG TCGCCGGCGA GGAAGCTCGG AAACGGTGGC CGCATCGATA TCAGAAAGAT

241 AATCAGTCAA CGGTACATAG CTTCTGTTTA TATGGCATAT GCTGCTAATT GTGTTAGGTT

301 ATGCGTATAT GGTTTTTTTG GAAGGCTTAT GCGTATATGT TTATGATTTG TGATTGTGTA

361 TATGTGTTTG TGGCACATTT AAAACATGCG GAGAATATAT GGATTTTGTT TATATATAGG

421 GTTCGTTCAG TTGAGCTGCC ATGGAATTGG GATTGGTTTT AAATTCAAGA TATTATAATT

481 GCGGATACTT CTTACTGAAT ATTCACTAAT TAGTAATTAC TAATGCATTG TATTTAGGTT

541 GTCACTAATG AAAAAGTCGA GATTGTTGTG AAAAATGTGA CAAACATTGT GTGTTGGATG

601 ATCCTCTATC CCATAATATT ATTATGGTAT CATTCTTAGT TTTACTATTT TAGTAATGAT

661 TTATAATTAA AAGTTGAATT TTGAAAAGTA TTAATTGACT CTAATAATTA ATTACTAATT

721 CAAATAGATA AGTGATTCCT AATTAATTAT TTTAAATTAT ATTAAAACTT AATGAATTAA

781 ACTATAACAA ATCGCAAATG AAGATATGTT AAGACACAAT AAATTATGGA GAAAAGGATT

841 AATGTATATG TGGTATTTAA TGTAACATGC TTAAATTAAC TGTTTCGACC AGATTGGCGT

901 TGCCAAAAGG TTAATTGTAT TCAAAGATTT TGATTTTGGA CGGAATCGTA AATATACTCC

961 GGCATTAAGT TTCATTAACT ACGTTTTTAA TTGTGATGCC TCGGTATTAC CAAGGAAAAG

1021 GAAAGTTGGA CTGCATGTTT TAAAAGACGG AGTGCATGCC ATTGGTTAGT GAAATAGCAG

1081 ATAGTAACAT TTTTTTTTAT TTTGTTCGAA TTGTATATAG TACTATATTT TTAAATTATA

1141 AATAAATACG CATTTTTCTG TCTCATTAAA GATGATTTGT TTTTCATCTT AGATCATCTC

1201 ACAAAAGATA TCTTATTTTC AAAAAAGATA ATTTAATTTA CATTTTTTTT CCATTAATCT

1261 TAGTGTTAAA AAAGTAAATT ATTTTTTAAT TGTAAATAAA TATGCATTCC GTCCCATTAA

1321 TGATAATTTG TTTTTTATTT TAGATCATCT CTTAAAAAAT ATCTTATTTT CATAAAAAAT

1381 AATTTAATTT ATATTTTTTC ATTAATCTTA GTGTAAAAAA ATAAATTAAT TTTTATGAGA

1441 CCAAGCGAGC ATAACTTAAG ATATTGATCC CCTAATTCAC GGCAAAAGTT TACTTTTGTA

1501 TTATTTTTGA AAATCCTTTG AAAGTTAATA ATTCATTCTT TTCTTTTGAT TGTGGTTGGT

1561 TATGTTTTTG CGAGAATATA TAAAAGTATC AACTTCTATA AAAGTGGGTA CTCAAAATAT

1621 TCATTTTTAA GAGATGCACT TTTTACCATT TATAATAAAA AACTCATGCT CCTTCCGTCC

1681 GCTAAGATTA TGGCAATTTG CTTGGGTACG AGATTTAATA AAATTGGTGA TGATTTTGAT

1741 GTAGTGGAGA AAGGGTCCCA CCACTTTATG AGATGAGTGG TTGAGATTGA ATTTTGAGTG

1801 ATTTTTTTGT AAATAAATAG TGTTAATAAG AATAAAATAT TAAAGAGAAT GGTGGGACCA

1861 TTGCTATAAC AAGAAAGTGA CATAATTTTG GCGGACGTCC AATATATTAA TTGTGACATA

1921 ATCTTGGCGG ACGGAGGGAA TACTATTTAC TTCTCCTTTG TGCTCGTATC ATCACTTCTC

1981 TTACTCCTCA TTTCAGTTTC GCCAGAGACT ACATTTTCGT ACCGCTTGTG CTTTTTAATT

2041 TCATAGTAAC TGTTTACATT GTGTATTCGG GGAGAGAAGA AGGATGGTTT CTTTGTCTTA

2101 ACGCACTTTC ACCTATTTTT CTTGGCAAGA ACTATTATGT GTCATGGATG TCTTGCCTTG

2161 GATTTTGCAG TTGTGATTTT GCAACCCTCA CATTGAGTAA GCTGTATTGA TATTACTATT

2221 CTGGCTTCAT GGATAATCGT AGGAAATCGT CCAAGCGAAA TGCCATTTTC TTCAAGCGAA

2281 GGTTGATGGA CTGGTTTATT GCTTGGAAGA TGATGCCCAT GTCAAGGTTG ATGGACTGGT

2341 TTTTGGTTTC GTCTTTATAG CTCTTTGTCA ATTTTCCTGT CCCCACTTTT TTCACCTGCA

2401 GCACTGTACT ATTGTGGGTT TCTTGATTGT GTAAACGTCA AGCTATTTTA AACTGACTTG

2461 CAAATTTTTT CGGTCGTTTA GGCTGAGGAA GGGAAGGAAA GTTATATTTG CAAGATTGTA

2521 GAATTGTTCC AAGCTGTTGA TGGGACACAA AATTTTTCAG CTCAGTGGTA TTATAGAGCA

2581 GATGATACGG TGAGGTGTTG TGAAATAAGT ACTGTCAGTT GTTTTCTTAG TTAATAGTTC

2641 TGACATAGTT TCTGTTTTCT TTGGTAGGTT ATTAAAACAT GTTCTAATCT TATAGATAAC

2701 AGACAAGTAT TCTTTTCTGA AGTCAAGGAT GATAATCCAC TTGACTGCCT TGTAAAAAAG

2761 CTTCGAATTG TGCTTTTACC TTTGAAGGTA TGTGTGAATC CATTAGAACT GTGTTCTTTA

2821 CTCAGCGGTG ATGTGGAACC TGATTTAGTT TCTTGCATGT TTATAGGTTG AAGATCAGGT

2881 AAAGGAAAAG CTTAGATCAA CACATGACTA TTACTATGAC AAGATGTACT TGCTTCCATA

2941 TACTTCTTTT GTAAACTTGT CATCAGGTGT GTTTATCTTT TTCCTATGTT AAATAGGCAT

3001 TGGTTTGTGA TTGAGGATTT ATATTATTGG AAATTGAGAT AGATTCTGAG TTTCCTTTTT

3061 CTTTCTTTGT ATGCTCTTTA TTTGCAGAGA GTACTACAAA CAGCAATGAA TCTGGTTCTA

3121 CCATCTCTAG TGATGCTGAT GGCAATTCAG TTACACATGT ATTTTCTGAG CTTGAAGGGC

3181 CAAGTGGTGA AAAAAGTCTA TTGGACATGT ACTCTGGATG TGGTGCAATG TCGACTGGGC

3241 TATGCCTTGG TGCTAACAGT AATGGGGTTA AACTTGTTAC GGTAAGTTTG GTCTTTCCTC

3301 ATTTGTTGTG CCACCCTTTT CTTATAGACA AGTTTGCTTT AAGTTGAAAC TGTTCCAGTT

3361 ATCATGGATT GACTTGTTCT CTATGATGCT TCTATTTTAA CAAACAGAAA TGGGCTGTTG

3421 ATCTCAACCC ATATGCTTGT GAAAGCCTGA AACTAAACCA TCCAGAAACA CAGGTAATTA

3481 TCTGTAAGCA TATTAATGAG TTCTATGTTG GACCAACATA TTGATCTTTT ACTTTTACTC

3541 ATCTTGGTAC AATAGGTAAG AAATGAAAAA GCAGATGATT TTTTCCACCT GCTTGTGGAG

3601 TGGGAGAAGC TTTGCACTTC CTACTTGTCC TCAGAAAACA AAGGCACATC CAGTGAGCAT

3661 GTCAAGGAAG AAGTTGAAGA TGATAGTGAG GAAGATGAAA ATGGCGACGA AGATGACTCT

3721 GAAGTGTTCG AAGTTGAAGA GATCTTGGAG GTTTGTTATG GAGACCCCAA TGAAAAAGAA

3781 TCTCCCGAAC TACATTTCAA GGTAATTGAA CAATCATATA TTTAGTCCTG TAGCGATAAG

3841 AAGTTTATAA TTTATATGTT CCTATATTTT CTTCATGTTT CCTGAAATGA TTAATGAGTT

3901 GTATGTATTG ATTCATTTTA CAGTAATATA TTACTAGTAG GTTTTCACTC AAGATATTGA

3961 AATGAATTGT TGACTAATGT GCTATGATTA ATAGGTCTTT AGTCATTAGT ATGAAGTATT

4021 ATGTGACTTA AATCCTAGGT TATAAATGGT TAGCTCTCTG ATATGCAAGT AAGATCATAT

4081 GTCATAGTTG TCCTTGTTGT ATGATACCAA ACCCTAGATG GGTTCCAACG ACAAAGAACC

4141 CTGTATTCAC TGCTCTAGGT TTATGATTTT ATTCTAATAC TGTTTTTGGT ATTATTTCCC

4201 AATTTTCTAT TTATCAGATA CGGTGGAAGG GTTATGGTCC CGAGCATGAT ACATGGGAGC

4261 CCTTGGATAG TCTAAGGTAT AAAAAGTTAA TCTTTTTCAG TTTTAATTCA CCTATTATAT

4321 GAATTTGTTT TGCATTTCTC ATGTGCTCAT ATGAAATCAT GTCTTGTCTT GCCAGTTCTT

4381 GCCCACAAAA GCTGAAGGAA TTTGTCTTAA GAGGCTACAA GTCTAGAATT TTGCCGTTGC

4441 CTGTGAGTGT TTTGGTGTTT AGTATCAGCA AGTTAAATTG AACACTATTT GCATTGAAGT

4501 AATGGGTATT GTTTTCATGA CATGTAGGGA GCTGTTGATG TTGTATGTGG GGGCCCACCT

4561 TGTCAAGGCA TAAGTGGTTT TAATAGGTTT AGGAACAAGG AAGCGCCACT TGATGATCCG

4621 AAAAATAAAC AACTTGTTGT GTTCATGGAT ATTGTGTCCC ATCTTAGGCC AAAGTTTGTG

4681 TTGATGGAGA ACGTGGTTGA CCTTGTGAAG TTTGCAGGCG GTTTTCTTGG TAGATATGCA

4741 ATGGGCAGCC TTGTTGGGAT GGGTTATCAA GCAAGAATGG GAATGATGGC AGCCGGTGCA

4801 TATGGTCTGC CTCAGTTTCG CATGCGTGTT TTTGTGTGGG GTGCCCTTTC CACAGAGGTA

4861 AATTACTTCT CTTCTGAAGC TATCATTCAG TATTAATAAC TTGATAATAA ATCTGTTTTG

4921 TTGAGCTAGC TTTGCTCGTT TTTTATCCTG CCTTGTCTTT TTNTGCTGCA GAAGTTACCC

4981 TCATACCCGT TGCCAACCCA TAATGTTGTT GTCAGAGGAG TTACTCCACT TGAATTTGAG

5041 GTACGGCTTG CTAATTCTTT GGGAGTTAAA TAGTGTCCAT TTAATTTTTC TCTTCTTGGA

5101 GGGGCTTATA ATTTTTTTAT TACCTACAGG CCAATACTGT TGCATATGAA GAAGGGTGTA

5161 AATTGGATCT AAAGAAGGCA TTGGTTCTTG GTGATGCTAT CTCTGACCTC CCTCATGTAT

5221 GTAGAACTTT GATATCTCAA CCTATGATAT GTTTTATCTC TTATAATATG ATTCATGTAA

5281 TTATTATGGC TTGAGTATGC ATTATTTAGG TCGAGAATGA TGAGGGGCGT GATGAAATAC

5341 CTTATGGCTC TGAGCCTCAA ACTGAATTCC AGCGCTTCAT TAGGCAAAGA AGGGATGGTA

5401 AGCTTATAGT TTTTATTTTA CTGTTGAATG TTGGCAAAAT GCAAGTGATT GTTCTTCTTC

5461 TCCAACTTAG GCTTTTACCA CTTCTATATC ATGCTTTCAT TTGAAGGATA TAAAGTTGCA

5521 GTACAAAATA TTTATGTTTT CAATAGTATG TATGTGTTGT CACCATCCAA ATTTTATATG

5581 ATAATATGTT TTTCAGTTGA AGTATTATGT CACGATTCAC TAATTTTTCT CAAATAAACA

5641 CATTTCTAAT GCAGAAATGC CTGGTTACTT GAATGTCAAA TCTGAAGTGT TTGAACACCT

5701 GTTGTGTGAT CATCGTCCCC TTCAGCTAAA TCAGGATGAC TACGAACGTG TTTGTCAAAT

5761 ACCTAAAAAG AAGGTAGAAC CTTGTGGCTC TGGAACTGCA CAACTATGAC AGTACAAGCA

5821 AATGTATAAA TTTTCATTTT TGCAACTGTT ACCTTCATGC AGGGTGCAAA CTTCAGGGAT

5881 TTGCCAGGTG TTAGAGTTCG TCCAGACAAC AAAGTTGAAT GGGATCCTGA TGTGCCAAGA

5941 CAAAAAGTTT CTTCCGGGAA ACCTTTGGTA CAATCTGCCT TTCAAATTTC TCATCTATGT

6001 GTCTTGTTTT GTTCAGTATA CCTCATGCTC TTCATTTGCA GGTCCCAGAT TACGCTATGA

6061 CTTTTGTTAA TGGAAGCTCC AGCAAGTACG CTAGCCATTT TGATTCTATG CTTAACCTAA

6121 TATAAGTTCC AGCGTCATTG TATACTCAAA TTTCCATTCT TGCAGGCCGT TTGGACGTTT

6181 GTGGTGGGAT GAAACAGTTC CTACTGTCGT CACTCGAGCA GAGCCTCATA ATCAGGTAAC

6241 ATTTTTATTC CTCTCTCCAT GTACAATGAT TTTGCCAGTA GTGCTTCTGC TATTAACTGA

6301 AAAGAAAACT TTCTAGGCAA TCTTGCATCC TAGTCAAGAT AGAGTTCTCT CAATCCGTGA

6361 AAATGCCCGA CTGCAAGGCT TTCCTGATTA CTACAAACTT GTTGGCCCTA TAAAGGAGAG

6421 GTACCCTTAC CCCTCTCTCC TCTTCCCTTC TCTCTTGCCT CTTTTACGTA TGATTTTGAG

6481 GCGCAATTTT CATTTCGCAG ATACATACAA GTGGGGAATG CGGTGGCTGT GCCAGTTGCA

6541 CGGGCATTGG GGTTCTCTCT AGCTCGGTCA TTGAGGGGGC TGTCACATAA TGAACCAGTG

6601 TTCACCTTGC CTGAAGATTA TCCCATGATG AGAGATGTAG CTTCACCTAC TATTGACATG

6661 ATATGA

>SmCMT3 ORF sequence

1 ATGGCGAATA AGAGGAAGTC CGAATCGGCG ACGAAGGATC CGGCCGCAGC CACGGCGGAA

61 GGAAGCCGGC GGTCCAAGAA GCCAGCCTCT GCTGAATCAA CTGGGGCGGT GGACGAGGAG

121 ACAAGGATTC CAACTGTCAT AGACGACGGG AGCGAGGATG CAGGTGAGGC CCTGTTTTTA

181 GGTGAACCGG TCGCCGGCGA GGAAGCTCGG AAACGGTGGC CGCATCGATA TCAGAAAGAT

241 AATCAGTCAA CGGAAATCGT CCAAGCGAAA TGCCATTTTC TTCAAGCGAA GGTTGATGGA

301 CTGGTTTATT GCTTGGAAGA TGATGCCCAT GTCAAGGCTG AGGAAGGGAA GGAAAGTTAT

361 ATTTGCAAGA TTGTAGAATT GTTCCAAGCT GTTGATGGGA CACAAAATTT TTCAGCTCAG

421 TGGTATTATA GAGCAGATGA TACGGTTATT AAAACATGTT CTAATCTTAT AGATAACAGA

481 CAAGTATTCT TTTCTGAAGT CAAGGATGAT AATCCACTTG ACTGCCTTGT AAAAAAGCTT

541 CGAATTGTGC TTTTACCTTT GAAGGTTGAA GATCAGGTAA AGGAAAAGCT TAGATCAACA

601 CATGACTATT ACTATGACAA GATGTACTTG CTTCCATATA CTTCTTTTGT AAACTTGTCA

661 TCAGAGAGTA CTACAAACAG CAATGAATCT GGTTCTACCA TCTCTAGTGA TGCTGATGGC

721 AATTCAGTTA CACATGTATT TTCTGAGCTT GAAGGGCCAA GTGGTGAAAA AAGTCTATTG

781 GACATGTACT CTGGATGTGG TGCAATGTCG ACTGGGCTAT GCCTTGGTGC TAACAGTAAT

841 GGGGTTAAAC TTGTTACGAA ATGGGCTGTT GATCTCAACC CATATGCTTG TGAAAGCCTG

901 AAACTAAACC ATCCAGAAAC ACAGGTAAGA AATGAAAAAG CAGATGATTT TTTCCACCTG

961 CTTGTGGAGT GGGAGAAGCT TTGCACTTCC TACTTGTCCT CAGAAAACAA AGGCACATCC

1021 AGTGAGCATG TCAAGGAAGA AGTTGAAGAT GATAGTGAGG AAGATGAAAA TGGCGACGAA

1081 GATGACTCTG AAGTGTTCGA AGTTGAAGAG ATCTTGGAGG TTTGTTATGG AGACCCCAAT

1141 GAAAAAGAAT CTCCCGAACT ACATTTCAAG ATGGGTTCCA ACGACAAAGA ACCCTGTATT

1201 CACTGCTCTA GTTCTTGCCC ACAAAAGCTG AAGGAATTTG TCTTAAGAGG CTACAAGTCT

1261 AGAATTTTGC CGTTGCCTGG AGCTGTTGAT GTTGTATGTG GGGGCCCACC TTGTCAAGGC

1321 ATAAGTGGTT TTAATAGGTT TAGGAACAAG GAAGCGCCAC TTGATGATCC GAAAAATAAA

1381 CAACTTGTTG TGTTCATGGA TATTGTGTCC CATCTTAGGC CAAAGTTTGT GTTGATGGAG

1441 AACGTGGTTG ACCTTGTGAA GTTTGCAGGC GGTTTTCTTG GTAGATATGC AATGGGCAGC

1501 CTTGTTGGGA TGGGTTATCA AGCAAGAATG GGAATGATGG CAGCCGGTGC ATATGGTCTG

1561 CCTCAGTTTC GCATGCGTGT TTTTGTGTGG GGTGCCCTTT CCACAGAGAA GTTACCCTCA

1621 TACCCGTTGC CAACCCATAA TGTTGTTGTC AGAGGAGTTA CTCCACTTGA ATTTGAGGCC

1681 AATACTGTTG CATATGAAGA AGGGTGTAAA TTGGATCTAA AGAAGGCATT GGTTCTTGGT

1741 GATGCTATCT CTGACCTCCC TCATGTCGAG AATGATGAGG GGCGTGATGA AATACCTTAT

1801 GGCTCTGAGC CTCAAACTGA ATTCCAGCGC TTCATTAGGC AAAGAAGGGA TGAAATGCCT

1861 GGTTACTTGA ATGTCAAATC TGAAGTGTTT GAACACCTGT TGTGTGATCA TCGTCCCCTT

1921 CAGCTAAATC AGGATGACTA CGAACGTGTT TGTCAAATAC CTAAAAAGAA GGGTGCAAAC

1981 TTCAGGGATT TGCCAGGTGT TAGAGTTCGT CCAGACAACA AAGTTGAATG GGATCCTGAT

2041 GTGCCAAGAC AAAAAGTTTC TTCCGGGAAA CCTTTGGTCC CAGATTACGC TATGACTTTT

2101 GTTAATGGAA GCTCCAGCAA GCCGTTTGGA CGTTTGTGGT GGGATGAAAC AGTTCCTACT

2161 GTCGTCACTC GAGCAGAGCC TCATAATCAG GCAATCTTGC ATCCTAGTCA AGATAGAGTT

2221 CTCTCAATCC GTGAAAATGC CCGACTGCAA GGCTTTCCTG ATTACTACAA ACTTGTTGGC

2281 CCTATAAAGG AGAGATACAT ACAAGTGGGG AATGCGGTGG CTGTGCCAGT TGCACGGGCA

2341 TTGGGGTTCT CTCTAGCTCG GTCATTGAGG GGGCTGTCAC ATAATGAACC AGTGTTCACC

2401 TTGCCTGAAG ATTATCCCAT GATGAGAGAT GTAGCTTCAC CTACTATTGA CATGATATGA

>SmCMT3 protein sequence

1 MANKRKSESA TKDPAAATAE GSRRSKKPAS AESTGAVDEE TRIPTVIDDG SEDAGEALFL

61 GEPVAGEEAR KRWPHRYQKD NQSTEIVQAK CHFLQAKVDG LVYCLEDDAH VKAEEGKESY

121 ICKIVELFQA VDGTQNFSAQ WYYRADDTVI KTCSNLIDNR QVFFSEVKDD NPLDCLVKKL

181 RIVLLPLKVE DQVKEKLRST HDYYYDKMYL LPYTSFVNLS SESTTNSNES GSTISSDADG

241 NSVTHVFSEL EGPSGEKSLL DMYSGCGAMS TGLCLGANSN GVKLVTKWAV DLNPYACESL

301 KLNHPETQVR NEKADDFFHL LVEWEKLCTS YLSSENKGTS SEHVKEEVED DSEEDENGDE

361 DDSEVFEVEE ILEVCYGDPN EKESPELHFK MGSNDKEPCI HCSSSCPQKL KEFVLRGYKS

421 RILPLPGAVD VVCGGPPCQG ISGFNRFRNK EAPLDDPKNK QLVVFMDIVS HLRPKFVLME

481 NVVDLVKFAG GFLGRYAMGS LVGMGYQARM GMMAAGAYGL PQFRMRVFVW GALSTEKLPS

541 YPLPTHNVVV RGVTPLEFEA NTVAYEEGCK LDLKKALVLG DAISDLPHVE NDEGRDEIPY

601 GSEPQTEFQR FIRQRRDEMP GYLNVKSEVF EHLLCDHRPL QLNQDDYERV CQIPKKKGAN

661 FRDLPGVRVR PDNKVEWDPD VPRQKVSSGK PLVPDYAMTF VNGSSSKPFG RLWWDETVPT

721 VVTRAEPHNQ AILHPSQDRV LSIRENARLQ GFPDYYKLVG PIKERYIQVG NAVAVPVARA

781 LGFSLARSLR GLSHNEPVFT LPEDYPMMRD VASPTIDMI

>SmDRM1 genomic sequence

1 ATGATACTAA TGTTCCTAGT GCCTATTCAG GATGAAAATG TATCTGGAGG AGATGTTTCT

61 GATGTTGATT GGACGACAGA TGACGAGTCA GAGATTAATT TGAACCCCGT TACTACTATT

121 GTTGGCAATG AGGAGGTAAA GTTGCATTCC TGTGTATGAA GGAATCTTAG TTATTATGTC

181 TTCTCGATTG CTGCATGCAT ATCATCTTGA TTGCTGCACG CATGTCTTCT CAGTTACTAT

241 GATAGATAAA TTAACTTCTT GTTTTGTATG AAGGCTTGCT CCTCAAGAAG TGCTTCCCGT

301 TCCAAGATGT TTGATCATTT TGTTGGAATG GGTTTTTCGG ATAAATTGGT TTCAAAAGCC

361 ATTGAGGAAA ATGGTACAGA CTATTTTATA TATTAACTAA GATTTGTTTG AGATATATGT

421 TGGCTATTTT AGATTTTCAA TTGTTTATCA TGCAGGAGAA GAAGATTCAG ACTCAATACT

481 GAACTGGCTC TTAACATTCT CGGTGAGTGA TGTGTGTTAT GTTATTAAGT TAACATTGAG

541 TTTAAATGGT TCCAAATCCC TTGACATGCT ATCTCAAAAT ATTATTTGCT GTTTTCATGG

601 GATACTGGGG ACATGTAATT TCTTGTGCCT TTAATTTATC CATTATGGTT TTGAAAAGTT

661 TTTGTCATAA TATACCTTGT AACAAGAAAT GACTGCCTTT TCCCTTGTGG CCTCAGATAT

721 TTTAGGTGTT AGTTGTAGTT AATGATTTAT GTTACCTATA CAATGCTTCA ACTTTTTACT

781 TGGATGTTTT TAAAATCGTG TGAGTAACAT TACTTCAGAA GTAAATAGAA CGACTGACAA

841 TATGAAATCA AATTGGCTCT CACCTGTCCT TGTCTTCATT TTTGTTCCCA TGAAGTCAGT

901 TGACAATTAT TTAATTTTGT GAATTTTTTC AAATTCCTTC GCTAGCACAC TGTTTTTGTT

961 CCCATAACAT TCCTTGGTTA TGTATTATTT GGTTTTGCAG AGTCTTGAAG ACTCTCCTCC

1021 ACAACAGTTG AGTACAAATT TTGATCCATG CTCTTCTGAT TACAATGAGA TCTTTCCAAA

1081 TGATCTTTCA GATATGGACA GCTGGTCTGA CGATGAAATA GAAGTACGTG AGCTTTACTA

1141 GTCCTCTCAT ACTCCACATT CTATCCATAT GCTTTCAGAT GTATGCATGA GATGATATTT

1201 TGAGCAACTC TTAAGTGCAT CTGTTATTGC TTTGTTATTT AGGTCCTTTG CAAGGCTACG

1261 TTTGACCAGG AATTTGTCTT GCTTTAGTTT CTTTTCGGTA TATTATAATT TTATGTGTAA

1321 AATCAAAGAC CATATGCTGT CATTGGTGTA TACTGTTTAG CCACCTAAGT TTGCAAGATT

1381 AATTTGATAT CTGGACTTCA GTTTATGGCA CAGTATGTAA TTATATTGCA AACCACAATA

1441 ATAGAACTAA AGGATCCAGG CCATCTTTTT TTTTTTTTAC CTTTCTGAAA ATGATCCTAG

1501 TTTGGTGACT GCTTTGTGAA TACACTGTTG CTACCTGCTA CCAGTTTCTC CCATTTGCTA

1561 TAAGAAGAAA GTTATATATA TTTGAGGGAC AAAGAAAGGT ATCAAGTTGA ATATACAGAT

1621 ACTAGCCAAA AAGACAGAAA GTCGTTTTAG GAAAATTCAG CTGATCTGAC AGAATATGAA

1681 CCAGTGGGAC CGGAGATTAT ATATGTCCTA TTTACTCCAA ATCTTGAAAA TCCATGCACA

1741 TTGTCTTGAA TTCTAAATGA TTTTACTCTT ATTATTATCT AGAGCTTCAT TTGACAGTTA

1801 TTTCGTCATT AAGGTCTCTA TGCGCAGACA TGTATACTCT GCATTCAGTT ACAATCATTG

1861 ACATTTAGTT ATTATAATTT GTGACCATTT TAGTGCGTTT TATACTCCTG GCATGTAGGA

1921 CTGCTTGAAG CCCAATGGTG CTCCCAATGG TGCTGCCAAT GGTGTTTATA GATCAGAAAA

1981 GCACAAGATG TTGTTGTCAT TGGCTGCATC TGGATCATTG ACAGAGAGAG AGGAAAAATT

2041 GCTGTCCTTA GCAGATATGG GATACCCTCT GGAAGAGGCT GAAATTGCTC TGGAAAGATG

2101 TGGTGTGTAC ATTAATATGC TTCCTATTGT TTCTTTATTT TGGGGCGCAA TCTGTGATAT

2161 AAATACATTT TTGTGTTTAT CTCCTTTTAC TGCATAACCT TTTTGAAACT TGCTTTACGT

2221 TTTCTGGATA GTTTTCCTTT GATTGTGGGT AGTAATTGTT GCAGATTTAT ACGTGTACAT

2281 TTTACCTTGT CTCACAATTG TGTTACCGGC ATTGGGTTCA GTTTTGAATG TCGTGTGAAG

2341 TCAGAAATTG TTTGTGTGTT TTAATGTAGG ATGATATATG TTATATGTGC CCATGGATTA

2401 TAATCAATCA ATATAGGTCT TGATGGTTAA GTAGGAAATG GGGGTTTCAT CCTGATATTG

2461 TCCAGCTCTT GAACTTTGAT AGATTATGAG ATTCATAGAG TGTTTGAAAG ATGCTGTGTT

2521 TGTTTTCGAA CACTGTCCTT TTTTCTCATT CCTGATTTTA CTTATATTCT TGAATGCTTT

2581 TGTTTGTTCC CTCGTAGTAT TGCAACGCTG TCTGTTTGTT CTTAGTGTTT AATATTCCCC

2641 ATGTAGGTCC AGGAATAATA TTCATGGTAA TCGTTAATCT TTCTTAAATT CTCGAAGGCA

2701 TGCTTTGATC ATAGAAAGAT CTTAGAATAA TATTTACTTG CTGCTACTTT TCAGGGCCAA

2761 AAGCCTTAAT TGGTGAGTTG AGTGATTTCT TATGTGCTGC TCAAATGGCT AGAGAGGAAG

2821 ACTGTTATTT GCCTGATGAT GATCTCAAGG TCAACTTTCT ACACCATATC TTCATTTCTA

2881 TTAGATTATA GAGAATATTG CTGACCACTT TCTACCATTT CTCACAGCCA AAGCATCTTC

2941 TTAGTGGGAG CAGCAAGAGC AAGAAGAGGA AATTTCTAGA GATGAAGAAG AATGTCATTG

3001 AGGAGTCAAT TCGCCTGCCT AATCCAATGA TTGGATATGG TGTTCCTTCC ATGCAGCTAG

3061 AGCGTGTTCA TCGTTCCTTG CGAGATGATG CAAGAGCGCC CCCCTACTTC TACTATGAGA

3121 ATGTAGCCCT TACACCAAAG GGGGTCTGGG ACACCATCTC ACGGTTCCTC TACGACATCG

3181 AGCCAGAATT CGTTGACTCG AAGTTCTTCT CAGCCTGTGC AAGAAAGAGA GGCTATGTCC

3241 ACAATCTGCC CATCGAGAAC AGGTTTCCCC TCATCCCTCT CCCACCCCTG ACCATCCAGC

3301 AGGCTTTTCC CTTATCAAAA AGGTGGTGGC CTAGATGGGA TCCACGAGAC AAGCTGAACT

3361 GTATCCAGAC TGCTATAGCT AGTGCAAAAC TGACAGAGAG GATCCGCAGT GCGCTAGAGA

3421 GGGTTGAGAG TGAGGCTGAC CCACCAGAGT CAGTCCAGCG CTATGTTATG GAACAGTGCA

3481 GAAAGTGGAA TTTGGTGTGG GTGGGACGAA ACAAAGTTGC CCCCCTAGAA CCTGATGAGG

3541 TGGAAATGCT TTTGGGCTTC CCAAAGAACC ACACGAGGGG TGGTGGGATA AGTAGAACCG

3601 ACAGATACAA GTCTCTTGGA AACTCTTTTC AGGTCAGCAA TCTTTAACTC TGTTTTTTTA

3661 CTTCAGTTTT TATAATATGC CATTTTACTT ATAAGAAAAG TCCGCATCTT GATCCTTGGT

3721 ATATTGTTTT GGGTTTCAAC ATAGGTCGAC ACTGTGGCCT ATCACCTGTC TGTTTTGAAG

3781 GATATATATC CTAACGGCGT CAATGTGCTG TCCCTCTTCT CTGGTATCGG TGGAGCAGAG

3841 GTTGCACTCC ACCGACTTGG GATAAAATTG AAGAACGTTG TCTCGATTGA GAAATCTAAA

3901 GTCAACAGAG ACATAGTGAG GAGTTGGTGG GAGCAGACCA ACCAGACAGG GATGCTGATC

3961 GATTTCGACG ATGTGCAGCA GTTCGACGAC ACCAGAATAG AGCAGATTAT TGGGAGCATT

4021 GGTGGGTTCG ATCTGGTGGT TGGTGGAAGC CCTTGCAACA ATCTGGCTGG AAGCAATCGA

4081 GTCAGTCGAG ATGGTCTGGA GGGGAAGGAG TCGTCGCTGT TTTATGATTA TTTTCGTATC

4141 TTAGGTTCAG TTAAGTCGGT CATGCGCAAC AATAGGTTCG CGTAG

>SmDRM1 ORF sequence

1 ATGATACTAA TGTTCCTAGT GCCTATTCAG GATGAAAATG TATCTGGAGG AGATGTTTCT

61 GATGTTGATT GGACGACAGA TGACGAGTCA GAGATTAATT TGAACCCCGT TACTACTATT

121 GTTGGCAATG AGGAGGCTTG CTCCTCAAGA AGTGCTTCCC GTTCCAAGAT GTTTGATCAT

181 TTTGTTGGAA TGGGTTTTTC GGATAAATTG GTTTCAAAAG CCATTGAGGA AAATGGAGAA

241 GAAGATTCAG ACTCAATACT GAACTGGCTC TTAACATTCT CGAGTCTTGA AGACTCTCCT

301 CCACAACAGT TGAGTACAAA TTTTGATCCA TGCTCTTCTG ATTACAATGA GATCTTTCCA

361 AATGATCTTT CAGATATGGA CAGCTGGTCT GACGATGAAA TAGAAGACTG CTTGAAGCCC

421 AATGGTGCTC CCAATGGTGC TGCCAATGGT GTTTATAGAT CAGAAAAGCA CAAGATGTTG

481 TTGTCATTGG CTGCATCTGG ATCATTGACA GAGAGAGAGG AAAAATTGCT GTCCTTAGCA

541 GATATGGGAT ACCCTCTGGA AGAGGCTGAA ATTGCTCTGG AAAGATGTGG GCCAAAAGCC

601 TTAATTGGTG AGTTGAGTGA TTTCTTATGT GCTGCTCAAA TGGCTAGAGA GGAAGACTGT

661 TATTTGCCTG ATGATGATCT CAAGCCAAAG CATCTTCTTA GTGGGAGCAG CAAGAGCAAG

721 AAGAGGAAAT TTCTAGAGAT GAAGAAGAAT GTCATTGAGG AGTCAATTCG CCTGCCTAAT

781 CCAATGATTG GATATGGTGT TCCTTCCATG CAGCTAGAGC GTGTTCATCG TTCCTTGCGA

841 GATGATGCAA GAGCGCCCCC CTACTTCTAC TATGAGAATG TAGCCCTTAC ACCAAAGGGG

901 GTCTGGGACA CCATCTCACG GTTCCTCTAC GACATCGAGC CAGAATTCGT TGACTCGAAG

961 TTCTTCTCAG CCTGTGCAAG AAAGAGAGGC TATGTCCACA ATCTGCCCAT CGAGAACAGG

1021 TTTCCCCTCA TCCCTCTCCC ACCCCTGACC ATCCAGCAGG CTTTTCCCTT ATCAAAAAGG

1081 TGGTGGCCTA GATGGGATCC ACGAGACAAG CTGAACTGTA TCCAGACTGC TATAGCTAGT

1141 GCAAAACTGA CAGAGAGGAT CCGCAGTGCG CTAGAGAGGG TTGAGAGTGA GGCTGACCCA

1201 CCAGAGTCAG TCCAGCGCTA TGTTATGGAA CAGTGCAGAA AGTGGAATTT GGTGTGGGTG

1261 GGACGAAACA AAGTTGCCCC CCTAGAACCT GATGAGGTGG AAATGCTTTT GGGCTTCCCA

1321 AAGAACCACA CGAGGGGTGG TGGGATAAGT AGAACCGACA GATACAAGTC TCTTGGAAAC

1381 TCTTTTCAGG TCGACACTGT GGCCTATCAC CTGTCTGTTT TGAAGGATAT ATATCCTAAC

1441 GGCGTCAATG TGCTGTCCCT CTTCTCTGGT ATCGGTGGAG CAGAGGTTGC ACTCCACCGA

1501 CTTGGGATAA AATTGAAGAA CGTTGTCTCG ATTGAGAAAT CTAAAGTCAA CAGAGACATA

1561 GTGAGGAGTT GGTGGGAGCA GACCAACCAG ACAGGGATGC TGATCGATTT CGACGATGTG

1621 CAGCAGTTCG ACGACACCAG AATAGAGCAG ATTATTGGGA GCATTGGTGG GTTCGATCTG

1681 GTGGTTGGTG GAAGCCCTTG CAACAATCTG GCTGGAAGCA ATCGAGTCAG TCGAGATGGT

1741 CTGGAGGGGA AGGAGTCGTC GCTGTTTTAT GATTATTTTC GTATCTTAGG TTCAGTTAAG

1801 TCGGTCATGC GCAACAATAG GTTCGCGTAG

>SmDRM1 protein sequence

1 MILMFLVPIQ DENVSGGDVS DVDWTTDDES EINLNPVTTI VGNEEACSSR SASRSKMFDH

61 FVGMGFSDKL VSKAIEENGE EDSDSILNWL LTFSSLEDSP PQQLSTNFDP CSSDYNEIFP

121 NDLSDMDSWS DDEIEDCLKP NGAPNGAANG VYRSEKHKML LSLAASGSLT EREEKLLSLA

181 DMGYPLEEAE IALERCGPKA LIGELSDFLC AAQMAREEDC YLPDDDLKPK HLLSGSSKSK

241 KRKFLEMKKN VIEESIRLPN PMIGYGVPSM QLERVHRSLR DDARAPPYFY YENVALTPKG

301 VWDTISRFLY DIEPEFVDSK FFSACARKRG YVHNLPIENR FPLIPLPPLT IQQAFPLSKR

361 WWPRWDPRDK LNCIQTAIAS AKLTERIRSA LERVESEADP PESVQRYVME QCRKWNLVWV

421 GRNKVAPLEP DEVEMLLGFP KNHTRGGGIS RTDRYKSLGN SFQVDTVAYH LSVLKDIYPN

481 GVNVLSLFSG IGGAEVALHR LGIKLKNVVS IEKSKVNRDI VRSWWEQTNQ TGMLIDFDDV

541 QQFDDTRIEQ IIGSIGGFDL VVGGSPCNNL AGSNRVSRDG LEGKESSLFY DYFRILGSVK

601 SVMRNNRFA

>SmDRM2 genomic sequence

1 ATGTGTGATG TAGTGGACAT TTCAGATGAT GAGGATTCCT TTTTGCTGGA AAATGATGCT

61 GGAGTTATAC CCAAGGATGA GAATTTGGAT TATGATTTAC CACCGCCGAA AACATTCTCA

121 ATGCCAAGGG AGGTATGATT CTCCTTAATA AAATACAGCA AATTCGTTAA GGATTGGGGG

181 AAATCATGTC TTGGTTAAGT TTAATGCTGA AAGAGTGAGA TTGATTCCAC TACCGTTGTA

241 GCTTTAGGAC TTGAAGGACA TGTTTTGCAT CTTTTATTTA CTGAAGAATT CATCGTCTTC

301 TTTGGCTGCA CTGTAGGAGG ATGGCGCAAG CTCGTCGGGG ACTAATTTAA GATCATCGTT

361 CATTGGAATG GGGTTTGCAC CAGCTCTCGT CGACAAAGCA ATCAAGGAAA AGGGTTGGTG

421 TAGCTTACTC CTAGACAGTT TTGTGGCTTT TGTAATGTTT GAGTTGATAT TAATCTGAGC

481 TTGGCTGTTA ACCATTAATT TTCTTTTAGA GAGTCTCCAA ACCATTGTAC TGGCAATTTC

541 AACTAAATCA AGATTTTGAA ATAGTTATCT TTTATGTATT ATAGTTACAC ATCACTATTC

601 GTTTTGAATA ATGACACATC AGGAAACTAA TATCTACCTT ACATTTTAGG TGAAGAAAAC

661 AGCGAACTGA TACTGGAAGC TCTTTTTTCA TATGATGTAA GTGTTAATCC TCGCAAGATA

721 AAAGTGATAA ACTACCATAT TTTCATACTT CTGGATTTTG GGTTAACATG ACACACAAGT

781 TTGTCTTTCA CCATGCCATA TGTAATTTTC CTTTGAATTA GACTGTTGCC CATTTAAACT

841 CAATCATTTG ACTTTTTATC CTTACATCTA TCATGTTGAT AAGTTATGGC TGACTGCTGG

901 TATTTCTATG AAAGACTTCT TTCCTTTTGC AGCTTATAAT GCTTGAATTA GACATGTTTT

961 TCCCAATTGA AGTTAGCCTT TTCCTTTGAC TAGGATGCTG AATTGTGCCA TGCATATCAA

1021 CACAGAATTT AGCCTTTTTT GTTTAACTCT GCGTATGAAG TTATGCTGTC CATCCCTAAT

1081 TGAACAGGCC CTTCAGAAAC CAAAGAATGA AGTTCTTGAT AATAGTTTTT CTGGGGAATG

1141 CAGTGATAGT TTAGCTGGAA ACCTTCGTGC TGAAGAGGTA TGCTTGTTCT CAGTTGTCTC

1201 TATGAGAATC CTTTCAATCA TATCTACACT TGAGTCATTG ACAAATCCAA CATGTTGTCT

1261 CTGGTAACTT GCAAAGGATG AATTTTCTGT GCAAATAAAG ACTTCCATTC AAAAACTGCA

1321 TTCAGTTTTT CTGATTTATG ATTGTTAATG CTAGAGATCC TTACTAGATC TGTGTTCTTA

1381 TCTCAGTCTT TCCTACTTTT TTTGATCAAT TGTTTGTTAG AGCATGTAGC TTACATTATA

1441 TCTATTCTTA ATGTTCATAA TATGGGTGTC TATGAAGTGA ATCACTTATC CTTCTGATTT

1501 TATTAGTGAA CCTTCTTTAG TTAAAATACT CGCCCCTGAG TTAACTAGTG AACCGTAGTT

1561 TTCTGGTCAT TCTTTTTATC TACTTTTAGT TCCCACCCAT AGATAAAGTT TAATATTATC

1621 TGTGTACTTG CAGAATGCAC ATTTAACTGT TCTCATCTCT AGTCTTGAGC TTTCTTTTTG

1681 AATGTTTTAG TTTCTGTTTT CTGAAGTATT TTGAAATATA CCAGGATTTT CACAAATCGG

1741 AGTCATCGGA TTCTCTTGGT AGATTGTTTG CTGAAGGTAC AAAGAAGAAT GCCTATGTTG

1801 AGGCTGATAT TCCTTTAAAA GTGGTAAATA TCAATATTTT TCCATTACTT GTTTCATTCA

1861 CATGTCATAA CCATAGCATA AGGTGAAATT TCCCTTTAAT CAACTGACGT AGTAGGCATT

1921 GCCTTGCCGC TGCATAGTTT TATCAGCTTT CTGTGTGACG TGTGCATGTT GTTTTTGTTG

1981 AAAGCCTTTT GCTAACTATG GCTTGAACTT TTCTGTACTG TACTTCTGAA GTCTTATACT

2041 CCCTCCAGGC TCCAGCCCAT AAGAAAGCAT CACATTTTCC ATTTTCGTCC GTCCCATAAG

2101 AAAGTATCAC TTCTATTTTG GGACATGGCC CCACTTTCTC TTTTTAACCA CAACCACTCA

2161 TTTCTACATA ACTCTACAAT CAATTCAACC ATAAACACTC ATTTCCACTC AATACATTAC

2221 CTATCAACCT TTTCTTAAAA CCCGTGCCAT CCCATATGCG ATACTCTCTT ACGGGATTGA

2281 GGGAGTAACG ATTATTAGGT TCTCAGTCTA CTGTCTTTCT TACAGTTGCA TATGTCTATT

2341 TATGTAATAC TAGTTGCTGG CTGCATCAAT TTGCAAACCA ACCCAAATTT ATGAGTAGAA

2401 TAACTGTGCT AAGGAAGTAC TAGTGATTTG ATGCCTGCTA ATCAGTCATC TAATTTCCAA

2461 AGCCTTGGGC TTTTTGTGTA TTATTGTGCT CAAAATGTCT TATTCATGAA AATTTATAGC

2521 ATCTTATTTT CTACAAATCA AATGATTAGA AACAATATTT CTGTTACTTT GATGGAAATT

2581 ATTTTAATTG AAGAGTCAAC TGTGTTCGGA ATTACATGCT TTCCATTATA CTTAATTTAT

2641 TGTCAACTAC TTCGCTGATT TGTCATTGGA AATTTTCTTT CTATGTTTTT ACCTCCCAAG

2701 GATGAAATTT ACTCGTTTGC TTGCATCGAA TAAGTCAGAT TTCCTCTCCT ATACTCTGAT

2761 GATTCTGATT TTGTTGCGAT AACTGTTCTT TCAGGAACTT GATGTTGGTG ATAGTGTCAT

2821 TGATGAGAAA ACGGCATCGT TGCTAAAAAT GAATTTCTCT TTGGATGAAG TTGGATTTGC

2881 TATGCAAAGA CTTGGTTCGT GGAATTTTCC CCATTCCAGC CTTGAGCTTC TAATTATGTG

2941 AAACATGCCT CATCTGTAAA TCTAGTGAAG TGTGCACTCA GTGTCTCCCT GTAATTAGTA

3001 ATTTATAACA TGAGAATGAG CTGTGCTACT CTTGACTAGG TCATTATGCT ACACTTGGTC

3061 ATTTGCTGTA TTTGATAATC TGTCAAATAA TTTCTGTAGC TTCCTAAGTT TGTCAGTCAT

3121 GCTTAATATT TCTAATATAG TTGTTTTTAA TCATCATTTT GTTGATTGAA GTTTTCTTTC

3181 ACGGTTTCAC CTTATTCCTA TTGATTATTA AATTTACAGC TAATTCTACT GCATGGAAAG

3241 TTAGCTGTAA AGTTACCTCA CAGTTTGCTC AAACAAGAAA AAGAAAAATT ACGTCATAGT

3301 TGTTGCCTAA CTGGTTACTT TGGTGTTGGA TCACAGGTAA AAATGCTGCT ATTGGTCAAC

3361 TAATGGACTT CATCTTCGCT GCACGGATGG CTAGAAAGTA TGAAAAGGAC ACAAATAATT

3421 CAATTCCTGG AAATGAGGAA ACCGAAAAGG TCTTAGATGA CATCTTTTTT ATAGTTTGTT

3481 AGATAGATCT GATGATTTCA GATGGTTAGG TACTTAGGTT ACATGGTTGG GGATGCCACC

3541 ATAGATTTTG TTTGCTACTT TATTTCTATT ATGCTCACTA TCTTTTATGT TTTGGAATCT

3601 TATGTCTTAA ACTCAGTTGC AAATTTGCAG GATTGCAACA ATGAGGTATT ATTTGGTATT

3661 ATGGAGAAGT CACTTCAACT GCTCGAAATG GGCTTTACAG AAAACGAAAT TTCCGCAGCT

3721 TTTGAGAGAT GTGGTGAGAT GATTATGCAA ATTGACAAAA ATATGCATTT AGTAAGTTCA

3781 CCAGATGGTG CTAGAATAGT CGTATCCAAA GTAAGATGGT TTTCCAAATG TGATGTTGGA

3841 GTCCTATTCT ATTTGATACC CCATGCATTG TTTCTCACCA TGAGCATCTG ATGTTTATTT

3901 TGAGGATGCA TTGTTTGTGA GTGAGGTAAT GAAGGACTTA AAGTAAACTG GAAAATAAAA

3961 TTTTTGGAGT CTCTAGGAGA TTGAACAATA ATGATACTAG TTGTAATAGA TAGATGCGTG

4021 CAATCAATTG AAAGTTTTTG CAATTTCTTG GTTAAATTCA AAACGAAAGT GCTACCGGAT

4081 GTAGGTGTTT CCTACATTAG ATCCACACCT GCACCGTTGA TGGACCATTC CATTAAGTCC

4141 CAAGTAAATA GGATTACAAC AATTGAATTT CAGGTTATTG TGTTCATATT AGTATTGTTA

4201 CCACCAATAT TTCTTATTCT CTCCGAGTTA GTAATTTCAT GATCTCTCAC CATGTTATCA

4261 TATCTTAGGT TCGGAAGCAC CCGTGACACT ACTTGCTGAA TCGATTGTTA CAGGTGGTAC

4321 TTGCCCATTA CCAGATAAGG TCTACATCAT TACTAATACG ATGATTCTTC ATTTGTAGAT

4381 CCACAGAACT GCTTGTTATG GAAGGATCAC AACTTCTGTT TATTGATCTT CATTTTGCAG

4441 TATTCCTCAA CTCTTTTGAG CACCTCAGGT GTCCGAAGGA GTTTTCAGTC TTCGAAAAGA

4501 AAGATGGAAG ATTACAACTC GATCTGCATT AAAACTGAAG AATACAGTTC AGATGTAGCA

4561 TCTCAAGTTA GGACTTCTGA CTTGCTGGAG AAATTGAAAG GCAAGATGCC AGAAGTAGAC

4621 ATAGATGAAC CAAACAATTT CAAGAAACCA AAGGAAGAAT TTATGGAAGA CTACCCTTCA

4681 GTTGGTAATT CTGCGGCAGT CCTACAGAGA CGGGGTGGCT GTTTTGCAAG CATTGATGAT

4741 GATAGGAAGC CCACAATATC CTTACCCAAT GCTTGCAGAA GTCTTAGTGA TGGGGTGGCC

4801 AAACCCCCGT ATTTCCTTTA TGGAAACGTC ACAAGTCTAT CTCCGGGCTC TTGGGCCAAA

4861 ATATGTAAGT TCTTGTACTC AGTTCAGCCG GAGTTTGTGG ATTCTCATTT GTTCTCAGCA

4921 TTGAACAGAA AGGAAGGCTA TGTCCATAAC CTCCCAAGTG AAGATCGGTT TCACATTTTT

4981 CCAAAGGGAC CAATGACTAT TGCAGAAGCC ATTCCATATG CCAAAAAATG CTGGCCGTCA

5041 TGGGATAATC GGAAGCAACT AAGCTCCATC ACTTGTGATA CGACTGGATT ACCCTATCTC

5101 TGTGAGAAAC TAGGAAGAAT GCTGGCTGAA TGCAACGGGC CTCCATCTGC AGAACTACAA

5161 ACAAGGATTC TCCAACAATG TGTGGCCAAG AATCTTGTTT GGGTAGGCAA GCAGAAGCTA

5221 GGTCCACTCG AACCTGAACA TCTGGAGCAC ATAATGGGCT ATCCGCAGCA TCACAGTCGA

5281 ATTGCTGGAT ATAGCTTGAC AGAGAGGCTG AAATCGCTGA AGCTCTCGTT CCAGACAGAC

5341 ACGTTGGGGT ACCATCTCTC AGTGTTGAAG CGCTTGTTTC CAAGCGGGGT GACAGTGCTG

5401 TCGTTCTTCA GTGGTATTGG TGGAGCAGAG GTTGCTCTAC ACCGGCTTGG GATTCGTCTG

5461 AAAGGGGTGG TCTCGGTGGA GCCGTGTGAG ACGAAGCGCA GGATCATGAA GAAGTGGTGG

5521 GAGAAGTCAG CGCAGAGTGG AGAGTTGATT CAGCTTGAGA GCATCAACAA ATTAACAAGC

5581 AACAAGCTTG AAGATCTGTT GAAGAAGTTG AAGGGATTTG ATTTGGTTAT ATGCCAGAAT

5641 CCATATTCTG GTGCAGAATC AGATAGTGAT AGCTTGGGCG GTTCAGATTT CTCAATGTTT

5701 GTAGAATTTG TAAGAATTAT GCAGCGTGTA AGGTCTACGT CTGTGGATGA CTTTTAA

>SmDRM2 ORF sequence

1 ATGTGTGATG TAGTGGACAT TTCAGATGAT GAGGATTCCT TTTTGCTGGA AAATGATGCT

61 GGAGTTATAC CCAAGGATGA GAATTTGGAT TATGATTTAC CACCGCCGAA AACATTCTCA

121 ATGCCAAGGG AGGAGGATGG CGCAAGCTCG TCGGGGACTA ATTTAAGATC ATCGTTCATT

181 GGAATGGGGT TTGCACCAGC TCTCGTCGAC AAAGCAATCA AGGAAAAGGG TGAAGAAAAC

241 AGCGAACTGA TACTGGAAGC TCTTTTTTCA TATGATGCCC TTCAGAAACC AAAGAATGAA

301 GTTCTTGATA ATAGTTTTTC TGGGGAATGC AGTGATAGTT TAGCTGGAAA CCTTCGTGCT

361 GAAGAGGATT TTCACAAATC GGAGTCATCG GATTCTCTTG GTAGATTGTT TGCTGAAGGT

421 ACAAAGAAGA ATGCCTATGT TGAGGCTGAT ATTCCTTTAA AAGTGGAACT TGATGTTGGT

481 GATAGTGTCA TTGATGAGAA AACGGCATCG TTGCTAAAAA TGAATTTCTC TTTGGATGAA

541 GTTGGATTTG CTATGCAAAG ACTTGTTGCA AATTTGCAGG ATTGCAACAA TGAGGTATTA

601 TTTGGTATTA TGGAGAAGTC ACTTCAACTG CTCGAAATGG GCTTTACAGA AAACGAAATT

661 TCCGCAGCTT TTGAGAGATG TGGTTCGGAA GCACCCGTGA CACTACTTGC TGAATCGATT

721 GTTACAGGTG GTACTTGCCC ATTACCAGAT AAGTATTCCT CAACTCTTTT GAGCACCTCA

781 GGTGTCCGAA GGAGTTTTCA GTCTTCGAAA AGAAAGATGG AAGATTACAA CTCGATCTGC

841 ATTAAAACTG AAGAATACAG TTCAGATGTA GCATCTCAAG TTAGGACTTC TGACTTGCTG

901 GAGAAATTGA AAGGCAAGAT GCCAGAAGTA GACATAGATG AACCAAACAA TTTCAAGAAA

961 CCAAAGGAAG AATTTATGGA AGACTACCCT TCAGTTGGTA ATTCTGCGGC AGTCCTACAG

1021 AGACGGGGTG GCTGTTTTGC AAGCATTGAT GATGATAGGA AGCCCACAAT ATCCTTACCC

1081 AATGCTTGCA GAAGTCTTAG TGATGGGGTG GCCAAACCCC CGTATTTCCT TTATGGAAAC

1141 GTCACAAGTC TATCTCCGGG CTCTTGGGCC AAAATATGTA AGTTCTTGTA CTCAGTTCAG

1201 CCGGAGTTTG TGGATTCTCA TTTGTTCTCA GCATTGAACA GAAAGGAAGG CTATGTCCAT

1261 AACCTCCCAA GTGAAGATCG GTTTCACATT TTTCCAAAGG GACCAATGAC TATTGCAGAA

1321 GCCATTCCAT ATGCCAAAAA ATGCTGGCCG TCATGGGATA ATCGGAAGCA ACTAAGCTCC

1381 ATCACTTGTG ATACGACTGG ATTACCCTAT CTCTGTGAGA AACTAGGAAG AATGCTGGCT

1441 GAATGCAACG GGCCTCCATC TGCAGAACTA CAAACAAGGA TTCTCCAACA ATGTGTGGCC

1501 AAGAATCTTG TTTGGGTAGG CAAGCAGAAG CTAGGTCCAC TCGAACCTGA ACATCTGGAG

1561 CACATAATGG GCTATCCGCA GCATCACAGT CGAATTGCTG GATATAGCTT GACAGAGAGG

1621 CTGAAATCGC TGAAGCTCTC GTTCCAGACA GACACGTTGG GGTACCATCT CTCAGTGTTG

1681 AAGCGCTTGT TTCCAAGCGG GGTGACAGTG CTGTCGTTCT TCAGTGGTAT TGGTGGAGCA

1741 GAGGTTGCTC TACACCGGCT TGGGATTCGT CTGAAAGGGG TGGTCTCGGT GGAGCCGTGT

1801 GAGACGAAGC GCAGGATCAT GAAGAAGTGG TGGGAGAAGT CAGCGCAGAG TGGAGAGTTG

1861 ATTCAGCTTG AGAGCATCAA CAAATTAACA AGCAACAAGC TTGAAGATCT GTTGAAGAAG

1921 TTGAAGGGAT TTGATTTGGT TATATGCCAG AATCCATATT CTGGTGCAGA ATCAGATAGT

1981 GATAGCTTGG GCGGTTCAGA TTTCTCAATG TTTGTAGAAT TTGTAAGAAT TATGCAGCGT

2041 GTAAGGTCTA CGTCTGTGGA TGACTTTTAA

>SmDRM2 protein sequence

1 MCDVVDISDD EDSFLLENDA GVIPKDENLD YDLPPPKTFS MPREEDGASS SGTNLRSSFI

61 GMGFAPALVD KAIKEKGEEN SELILEALFS YDALQKPKNE VLDNSFSGEC SDSLAGNLRA

121 EEDFHKSESS DSLGRLFAEG TKKNAYVEAD IPLKVELDVG DSVIDEKTAS LLKMNFSLDE

181 VGFAMQRLVA NLQDCNNEVL FGIMEKSLQL LEMGFTENEI SAAFERCGSE APVTLLAESI

241 VTGGTCPLPD KYSSTLLSTS GVRRSFQSSK RKMEDYNSIC IKTEEYSSDV ASQVRTSDLL

301 EKLKGKMPEV DIDEPNNFKK PKEEFMEDYP SVGNSAAVLQ RRGGCFASID DDRKPTISLP

361 NACRSLSDGV AKPPYFLYGN VTSLSPGSWA KICKFLYSVQ PEFVDSHLFS ALNRKEGYVH

421 NLPSEDRFHI FPKGPMTIAE AIPYAKKCWP SWDNRKQLSS ITCDTTGLPY LCEKLGRMLA

481 ECNGPPSAEL QTRILQQCVA KNLVWVGKQK LGPLEPEHLE HIMGYPQHHS RIAGYSLTER

541 LKSLKLSFQT DTLGYHLSVL KRLFPSGVTV LSFFSGIGGA EVALHRLGIR LKGVVSVEPC

601 ETKRRIMKKW WEKSAQSGEL IQLESINKLT SNKLEDLLKK LKGFDLVICQ NPYSGAESDS

661 DSLGGSDFSM FVEFVRIMQR VRSTSVDDF

>SmDNMT2 genomic sequence

1 ATGGAGGGTT TAAGTAGAGA GACAGAGAAG CCATGGCGCG TACTCGAATT CTACAGTGGC

61 ATAGGTGGAT TGGTATGCTT GTTTATTGTC GTGTTTAATT GGAACTCTTT TTGTGCTTAA

121 TTTTGATTTC GATTGCGGTA GCGATATTCA TTGATGGAGG CGGGCGTAGA TGCTGTGGTA

181 ATCGAAGCAT TTGACATCAA TGACGTTGCA AATGATGTTT ACGAGCACAA TTTCGGCCAC

241 CGTCCGCATC AGGTAGCATC TTAAGATTAG CTGAAATATG AGTTTATATA TGCTATTCAT

301 GCATGTAGTG ATTCATTTAG TTTGCTAGTT TTGTATTTTC AGTATGATAA TTAGTTAGAG

361 ATGTTTTTGC ATTAGATGAT TTAGGTTGGT GTGTGTGTGT GTGTGAATTT GATATTAATT

421 CATGCTAATA TTCGCTTGTG GTGTTGGACG TTTATTTTGG TGTGCTTGTA GGGAAATATT

481 CAGACCTTGA GTGCTGTTAA CCTCGACAGA TATGGGGCTG ATGCATGGCT TTTGTCTCCT

541 CCTTGCCAAC CTTACACAAG ACAAGGTGGT TGATTTCTCG TTCATTAAAT TTTTCTGTGC

601 TATGCAGATT GTATGAGCAT ATCTACTTGT GTTGACAGTG TTGTTATAGC TTCTTAAAAT

661 ATTGTTTATT AAGGTCTCCA GAAGGGTTCA AATGACGCTA GAGCTTCTTC ATTTTTGAAG

721 ATTCTTGAAC TAATACCACA ATGTTCACGA CCCCCAGTGA TGCTATTTGT GGAGAATGTT

781 GTTGGATTTG AGGTTTGTGG TTTATGTATG TCTTTCATTA AGTAAAAAAG ATTTTAGTAT

841 TGAATTTGTT GAACCACCTT TCTTATGAAA TGACAACTTC GTAATTGCAA TTTTTTTCTA

901 GAAATTTGTT TGGGTTTGGA GTTGTATGTG GATGTAGGTT TTTCTTTTTA TTTTCCTATG

961 CCTTTTCGTT TCTTTTTCGA GGAGACATAT TGTATAAGTT TTTTATTAAA ACCCATGATC

1021 TTACTATTAT TTTTGTTTAA CAAGTCATTC ATATATACTT TCTCTCTATT CATTTTGTAT

1081 CTTGTTTCTA TTTCCTTTTG CAGACATCTG ATACACATGA AAAGATGATC ACCATGTTGC

1141 GAGAAAATCA TTTTGACACA CAAGAATTCA TTCTAAGTCC TTTGCAATTT GGTGTGCCAT

1201 ATTCTCGGCC TCGCTATTTT TGCCTGGTCC ACCTCTCTCT CTCTCTCTCT CTCTCTGTCC

1261 CCAGTATATA CACCCACATA ATTTATGTTT GTCCATTTAC TTTTGTATAT CCAGTGCATT

1321 CACATGCATT CTCTCCATCT CTTCATTATG CATCTGCCTG TAAAGTTTGT ACCTAGCACG

1381 TGATATGGAA TCTTAACGCA GTTATACATA GAATCTGCAG CCTGAATAGA GAAAGTACTC

1441 TATATATTTA TTCGGCTTTC TTTACTCTTA TTGGTTGTTC GATATATGTT TCTAATCCAT

1501 TGTGAACTAC AGGCAAAGCG GAAACCTATA TATTTCCAGA AGGCACAGTT CAATAATGCG

1561 CTTCTTTGGT GCCCACGCCC TATACTTGGG TATGAGGAAA GCATGGTGAC AAGTGAAGAT

1621 GCTGAATCAC ATACATATTG GAATAAATTA CTTGAAACTA CTCTACCTAT ATGTGATTTT

1681 TTGGAATCAA GTGAAGTTAT TTTAGAATCG AGCTTAACTA AAATATCTGA GGATACTGAA

1741 GAAGAAAGTG AACTTGCTGC TGACCCTTCA AAACTGTTTT TAGTTCCATC TAGCCAGATA

1801 GAAAGATGGG GGAGTGCCAT GGGTATGCTC ACCTTTTCCA GCCTTGATAC TTCTTTACCA

1861 TTTTGTTCAC ATTTACTTTT GTTTTTGTAA CTCTTATACC ATTAAATTGG TCTACTTCTT

1921 TACCATTTTG TTCGCATTTA TTTCTGTTTC TGTAACTCTT ATACCATTAA ATTGGTTCAA

1981 TGATTGATTT AGATATTGTT TTCCCCGAAT CAAGACGCTG TTGTTGCTTC ACCAAAAGTT

2041 ACTTTCGATA CGTGAAGGGG ACTGGTTCCG TTTTGGCTAC CTGTCCCGCA AGTCAGCTAC

2101 AGGTACACAC CAATTTTTAT TGCAAATATA TCTATTTAGA TATAAGCACA TACAATATCA

2161 GCAGTTCCTA CCTTATGTCC TGAGAGCCTT TGAATTGTTC TTTATATATT CCTGATATTC

2221 CATGAGTGTC AATTATTGAT ATGTTCCTTT ACTTACAATT AACTTTCTGG AGATGAATAT

2281 TAATCTGAAT ATGGAATCCA AATTCTTTTG CACTGCATAT CCATGATGAT TTCATTTGAA

2341 TTTTCCCTAT GCATGCATGC CGACATAAGT TTGTACTATT AAATTTTCCA TGTGTAAGAG

2401 TGAAATATGT TGATGCTTGG TTATCCATGT TTATGTCTTG CGAAATACTC GAGGATGAAA

2461 ACTCTTTTGC TGACCTTGTG AAAACTTCTG CAGGTATGAT CACGTTCCTC ATCACGAAGA

2521 GTTCTCACTT GTGCACTGTT TTTTGAGTGT TCCTGATTCT GATATTGCCA AAGACATTAA

2581 TTTTCTTTCT TCAATATTAA AGCAATCATA TCACTTATCA TATTATGCAG GGAAAGAAGA

2641 AAATTGAATT GCCTTCGTTG CAGGAGCTTC AACTTAGATA CTTTACGCCC AGGGAGGTGC

2701 ACATTTTTCA CTCTCATACA TCAGTTTGTT TTTCAGCTCC TTTTGCATTA TTACTTGTTC

2761 AATTTTGTTC TCTCAAGTGT TTTCTCATCA ATGTCACTGT CAGGTCGCCA ATTTCCACTC

2821 TTTTCCGAAG GAGTTCCAAT TTCCACAACA TGTTAGCCTT CGTCAACGGT GCGCCTTACA

2881 TTTCTTGTAT TGTTGCTTGA CATAATATGC AAAAGCAGGC ATCTTTGAGG AGGCTATTTC

2941 TCACTCATGT GGAACCTAAT GAAAGAATTA TGGACATATA GAAGTGACTT AAATCAGGTG

3001 TTGCCTCTCC AACTGCTTAG GAATTTTAAA ATAATATATA GCGAATAATT TCATCCTAGG

3061 GCAGTTGGTT GCAGTTGGCC TCTGGAACTT TGCATGAGAA TATATATGAT CTATTTTTTG

3121 GATATCATAT ATGTTGGTTT TTGTGTTATT GGTTTGTTTG TTGGTTATGT GCATGTTCTT

3181 AACTAGAGAT GGTTATGTGC AGTTACGCGT TGCTGGGAAA CAGTCTGAGT GTTGGAGTCG

3241 TGGCACCACT TTTGCGCTAT CTATTTACTC AGCAATCATC ATAG

>SmDNMT2 ORF sequence

1 ATGGAGGGTT TAAGTAGAGA GACAGAGAAG CCATGGCGCG TACTCGAATT CTACAGTGGC

61 ATAGGTGGAT TGCGATATTC ATTGATGGAG GCGGGCGTAG ATGCTGTGGT AATCGAAGCA

121 TTTGACATCA ATGACGTTGC AAATGATGTT TACGAGCACA ATTTCGGCCA CCGTCCGCAT

181 CAGGGAAATA TTCAGACCTT GAGTGCTGTT AACCTCGACA GATATGGGGC TGATGCATGG

241 CTTTTGTCTC CTCCTTGCCA ACCTTACACA AGACAAGGTC TCCAGAAGGG TTCAAATGAC

301 GCTAGAGCTT CTTCATTTTT GAAGATTCTT GAACTAATAC CACAATGTTC ACGACCCCCA

361 GTGATGCTAT TTGTGGAGAA TGTTGTTGGA TTTGAGACAT CTGATACACA TGAAAAGATG

421 ATCACCATGT TGCGAGAAAA TCATTTTGAC ACACAAGAAT TCATTCTAAG TCCTTTGCAA

481 TTTGGTGTGC CATATTCTCG GCCTCGCTAT TTTTGCCTGG CAAAGCGGAA ACCTATATAT

541 TTCCAGAAGG CACAGTTCAA TAATGCGCTT CTTTGGTGCC CACGCCCTAT ACTTGGGTAT

601 GAGGAAAGCA TGGTGACAAG TGAAGATGCT GAATCACATA CATATTGGAA TAAATTACTT

661 GAAACTACTC TACCTATATG TGATTTTTTG GAATCAAGTG AAGTTATTTT AGAATCGAGC

721 TTAACTAAAA TATCTGAGGA TACTGAAGAA GAAAGTGAAC TTGCTGCTGA CCCTTCAAAA

781 CTGTTTTTAG TTCCATCTAG CCAGATAGAA AGATGGGGGA GTGCCATGGA TATTGTTTTC

841 CCCGAATCAA GACGCTGTTG TTGCTTCACC AAAAGTTACT TTCGATACGT GAAGGGGACT

901 GGTTCCGTTT TGGCTACCTG TCCCGCAAGT CAGCTACAGG GAAAGAAGAA AATTGAATTG

961 CCTTCGTTGC AGGAGCTTCA ACTTAGATAC TTTACGCCCA GGGAGGTCGC CAATTTCCAC

1021 TCTTTTCCGA AGGAGTTCCA ATTTCCACAA CATGTTAGCC TTCGTCAACG TTACGCGTTG

1081 CTGGGAAACA GTCTGAGTGT TGGAGTCGTG GCACCACTTT TGCGCTATCT ATTTACTCAG

1141 CAATCATCAT AG

>SmDNMT2 protein sequence

1 MEGLSRETEK PWRVLEFYSG IGGLRYSLME AGVDAVVIEA FDINDVANDV YEHNFGHRPH

61 QGNIQTLSAV NLDRYGADAW LLSPPCQPYT RQGLQKGSND ARASSFLKIL ELIPQCSRPP

121 VMLFVENVVG FETSDTHEKM ITMLRENHFD TQEFILSPLQ FGVPYSRPRY FCLAKRKPIY

181 FQKAQFNNAL LWCPRPILGY EESMVTSEDA ESHTYWNKLL ETTLPICDFL ESSEVILESS

241 LTKISEDTEE ESELAADPSK LFLVPSSQIE RWGSAMDIVF PESRRCCCFT KSYFRYVKGT

301 GSVLATCPAS QLQGKKKIEL PSLQELQLRY FTPREVANFH SFPKEFQFPQ HVSLRQRYAL

361 LGNSLSVGVV APLLRYLFTQ QSS
